# Direct Recycling of Mixed‐Oxide Cathodes: Balancing Cost, Performance and Environmental Trade‐Offs

**DOI:** 10.1002/advs.202519076

**Published:** 2026-02-16

**Authors:** Evgenii Beletskii, Elizaveta Evshchik, Anna Shikhovtseva, Valery Kolmakov, Andrey Popov, Svetlana Eliseeva, Lyubov Shmygleva, Yurii K. Gun'ko, Valentin Romanovski

**Affiliations:** ^1^ MllT Key Laboratory of Critical Materials Technology for New Energy Conversion and Storage School of Chemistry and Chemical Engineering Harbin Institute of Technology Harbin China; ^2^ Federal Research Center of Problems of Chemical Physics and Medicinal Chemistry Russian Academy of Sciences Chernogolovka Moscow Russia; ^3^ Institute of Chemistry St. Petersburg State University St. Petersburg Russia; ^4^ Department School of Chemistry CRANN and AMBER Research Centres Institution Trinity College Dublin College Green Dublin 2 Ireland; ^5^ Department of Materials Science and Engineering University of Virginia Charlottesville Virginia USA

**Keywords:** battery recycling, environmental impact, lithium‐ion battery, relithiation, sustainable development, techno‐economic analysis

## Abstract

We compare solid‐state relithiation (SSR), hydrothermal relithiation (Hydro), molten salt thermochemistry (MST), electrochemical (EC), and chemical (Chem) methods using harmonized techno‐economic (Group I), electrochemical (Group II), and environmental/toxicological (Group III) metrics. SSR/Hydro occupies a leading position in Group I. EC combines low energy (143.0 kJ·g^−1^) with higher material cost (147.7$·kg^−1^) at the laboratory scale, yielding 71.9 pts, while Chem remains competitive (77.8 pts) despite elevated 201.4$·kg^−1^ and moderate energy consumption of 333.7 kJ·g^−1^. In Group II, MST and SSR lead (64.0 and 61.1 pts), followed by Chem, Hydro, and EC. In Group II, Chem/MST shows the best rate capability recovery, EC—cycling stability recovery, MST—capacity recovery. Group III follows the energy consumption track. The CO_2_ emission declines as follows: EC < Chem < MST < Hydro < SSR, with method toxicity near moderate hazard for SSR/MST/Hydro/EC and highly reactive / highly toxic for Chem. Integrated performance for Groups I–III is close for methods with energy control in the range of 60–65 points (SSR, Hydro, MST, EC), while Chem leads (72.3 points). For Ni‐rich cathodes, transferable methods should include a brief high‐temperature step. EC/Chem can only be used for mild regeneration.

## Graphical abstract

Direct regeneration routes for mixed‐oxide lithium‐ion battery cathodes are systematically compared using harmonized techno‐economic, electrochemical, and environmental metrics. Solid‐state, hydrothermal, molten salt, electrochemical, and chemical relithiation methods are benchmarked to identify performance, cost, and sustainability trade‐offs. The analysis establishes transferable criteria for selecting scalable regeneration strategies.

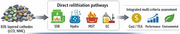

## 1 Introduction

Lithium‐ion batteries (LIBs) play a crucial role in the transition to a sustainable energy future, providing energy storage solutions for a wide range of applications, from electric vehicles to grid energy systems. With a global market capacity projected to reach about 1700 GWh by 2025 [1, 2, 3, 4, 5, 6, 7, 8, 9, 10], the increasing use of LIBs presents significant challenges related to resource management and environmental impact, particularly concerning the growing number of end‐of‐life (EOL) batteries. These spent batteries contain valuable metals as well as potentially harmful substances, underscoring the need for effective recycling strategies to ensure resource conservation, environmental protection, and the promotion of sustainable development [11, 12, 13].

Among the various cathode materials used in LIBs, mixed oxide‐based compounds such as lithium cobalt oxide (LCO), lithium manganese oxide (LMO) and nickel–manganese–cobalt oxides (NMСs) are commonly utilized due to their high energy density and performance characteristics [14, 15, 16]. However, EOL batteries, containing these materials, cause both environmental and resource‐related concerns [17, 18]. These batteries still contain valuable metals that can be recycled and reused, but their degradation during use, including loss of electrochemical properties and structural changes in the cathode materials, requires efficient recycling processes. Direct regeneration of these cathodes has emerged as a promising approach to restore their structure, composition, and electrochemical properties, effectively addressing the environmental and resource challenges associated with their disposal.

The relithiation process (regeneration or direct recycling), which involves restoring lithium in degraded cathode materials, plays a critical role in this direct regeneration. The key to successful relithiation comes from overcoming the problems associated with irreversible lithium loss and the formation of undesirable phases during long‐term cycling. Various relithiation methods, including electrochemical [19, 20, 21], chemical [22, 23, 24], solid‐state relithiation [25, 26, 27], molted salt thermochemistry [28, 29, 30] and hydrothermal [31, 32, 33] techniques, have been developed to restore the electrochemical activity of these mixed oxide cathodes and improve their total performance. The development of these diverse approaches aims to provide less resource‐intensive, cost‐effective, and environmentally friendly solutions for the recycling of mixed oxide‐based cathodes.

**FIGURE 1:**
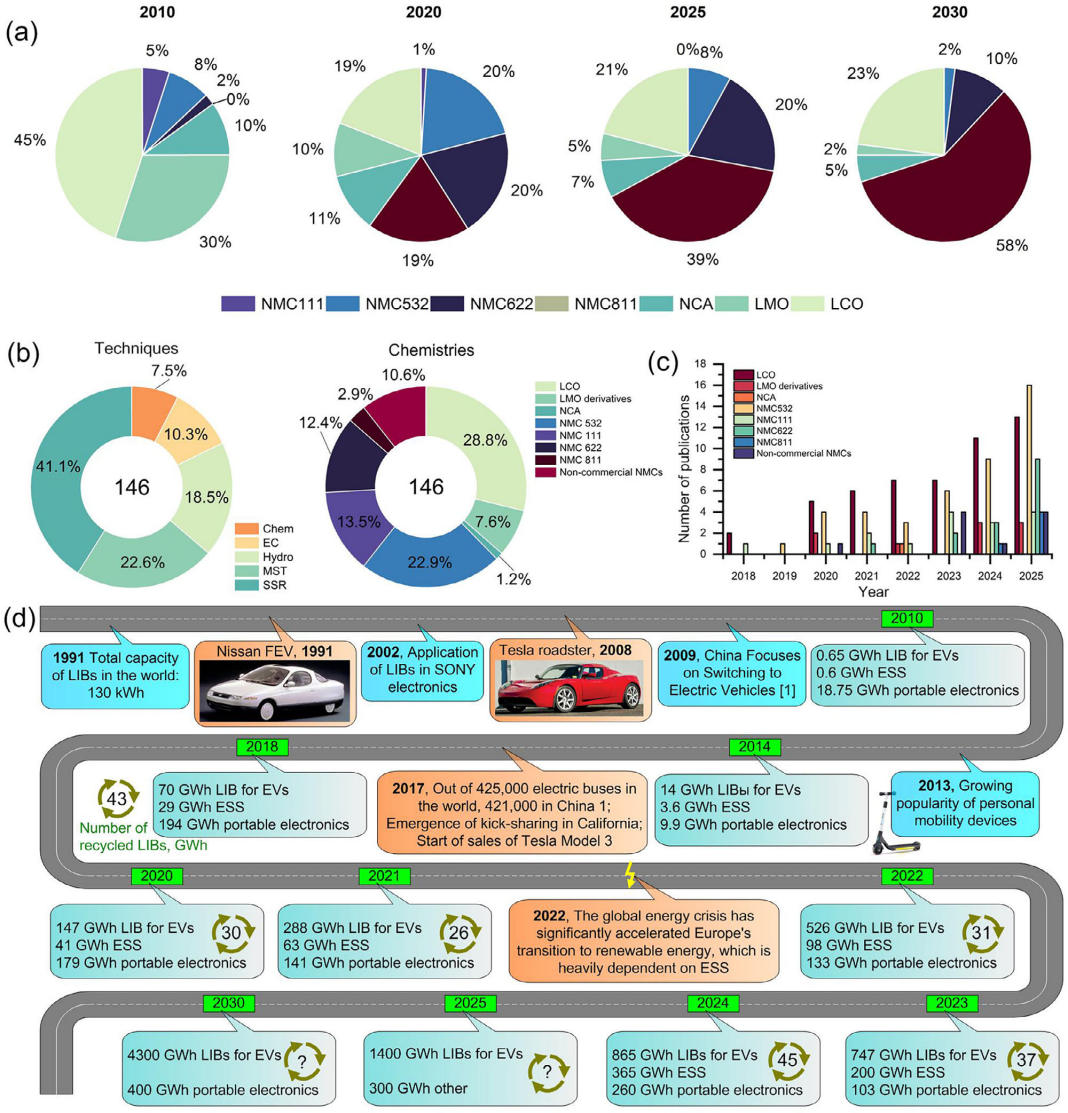
(a) Evolution of Lithium‐Ion Battery Cathode Chemistry Composition from 2010 to 2030. Compiled based on [34, 35, 36, 37, 38, 39, 40]; (b) Distribution of relithiation techniques (SSR—solid‐state relithiation; Hydro—hydrothermal relithiation; MST—molten salt thermochemistry; Chem— chemical relithiation; EC—electrochemical relithiation) and chemistries in research. The figure presents the percentage share of different relithiation methods studied in the literature, with sintering (SSR) being the most investigated technique. It also highlights the prevalence of various cathode chemistries in relithiation studies, with lithium cobalt oxide (LCO) and nickel‐manganese‐cobalt (NMC 532) variants being the most frequently examined materials. Compiled from the 146 publications used in this paper for analysis (Table S1); (c) Publication trends in relithiation research (2018–2025). The figure shows the annual number of studies dedicated to different recycled chemistries, highlighting the dominance of LCO cathode material. Compiled from the 146 publications used in this paper for analysis (Table S1). (d) Evolution of lithium‐ion battery production, recycling trends, and projected waste accumulation. The infographic illustrates key milestones in LIB development, the growing demand for electric vehicles and portable electronics, and the widening gap between LIB waste generation and recycling capacity. The projected battery waste by 2030 highlights the urgent need for improved recycling technologies and industrial‐scale implementation. Compiled from reports [1, 3, 4, 5, 6, 7, 8, 9, 10, 41] and publications [42, 43, 44, 45, 46, 47].

An updated market composition forecast of LIB chemistries from 2010 to 2030 (Figure 1a) provides critical context for evaluating the relevance of relithiation strategies. The data show a dramatic change in cathode composition: in 2010, the market was dominated by LCO (45%) and LMO (30%), and by 2030, NMC811 is projected to account for 58% of total production with LCO and LMO declining to 23% and 2%, respectively. These trends suggest a strong industrial transition toward Ni‐rich chemistries, particularly NMC811, due to their superior energy density and lower cobalt content. This evolution directly informs the direction of relithiation research, indicating that methods must increasingly be optimized for Ni‐rich systems rather than previous materials like LCO or LMO.

The importance of relithiation research has been steadily growing, as evidenced by an increasing number of publications in recent years (Figure 1b,c). Between 2018 and 2025 (Figure 1b), studies on relithiation using sintering (solid‐state relithiation or solid‐state reaction, SSR) as the main process have dominated, accounting for approximately 41% of the publications, while hydrothermal (Hydro) and molten salt thermochemistry (MST) methods also demonstrated significant academic interest (22.6% and 18.5%, respectively). Meanwhile, electrochemical (EC) and chemical (Chem) approaches received relatively less attention, with only 10.3% and 7.5% of the studies focusing on these methods. However, it is noteworthy that most published studies apply each relithiation method to only one cathode composition, typically LCO or NMC532, and that there is limited systematic cross‐chemistry validation. While the underlying relithiation mechanisms are broadly transferable, their applicability to emerging Ni‐rich materials is not well understood. In particular, although NMC811 is expected to become one of the main commercial chemistries, research into its direct regeneration has only recently begun to increase: the number of published studies on this topic rose from one before 2024 to four by 2025 (Figure 1c). This growth signals rising interest in overcoming the challenges associated with Ni‐rich systems. Nevertheless, the transferability of methods to different chemistries for NMC811 and related high‐Ni cathodes remains a critical research gap.

Despite growing interest in regeneration research, efforts to recycle LIBs on an industrial scale have not kept up with the rapidly increasing volume of battery waste. The current industrial recycling capacity is vastly insufficient when compared to the projected LIB waste (Figure 1d). There is exponential growth in LIB market capacity and linear growth in recycling capacity, so that between 2020 and 2024, the former increased from about 367 to 1460 GWh, respectively, and the latter from 30 to 45 GWh, respectively. This drawback represents a significant risk to global sustainability efforts, as improper disposal of LIBs can lead to serious environmental consequences, including the leakage of hazardous materials and inefficient resource utilization. The widening gap between the accumulation of spent LIBs and their recycling capabilities points to the urgent need for strategic advances in recycling methodology and industry adoption.

To tackle this problem, it is essential to critically assess and prioritize research areas that can be feasibly scaled up for industrial applications. Some methods currently being studied in academia may not be suitable for large‐scale processing due to economic or environmental constraints, while others hold great promise. Therefore, understanding which recycling techniques should be developed further and which should be given less attention is of primary importance. We employ a multi‐criteria evaluation system that enables transparent and quantitative comparison of the methods presented in terms of cost, electrochemical performance, and environmental impact. Crucially, to avoid overinterpreting the heterogeneous and incomplete data presented in the literature, we complement the central trends with explicit levels of reliability (median and IQR, 95% confidence intervals of the median, and leave‐one‐out sensitivity, ΔLOO). This system is not intended to replace comprehensive technical‐economic (TEA) or life cycle (LCA) analyses but serves as an intermediate tool that systematizes disparate experimental data into a single numerical scale. Thus, it allows identifying the most effective publications for each relithiation method highlighting development trajectories and, in the case of comparable overall assessments, serves as a basis for developing hybrid strategies by combining the advantages of different approaches. Based on this approach, we propose a structured roadmap that links academic research with industrial implementation. This roadmap combines mechanistic research, multi‐criteria assessment, LCA/TEA analysis, pilot and demonstration validation, and ultimately integration into the circular economy. By placing the assessment system within this broader perspective, we aim to bridge the gap between laboratory research and practical industrial solutions for sustainable cathode regeneration.

## 2 Methodology

The detailed description of the score estimation approach undertaken in our publication was provided in our previous work [48]. All estimation changes made according to layered oxides state of art are specially discussed in relevant sections. All results of the criteria assessment are collected in Table S1.

### 2.1 Direct Production Costs Criteria (Group I)

To evaluate relithiation techniques from a production cost perspective, as discussed in our previous publication [48], the cost structure associated with electrode materials and battery production criteria is essential [48]. The key parameters considered were: material cost, energy consumption, capital expenditure for key equipment, process duration, and space requirements. A scoring methodology was implemented, utilizing reference points to normalize and benchmark the selected indicators across all publications examined. Specifically, the minimum observed value for each parameter was assigned a score of 100 points, representing optimal performance, while the maximum value received a score of 0 points. Parametric values were then converted into corresponding scores, using an empirical equation, facilitating a comparative assessment of relative performance. Critically, distinct reference points were established for each individual parameter to ensure accurate and context‐specific evaluation.

**1. Material cost**. Material cost is a crucial factor influencing the economic viability of lithium‐ion battery recycling processes. To estimate material costs, the price of each compound was obtained from the Fisher Scientific website [49]. Of each compound, all search results corresponding to the chemical formula and the purity grade reported in the original publication were examined. From these, the reagent with the lowest price per kilogram at the specified purity was selected to represent the minimum achievable laboratory‐scale cost. This approach ensures consistency across the evaluated methods and avoids overestimation caused by random selection or transient price fluctuations. To enhance data visualization and presentation, a boundary value of $1000∙kg^−^^1^ was applied. This threshold effectively encompasses over 90% of the publications, which exhibit material costs below this value, while simultaneously preventing a disproportionate influence from the approximately 10% of publications reporting significantly higher material costs.

To convert the cost of input materials presented in the publication to a scoring system, Equation (1) can be used:

(1) $$S_{M}=\left\{\begin{array}{c} 0\;\;\;\;\;\;\;\;\;\;\;\;\;\;\;\;\;\;\;\;\;if\;C_{M}=1000\;\$\cdot kg^{-1}\\ 100\;\;\;\;\;\;\;\;\;\;\;\;\;\;\;\;\;\;\;\;\;\;if\;C_{M}=0\;\$\cdot kg^{-1}\;\\ \frac{1000-C_{M}}{1000}\cdot 100\;\;\;\;\;\;\;\;\;\;\;\;\;\;\;\;\;\;otherwise\; \end{array}\right.$$

where *S_М_* is the score of the article's technique related to material costs (*pts*), *C*_M_ is the cost of chemicals per 1 kg of cathode active material, CAM ($·kg^−1^), 1000$·kg^−1^ is the maximum material cost.

**2. Energy consumption**. To accurately assess the energy consumption associated with a given relithiation technique, it is imperative to account for not only the energy directly consumed by the primary relithiation reaction itself but also the energy expended during pre‐ and post‐processing steps. In general, the following processes represent the most energy‐intensive aspects of cathode active material treatment: sintering, drying, electrolysis, hydrothermal treatment, and molten salt thermochemistry.

Unit operations such as reagent mixing, washing, filtration, and decantation were excluded from the energy consumption calculations due to their comparatively negligible contribution to the overall energy demand. It is important to acknowledge that the resulting energy consumption estimates are inherently approximate, as they rely on modeling the CAM treatment processes based on descriptions provided by various authors in their respective publications. While differences may arise between the idealized modeling conditions and specific experimental parameters reported in the selected literature, this approximation remains sufficient for a meaningful comparative analysis using the proposed scoring scale.

**Sintering**. To calculate the energy consumption of the sintering process, Equation (2) for the relationship between heat and the heat capacity of CAM was used:

(2) $$E=c_{CAM}\cdot \left(T_{2}-T_{1}\right)\cdot NEE$$

where *T*_1_ and *T_2_* is the room temperature (20°C) and operating temperature, *с_CAM_*—the specific CAM heat capacity at the temperature *T_2_* and *NEE* (*normalized excess energy*) is the coefficient that relates the energy required for sintering to the energy actually consumed in the process. For annealing in muffle furnaces, *NEE* can be assumed to be equal to 400 [50].

**Drying**. To estimate the energy consumption (J·g^−^^1^) during cathode active material drying, we considered the energy required for heating the wet CAM and evaporating the residual solvent. The solvent content was assumed to be 10 wt%. The energy consumption was then calculated using Equation (3):

(3) $$E=c_{CAM}\left(T-20\right)+0.1\cdot Q_{r}$$

where *T* is the drying temperature (°C), *c_CAM_* is a CAM heat capacity (J· K^−1^·g^−1^), 0.1 is a solvent fraction in CAM, *Q_r_* is the specific heat of vaporization (J·g_solvent_^−1^) that depends on solvent nature. The choice of a solvent‐to‐CAM ration threshold value of 0.1 was necessitated by the need for a rough estimate of energy consumption that would nevertheless allow a meaningful comparative analysis of the various techniques presented in the literature. This approach is similar to pre‐assessment in laboratory experiments where accurate determination of moisture content is not critical. Furthermore, this value is supported by thermogravimetric analysis (TGA) data, wherein the mass loss observed during the heating of various oxides prior to decomposition typically does not exceed 10% [51, 52, 53]. More rigorous energy consumption assessments can be conducted at a later stage through techno‐economic analysis (TEA), which accounts for both the intrinsic properties of the recycled material and the key parameters of the technological process.

**Electrolysis**. To calculate the energy consumption of electrochemical relithiation, Equation (4) for calculating the amount of electrical energy passing through an electrical circuit was used:

(4) $$E=\frac{I\cdot V\cdot t}{m_{CAM}}$$

where *I* is the current (A), *V* is the voltage of the power source or the difference in electrode potentials between the working electrode and the counter electrode (V), *t* is the electrolysis time (s), and *m_CAM_* is the mass of cathode active material (CAM) subjected to electrolysis (g).

**Hydrothermal treatment or molten salt thermochemistry**. For hydrothermal treatment and molten salt thermochemical processes, energy consumption calculations were performed using Equations (5) and (6). In this context, “solvent” refers to both the liquid medium in which the cathode active material is dispersed during hydrothermal or molten salt thermochemical treatment.

Given the use of a significant excess of solvent in these reactions, the majority of thermal energy is consumed in heating the solvent medium, allowing the heat absorbed by the active material itself to be considered negligible. Therefore, the calculated heat input for these processes considers the temperature‐dependent specific heat capacity of the solvent. The resulting energy consumption values are then normalized with respect to the mass of the final relithiated cathode active material product.

(5) $$E=c_{solvent}\cdot \left(T_{2}-T_{1}\right)\cdot \frac{m_{solvent}}{m_{CAM}}$$

Conversely, in cases where the solvent is present in substoichiometric or comparable quantities relative to the cathode active material, a comprehensive energy consumption calculation must account for the heat absorbed by both components of the mixture, incorporating the temperature dependence of their respective specific heat capacities.

(6) $$E=c_{solvent}\cdot \left(T_{2}-T_{1}\right)\cdot \frac{m_{solvent}}{m_{CAM}}+c_{CAM}\left(T_{2}-T_{1}\right)$$

where *T*_1_ and *T_2_* is the room temperature (20°C) and operating temperature, *c_solvent_* is the average heat capacity of solvent (J· K^−1^·g^−1^) between *T*_1_ and *T_2_* [54].

Using the idealized scenario outlined in the Material Costs analysis, we defined two benchmark values for energy consumption scoring:

– **100 points** corresponds to zero energy consumption (0 kJ·g^−1^), representing the optimal case.
– **0 points** corresponds to the maximum reported energy consumption in the literature (1000 kJ·g^−^^1^, see Data S1), representing the least efficient case.

The conversion from specific energy consumption values to scores was performed using the following empirical equation (Equation 7):

(7) $$S_{E}=\left\{\begin{array}{c} 0\;\;\;\;\;\;\;\;\;\;\;\;\;\;\;\;\;\;\;\;\;\;if\;E=1000\;\mathrm{kJ}\cdot g^{-1}\\ 100\;\;\;\;\;\;\;\;\;\;\;\;\;\;\;\;\;\;\;\;\;\;if\;E=0\;\mathrm{kJ}\cdot g^{-1}\;\\ \frac{1000-E}{1000}\cdot 100\;\;\;\;\;\;\;\;\;\;\;\;\;\;\;\;\;\;otherwise\; \end{array}\right.$$

where *S_E_* is the score of the article's technique related to energy consumption (*pts*), *E* is the energy consumption of 1 g of CAM (kJ·g^−1^).

In those publications that did not provide the detailed hydrothermal treatment parameters necessary to accurately calculate energy consumption, we assumed that these energy consumptions did not exceed the average energy consumption for the hydrothermal treatment stage reported in other publications with similar conditions. This assumption is based on the expectation that the median value, representing the most frequently observed energy consumption, provides a reasonable estimate even in the absence of complete experimental details. The median energy consumption for the hydrothermal stage was determined to be 4.1% of the sintering stage energy consumption.

**3. The Key equipment costs, Process duration, and Space requirement** criteria were assessed using the same methodology as previously described in our earlier publication [48], with the following modifications to the scoring scale:

1. Key equipment costs: 0 points for $5000 and 100 points for $0;
2. Process duration: 0 points for 100 h. and 100 points for 0 h.

The process duration or repairing time criterion represents the total technological time required for the completion of cathode relithiation, including all auxiliary pre‐ and post‐treatment stages such as precursor preparation, washing, drying, and annealing.

**4. Integrated score of Group I**. To calculate and compare the total average score of different relithiation techniques for Group I, a ranking method using weighted coefficients (significance coefficients) was used in Equation (8), where the sum of the coefficients equals 1.

(8) $$S_{Group\;I}=\sum_{i=1}^{n}S_{i}\cdot k_{i}$$

where *S*_i_ is the rank of the object according to the first, second,..., *n*th criteria; *n* is the number of criteria used for averaging, and *k_i_* is the significance coefficient of each criterion as a fraction of unity.

Thus, the following significance coefficients were used: 0.68 for materials, 0.026 for energy, 0.13 for equipment, 0.1 for labor (i.e., duration), and 0.062 for land and building rent (i.e., space) [48]. Weighting coefficients are selected based on an analysis of the cost structure and impacts in technical and economic models (EverBatt, BatPaC) and independent studies. A detailed justification is provided in Section S1.

### 2.2 Performance Criteria (Group II)

Consistent with our prior methodology [48], the score evaluation of cathode active materials was conducted using three distinct criteria groups. However, to more effectively address the inherent challenges in assessing relithiation techniques, a modified approach was implemented for defining the boundaries of these criteria groups, as detailed in subsequent sections.

**1. Rate Capability**. C‐rate data were digitized from each publication and plotted as a function of capacity vs. charge/discharge rate (Figure S1). These data were then analyzed using an empirical equation derived from Tian's work [55, 56] to obtain *τ* values, where *τ* represents the characteristic time associated with the material's power performance. A smaller *τ* value signifies superior rate capability. A detailed explanation of the relationship between *τ* and the physical properties of the materials and electrodes is available in Tian's work [55, 56]. In our previous study, the improvement in rate performance was quantified by dividing the *τ* value of the pristine cathode material by the *τ* value obtained after relithiation, yielding a parameter denoted as *⟨τ⟩*. While this approach allowed for objective assessment of rate capability, the data representation was occasionally disrupted because the criterion boundaries were defined by the maximum and minimum values of *⟨τ⟩*. To enhance data presentation, we modified our approach proposed in the present work. The current criteria are based on the difference between the characteristic time before and after relithiation (*τ_after—_τ_before_)*. Given that smaller *τ* values indicate better rate capability, an ideal material would exhibit *τ* = 0. Therefore, a material achieving this ideal would have a value of *τ_after—_τ_before_* ***=—*** *τ_before_*, which was chosen as the upper boundary for the scoring criteria, assigning a score of 100 points. Conversely, a material exhibiting minimal improvement in rate capability (*τ_after_* ≈ *τ_before_*), resulting in a difference approaching zero, would receive a score of 0 points.

This modification allows the evaluation system to remain adaptive and self‐consistent as new publications emerge, even if future results fall outside the ranges currently specified. Unlike normalization based on fixed standards for defining criterion boundaries, the flexible boundary approach dynamically incorporates new data and maintains internal comparability between heterogeneous studies.

The resulting dataset was then converted into a score using empirical Equation (9):

(9) $$S_{c-rate}=\left\{\begin{array}{c} 0\;\;\;\;\;\;\;\;\;\;if\;\tau_{after}-\tau_{before}=0\\ \;\;\;\;\;\;\;\;\;\;\;\;\;100\;\;\;\;\;if\;\tau_{after}-\tau_{before}=-\tau_{before}\\ \frac{\tau_{after}-\tau_{before}}{-\tau_{before}}\cdot 100\;\;\;\;\;\;\;\;\;\;\;\;\;\;\;\;\;\;otherwise\; \end{array}\right.$$

where *S_c‐rate_* is the score of the article's technique related to the rate capability criteria (*pts*), *τ_after_ and τ_before_* represent the characteristic times of the CAM after and before relithiation, respectively.

**2. Cyclic Stability**. As a key parameter objectively correlated to CAM cyclic performance, a degradation constant *k_c_* can be applied. According to the empirical equation applied in a publication [57], a degradation constant *k_c_* can be calculated as follows:

(10) $$Capacityretantion=1-k_{c}\cdot N^{0.5}$$

where *k*_c_ is the cell capacity degradation constant, *N* is the number of charge/discharge cycles.

To evaluate the cyclic stability of CAM before and after relithiation, data from the selected publications were compiled into Table S1, and analogously to the rate capability assessment, the difference between *k_c(after)_ and k_c(before)_* was used as the key parameter to be converted to the scoring system.

Utilizing empirical Equation (10), the resulting dataset was converted into a score system:

(11) $$S_{c\mathrm{ycling}}=\left\{\begin{array}{c} 0\;\;\;\;\;\;\;\;\;\;if\;k_{c\left(after\right)}-k_{c\left(before\right)}=0\\ 100\;\;\;\;\;\;\;\;\;\;\;\;\;\;\;\;\;\;\;\;\;\;if\;k_{c\left(after\right)}-k_{c\left(before\right)}=-k_{c\left(before\right)}\\ \frac{k_{c\left(after\right)}-k_{c\left(before\right)}}{-k_{c\left(before\right)}}\cdot 100\;\;\;\;\;\;\;\;\;\;\;\;\;\;\;\;\;\;otherwise\; \end{array}\right.$$

**3. Capacity**. While the two criteria described previously reflect the impact of the relithiation technique on the rate capability and cyclic stability of the CAM, they do not explicitly account for changes in the material's capacity. Consequently, significant improvements in rate capability and cyclic stability would be awarded high scores irrespective of the CAM's absolute capacity. This omission could lead to an underestimation of the overall performance enhancement achieved through relithiation, resulting in an inaccurate assessment of the technique. To address this limitation, in our work [48], we proposed the introduction of an additional criterion linked to the CAM capacity change, denoted as *S_capacity_*, which quantifies the difference in capacity before and after relithiation. To isolate the impact of relithiation on capacity, comparisons were conducted at the lowest reported current densities, minimizing the influence of rate limitations. An ideal relithiation technique would fully restore the CAM's capacity, particularly for highly degraded CAM exhibiting discharge capacities approaching 0 mAh∙g^−1^.

To evaluate the capacity criterion, the following Equation (12) was used:

(12) $$S_{capacity}=\left\{\begin{array}{c} 0\;\;\;\;\;\;\;\;\;\;\;\;\;\;\;\;\;\;\;\;\;\;if\;Q_{after}-Q_{before}=0\;\\ 100\;\;\;\;\;\;\;\;\;\;\;\;\;\;\;\;\;\;\;\;\;\;if\;Q_{after}-Q_{before}=Q_{theoretical}\\ \frac{Q_{after}-Q_{before}}{Q_{theoretical}}\cdot 100\;\;\;\;\;\;\;\;\;\;\;\;\;\;\;\;\;otherwise\; \end{array}\right.$$

where *Q*_before_ and *Q*_after_ are the LFP capacity before and after relithiation. 170 mAh·g^−1^ is the capacity difference for the ideal relithiation technique.

**4. Integrated score of Group II**. To calculate and compare the total integrated score for the Group II relithiation techniques, a ranking method employing weighted coefficients (significance coefficients) was used, as described in Equation (13), where the sum of the coefficients equals 1.

(13) $$S_{GroupII}=\sum_{i=1}^{n}S_{i}\cdot k_{i}$$

where *S*_i_ is the rank of the object according to the first, second,..., *n*th criteria; *n* is the number of criteria used for averaging, and *k_i_* is the significance coefficient of each criterion as a fraction of unity.

The significance coefficients were selected based on the premise that rate capability, cyclic stability, and material capacity represent the most critical performance attributes for battery applications. Materials exhibiting superior performance across these parameters are generally considered optimal. Consequently, a significance coefficient of 1/3 was assigned to each parameter, reflecting their equal weight in determining the overall material performance score.

### 2.3 Environmental Impact Criteria (Group III)

The environmental impact criterion (Group III) was calculated in accordance with our previous publication [48]. We considered two primary sources of CO_2_ emissions during the recycling process. The first source is material‐related, stemming from CO_2_ release during chemical reactions involved in recycling or from the oxidation of organic molecules into CO_2_ under environmental conditions following leakage. The second source is energy‐related, correlating with the recycling energy consumption, which was converted to kg CO_2_eq∙kg^−1^ using an emission factor of 0.417 kg CO_2_eq∙kWh^−1^ [58]. We used the values calculated directly in Group I (Material Costs). The resulting values were then used to generate a GHG emissions score based on the energy‐related criterion with the boundary conditions defined by Equation (14):

(14) $$S_{Group\;III}\left\{\begin{array}{c} 0\;\;\;\;\;\;\;\;\;\;\;\;\;\;\;\;\;\;\;\;\;\;if\;\varepsilon =120\;kg_{CO_{2}e}\cdot kg_{CAM}^{-1}\\ 100\;\;\;\;\;\;\;\;\;\;\;\;\;\;\;\;\;\;\;\;\;\;if\;\varepsilon =0\;kg_{CO_{2}e}\cdot kg_{CAM}^{-1}\\ \frac{120-\varepsilon }{120}\cdot 100\;\;\;\;\;\;\;\;\;\;\;\;\;\;\;\;\;\;otherwise\; \end{array}\right.$$

where *S_GHG_* is the score for the technique based on GHG emissions associated with material and energy consumption (*pts*), and ε is the total CO_2_ emission (kg∙CO_2_e∙kg^−1^) per kg of CAM recovered.

In addition to the primary scoring criteria, we conducted a supplementary toxicity scoring *S_tox_* based on the GreenScreen Hazard Benchmark concept, as detailed in Supporting Information. This indicator provides a preliminary view of potential chemical hazards but is not integrated into the main evaluation framework due to the fragmented nature of available data. Instead, it serves as an auxiliary case study illustrating how relithiation techniques may differ in toxicological impact.

### 2.4 Total Average Score

After calculating all three groups of criteria to determine the most optimal the specific synthesis technique, the scores for all three groups of criteria were averaged. The following Equation (15) was used:

(15) $$S_{sum}=\frac{S_{GroupI}+S_{GroupII}+S_{GroupIII}}{3}$$

where *S*_GroupI_ is the total average score of relithiation techniques for Group I (calculated via Equation (9)), *S*_GroupII_ is the total average score of relithiation techniques for Group II (calculated via Equation (13)), *S*_Group III_ is the GHG criterion (calculated via Equation (14)).

As a result of this averaging process, each synthesis approach is characterized from three complementary perspectives: direct production costs, electrochemical performance, and environmental impact. The publications that achieve the highest scores across these categories are selected as the most promising candidates for a subsequent techno‐economic assessment, including scale‐up feasibility and cost estimation of battery energy at the system level.

### 2.5 Statistical Evaluation of Box‐Slot Robustness

For each relithiation method and group of criteria, the number of publications included (N), individual metric values, and grouping labels were compiled into a single analytical dataset. Due to the frequent abnormality and asymmetry of empirical performance indicators presented in studies on EC and Chem methods, we chose statistical measures that are independent of distribution for this analysis. This approach avoids the excessive influence of outliers and assumptions about distribution inherent in classical parametric statistics [59].

1. **Median and interquartile range**. We adopted the median as the primary measure of central tendency because it is robust in the presence of skewness and extreme values and is more resistant to breakdown than mean‐based metrics. We used the interquartile range (IQR), which is defined as the difference between the 75th and 25th percentiles, to quantify dispersion in each group [60].
(16) $$\mathrm{Median}=Q_{50};\;\mathrm{IQR}=Q_{75}-Q_{25}$$

This non‐parametric summary captures the core distributional features without reliance on mean‐variance assumptions common in parametric techniques.

1. **95% Confidence Interval for the median (95% CI)**. To derive reliable uncertainty estimates for the median of each box‐slot parameter, we implemented a bootstrap resampling procedure with 10^4^–10^5^ iterations. Bootstrap methods involve repeatedly resampling the dataset with replacement and computing the statistic of interest to build an empirical sampling distribution [61]. The 95% confidence interval (CI) for the median was determined using the 2.5th and 97.5th percentiles of the bootstrap distribution. This method is suitable for non‐normal and irregular empirical distributions.
2. **Leave‐One‐Out Sensitivity (ΔLOO)**. In order to evaluate the sensitivity of the median estimates to the individual data points, we computed a leave‐one‐out (LOO) median shift, which is defined as follows:
(17) $$\Delta LOO=\frac{1}{N}\sum_{i=1}^{N}\left|Median_{\left(-i\right)}-Median_{full}\right|$$

here: *Median_(−i)_* represents the median computed from the full dataset after excluding the *i‐th* entry, consistent with jackknife/LOO resampling principles; *Median_full_* is the median from the original full dataset; *N* is the number of observations.

This metric quantifies the average influence of each individual observation on the central tendency estimate, providing insight into the robustness of box‐plot parameterization against the removal of single studies.

1. **Interpretation**. By combining non‐parametric central tendency, dispersion, uncertainty intervals, and sensitivity metrics, this framework captures multiple dimensions of reliability in box‐slot assessments. Median and IQR characterize the core statistical properties, bootstrap‐derived CIs quantify uncertainty without normality assumptions, and ΔLOO identifies the influence of individual studies on the robustness of aggregated results. Collectively, these measures ensure that the patterns reported in subsequent sections reflect consistent behavior in literature data rather than artifacts of sampling variability.

## 3 Results and Discussion

### 3.1 Review of Publications from an Economic Efficiency Perspective

To compare the economic efficiency of different relithiation strategies, we compiled the material costs, energy consumption, and other processing parameters for all eligible publications, converting them into a Group I score (*S_Group I_*) using the methodology described in Section 2. Additionally, we quantified the statistical robustness of each method group using non‐parametric metrics (median, interquartile range (IQR), 95% confidence interval of the median, and a leave‐one‐out sensitivity test (ΔLOO), which provide a simple sensitivity analysis with respect to data incompleteness and outliers. We systematically categorized publications according to the types of reagents applied and indicated the material cost ranges and energy consumption with median values as well as the values of *S_Group I_* and IQR that quantifies the spread of the central 50% of the data and therefore captures the intrinsic variability of reported costs across studies and reflects how sensitive each method is to changes in reporting practices and thus provides a more reliable measure of the robustness of performance. A summary of cathode active materials, reagents, and key processing parameters across different relithiation methods are provided in Table 1 and Table S2. Table 1 shows the variety of CAM compositions, lithium sources, and recycling options (e.g., sintering, hydrothermal, molten salt, electrochemical, and chemical methods).

**TABLE 1:** A summary of technical and economic parameters for grouped relithiation methods.

Method variations	CAMs	Reagent variations	Median *C_M_*, $∙kg^−^^1^	Median *E*, kJ∙g^−^^1^	Median *S_Group I_*, pts	IQR for *S_Group I_*	Ref.
	**Solid‐state reaction (SSR)**						
SSR‐1	LCO, NMC151570, NMC111, NMC631, NMC622, NMC532, NMC 830512, LMO	Li_2_CO_3_	37.0	560.0	88.6	5.17	[25, 26, 27, 62, 63, 64, 65, 66, 67, 68, 69, 70, 71, 72, 73, 74, 75, 76, 77, 78, 79, 80, 81, 82, 83, 84, 85, 86, 87, 88]
SSR‐2	NMC532, LCO, LMO, NMC622, NMC111	LiOH	73.6	429.3	87.0	6.15	[25, 63, 68, 84, 89, 90, 91, 92, 93, 94, 95, 96, 97, 98, 99, 100]
SSR‐3	NMC532, LCO, LMO, NMC622, NCM83	Biphenyl‐Li, amyloxyllithium, surface contaminants, CB and PVDF residuals, PTFE	12.5	582.1	90.0	11.51	[63, 72, 82, 101, 102, 103, 104, 105, 106]
	**Molten salt thermochemistry (MST)**						
MST‐1	NMС532, NMC622, NMC111	Inorganic lithium compounds: LiOH, LiNO_3_, Li_2_CO_3_, LiI	115.6	461.4	79.3	25.41	[107, 108, 109, 110, 111, 112, 113, 114, 115, 116, 117, 118, 119, 120, 121, 122, 123]
MST‐2	NMC111, NMC532, LCO	Organic: ionic liquid ([C_2_OHmim]), CH_3_COOLi, lithium salicylate, urea, betaine, LiCl	343.2	143.2	69.0	57.13	[28, 124, 125, 126, 127, 128, 129, 130, 131]
MST‐3	NMC532, NMC622, LCO, NCA	Inorganic lithium and non‐lithium compounds: KCl, KNO_3_, NaCl, KOH, NaOH, NaNO_3_, Na_2_SO_4_	470.5	907.6	52.6	34.13	[29, 30, 132, 133, 134, 135, 136]
	**Hydrothermal (Hydro)**						
Hydro‐1	NMC111, NMC151570, NMC553015, LCO, NMC532, LMO, LiNi_0.5_Mn_1.5_O_4_	LiOH‐based aqueous solution treatment	58.9	509.3	88.8	3.72	[31, 33, 137, 138, 139, 140, 141, 142, 143, 144, 145, 146, 147, 148]
Hydro‐2	LCO, NMC111, NMC532	LiOH‐based aqueous solution treatment with optional additives	58.9	425.9	88.2	2.94	[32, 75, 149, 150, 151, 152, 153, 154, 155]
Hydro‐3	NMC622, NMC532	Li compounds‐based solvothermal treatment	119.0	504.4	78.8	9.13	[156, 157, 158, 159]
	**Electrochemical (EC)**						
EC	LMO/NMC, LCO, NMC111, NMC413623, NMC622	Aqueous electrolytes: Li_2_SO_4_, LiNO_3_, LiOH Organic solutions: lithium bromide (LiBr) in acetonitrile (MeCN); battery electrolytes	147.7	143,0	71.9	16.0	[19, 20, 21, 160, 161, 162, 163, 164, 165, 166, 167, 168, 169, 170]
	**Chemical (Chem)**						
Chem	LCO, NMC622, LMO, NMC811	DTBQ, FL‐Li, Per‐Li, LiBr, DMSO, C_2_H_5_LiO, etc.	201.4	333.7	77.8	21.0	[22, 23, 24, 171, 172, 173, 174, 175, 176, 177, 178]

Three groups of publications can be identified for the SSR method of relithiation in according to the author's application of lithium carbonate, lithium hydroxide, and lithium organic compounds as a Li‐source. The utilization of lithium carbonate (SSR‐1) is a typical solution that has proven its efficiency for the synthesis of mixed oxide cathode materials. It is a rather cheap and common source of lithium compared to other lithium compounds. Therefore, the range of values for the cost of CAMs reagents for relithiation is quite narrow with a median value of about 37.0$∙kg^−1^. The core relithiation process is governed by a solid‐state reaction wherein mixed metal oxides undergo lithiation through thermal treatment with lithium carbonate, typically employing a controlled stoichiometric excess (5–10 mol%) to compensate for lithium loss at high temperatures. The differences between the publications are only in the pre‐ and post‐treatment approaches or sintering temperature regime. For example, Gao et al. [25] carried out sintering of LCO with Li_2_CO_3_ at temperatures of 500°C–900°C in steps of 100°C, Meng et al. [83] mechanochemically activated sample (500 rpm, 4 h) before applying different sintering temperatures (600°C, 700°C, 800°C, 900°C, 1000°C). Other authors added functional additives: Mn_2_O_3_ [86], MgO/Al_2_O_3_ [26], Co_3_O_4_ [64], MgO/TiO_2_ [74], Sm(NO_3_)_3_ [103]. MgO [67, 73], MgO/Al_2_O_3_/TiO_2_ [69]. The situation is similar for the group of publications using lithium hydroxide as a lithium source (SSR‐2). However, due to the higher cost of the latter compared to lithium carbonate, the SSR‐2 median value of the material costs is one and a half times higher (73.6$∙kg^−1^ vs 37.0$∙kg^−1^, respectively). Also, the SSR‐2 median value for energy consumption has a lower value of 429.3 vs 560.0 kJ·g^−1^, respectively, which is related to the lower sintering temperatures due to the lower melting point of lithium hydroxide (462°C) compared to lithium carbonate (732°C).

SSR‐3 achieves lower median relithiation costs (12.5$∙kg^−1^) due to in situ formed lithium‐containing surface contaminants from electrolyte decomposition [70, 82, 101, 102, 103, 179, 180] or self‐adsorbing lithium compounds (e.g., amyloxylithium [104], biphenyl‐Li [105]) that cover the surface of spent CAM particles with uniform lithium‐containing layers. During subsequent annealing, these compounds transform into lithium carbonate, facilitating uniform lithium access to damaged regions of the electrode material. For this reason, heat treatment uses temperature regimes similar to those used for SSR‐1. The core innovation of SSR‐3 lies in the utilization of pre‐existing lithium‐containing surface layers (either contaminants or deposited compounds). Since layers can be formed from surface contaminants, there is no need for additional sources of lithium, making SSR‐3 the most cost‐effective of all SSR methods.

When all SSR datasets (SSR‐1–3) are pooled, the distribution of *S_Group I_* scores remains tightly clustered, with a high overall median of ≈88–89 pts, a relatively narrow interquartile range of ∼6 pts, and an exceptionally low ΔLOO of ≈0.01–0.7. These robustness metrics suggest that the economic ranking of SSR methods is statistically robust: the central tendency is only weakly affected by the inclusion or exclusion of individual studies despite variations in lithium sources, additives, and sintering protocols. Therefore, the conclusions drawn in the comparative analysis of SSR‐1, SSR‐2, and SSR‐3 can be considered both internally consistent and reliable for use in broader techno‐economic assessments.

Three distinct approaches can be identified in MST‐based cathode relithiation, differentiated by their eutectic melt composition: inorganic lithium compounds, organic compounds, or hybrid systems combining lithium salts with inorganic additives. MST‐1 has similar features to the SSR method due to similar compounds application, such as LiOH, LiNO_3_, Li_2_CO_3_, LiI, resulting in similar temperature regimes application in some cases. However, MST‐1 differs fundamentally in employing a large stoichiometric excess of eutectic melt relative to CAM and performing the key relithiation step at much lower temperatures (typically 300–320°C). This results in a wider material cost of 35.3–5956.3$∙kg^−1^ and wider energy consumption of 228.8–611.4 kJ·g^−1^. For example, Ma et al. [116] used a more expensive eutectic LiI‐LiOH melt (45 mol.% LiOH) in a threefold excess, resulting in a material cost of $5956.3 at the laboratory stage for recycling 1 kg of CAM. Typical components for the formation of MST‐1 group eutectic mixtures are LiNO_3_/LiOH in different molar ratios [107, 117, 118] and LiOH/Li_2_CO_3_ [119, 120]. Across all publications, MST‐1 exhibits a median material cost of 115.6$·kg^−1^, a median energy consumption of 461.4 kJ·g^−1^, and a median *S_Group I_* score of 79.3 pts with a wide IQR of 25.41 pts, showing that despite its conceptual similarities to SSR‐1, its economic performance is far more variable. Nevertheless, MST‐1 remains the most cost‐competitive among MST subcategories.

The remaining pathways, MST‐2 and MST‐3, are significantly less economically attractive, with median *S_Group I_* scores of 69.0 pts and 52.6 pts, respectively. Their higher material costs (343.2$·kg^−^^1^ for MST‐2 and 470.5$·kg^−^^1^ for MST‐3) stem from either the use of expensive chemicals or the requirement of using large excesses of reagents. For example, 50 grams of LiOH‐KOH‐Li_2_CO_3_ eutectic mixture (molar ratio = 3: 7: 0.5), which was irretrievably lost was used to relithiate 1 gram of LCO [134], or the expensive ionic liquid [C_2_OHmim][NTf_2_] was used, 0.33 grams of which is irretrievably lost after relithiation of 1 gram of black powder [124]. Their energy consumption diverges strongly from MST‐1: MST‐2 has a median of only 143.2 kJ·g^−1^ due to the use of low‐melting organic eutectics (150–300°C), whereas MST‐3 reaches 907.6 kJ·g^−1^, the highest among all MST categories, reflecting the need for high‐temperature molten inorganic salts.

Clear differences in economic feasibility and robustness emerge when comparing MST variations with the corresponding SSR ones. Despite employing lithium salts similar to those in SSR‐1 and operating at comparable or lower temperatures, MST‐1 exhibits weaker economic performance. Its median Group I is lower, and both IQR and ΔLOO are substantially higher (Table S3). This indicates that favorable results rely on less statistically stable data than those in SSR‐1. Although MST‐2 benefits from lower energy consumption due to the use of low‐melting organic eutectics, this advantage is outweighed by higher and more variable reagent costs. Consequently, MST‐2 is less economically reliable than the combined SSR‐2 studies. MST‐3 performs the worst overall, being the most expensive, the least energy‐efficient, and the least statistically robust. Overall, MST‐based relithiation is more sensitive to specific experimental choices and less economically consistent. In contrast, all three SSR subgroups demonstrate more stable and reproducible cost–performance characteristics.

Three groups of publications can be identified relating to Hydro‐based CAM relithiation. Hydro‐1 uses aqueous LiOH solutions with varying concentrations and pre‐ and post‐treatment temperatures, and its economic performance is very similar to that of SSR‐1. Its median *S_Group I_* is 88.8 pts (vs 88.6 pts for SSR‐1), with a relatively narrow IQR of 3.72 pts and a tight 95% CI for the median (87.00–89.30), while ΔLOO remains low at 0.19. Although the median reagent cost for Hydro‐1 is slightly higher and the energy demand slightly lower than for SSR‐1, the overall economic efficiency of Hydro‐1 is comparable and statistically robust. Although Hydro‐2 introduces optional additives into the LiOH solution, thereby broadening the range of material costs, its central economic performance remains essentially unchanged. The median *S_Group I_* is 88.2 with an even narrower IQR of 2.94, a 95% CI of 86.96–88.77, and a ΔLOO of 0.14. This confirms that the cost–performance balance is not fundamentally altered by the addition of these additives. Represented by a smaller dataset (N = 6), Hydro‐3 exhibits a lower median *S_Group I_* of 78.8 points and a wider IQR of 9.13 points. With a ΔLOO of 4.56, it suggests reduced economic attractiveness and weaker robustness compared with Hydro‐1/2.

The modest impact of additives on Hydro‐2 scores can be rationalized by their targeted functional roles. Functional additives are introduced into lithium hydroxide solutions to optimize cost, energy efficiency, and material performance by precisely controlling redox chemistry, reaction kinetics, and product purity. Oxidizing agents such as H_2_O_2_ and (NH_4_)_2_S_2_O_8_ enhance relithiation efficiency by removing organic residues and facilitating the oxidation of Co^2+^/Ni^2+^ back to their trivalent states [32, 151]. Urea serves as a precipitation agent, enabling uniform doping of transition metals like Ni and Mn into the cathode structure [150]. To reduce energy consumption, organic additives such as ethanol and ethylene glycol lower the required reaction temperature to 90°C–100°C [32], while KOH forms low‐melting eutectic mixtures with LiOH, accelerating ion diffusion [149]. Additionally, retaining unwashed LiOH residues helps mitigate lithium loss during high‐temperature annealing [152]. Structural stability is further improved by incorporating Li_2_SO_4_, which suppresses LiOH decomposition and stabilizes the reaction environment [75]. These strategies collectively enhance the sustainability and efficiency of cathode regeneration processes.

Electrochemical relithiation encompasses diverse operational formats, including aqueous systems based on Li_2_SO_4_, LiNO_3_, LiOH, non‐aqueous electrolytes (e.g., MeCN/LiBr), static H‐cells, Li‐metal half‐cells, and galvanic spontaneous‐corrosion configurations. Relithiation can proceed under constant current, potentiostatic conditions, or via cyclic charge–discharge, and is applied either to dispersed CAM powders or to intact electrodes after surface cleaning. This methodological heterogeneity is reflected in the economic metrics. Although electrochemical methods exhibit a relatively wide cost distribution due to the diversity of electrolyte systems and cell configurations, the robustness indicators show that their central tendency remains reasonably stable: the interquartile range is moderate (IQR = 16 pts), and the leave‐one‐out sensitivity of the median (ΔLOO = 0.64) is substantially lower than that of **MST** approaches, indicating that the economic performance of EC is not strongly driven by single publications and remains comparatively reliable despite methodological variability.

The key strength of EC methods is their exceptionally low energy demand: the median consumption is only 143 kJ·g^−1^, nearly four times lower than SSR‐1 (560.0 kJ·g^−1^), because relithiation proceeds at room temperature [19, 21, 164, 165, 166, 170] and heating [160, 168] heating is only rarely required for the final crystallization. In some configurations, energy input becomes effectively negligible (e.g., spontaneous Al corrosion in LiBr/MeCN). However, despite this advantage, EC remains less cost‐effective overall (*S_Group I_* = 71.9 pts) due to the high price of lithium salts and supporting electrolytes, the need for specialized equipment, and long processing times, which collectively limit its scalability. These results are consistent with the intrinsic cost structure of EC methods. The use of high‐purity lithium salts, supporting electrolytes, and separators, as well as the use of partially non‐recoverable electrolyte volumes, naturally increases material costs. Meanwhile, variation in cell architecture, current density, and electrolyte formulation contributes to the wide range of reported values. Nevertheless, the moderate robustness metrics suggest that this variability primarily affects the extremes of the distribution, rather than shifting the central trend.

Chemical relithiation is based on mild reductive‐oxidative reactions to restore the lithium stoichiometry and structure of the active material. The main approach is to use redox mediators such as 5,10‐Dihydro‐5,10‐dimethylphenazine, 3,5‐di‐tert‐butyl‐o‐benzoquinone, or perylene, capable of transferring Li^+^ and electrons from metallic lithium to the cathode material [23, 24, 174]. One work realized an autooxidation cycle using LiBr in DMSO, where the solvent also plays the role of oxygen donor [22]. Some techniques include closed cycles of reagents with the possibility of their regeneration and reuse. From an economic perspective, chemical methods show a median material cost of 201.4$·kg^−1^ and a median energy consumption of 333.7 kJ·g^−1^, which is consistent with room‐temperature lithiation followed by brief annealing to restore crystallinity. The integrated criterion yields a median *S_Group I_* of 77.8 pts, with an IQR of 21 pts and a 95% CI of 63.52–82.31, indicating noticeable heterogeneity between implementations but a reasonably well‐defined central trend. The leave‐one‐out sensitivity of the median is 1.05, i.e., removing individual extreme studies only moderately perturbs the overall assessment. Combined with their simple equipment requirements (standard laboratory glassware, no electrochemical hardware) and short active processing times (< 1–2 h), these features make chemical methods economically more attractive than EC and MST‐2/3 at the laboratory scale.

**FIGURE 2:**
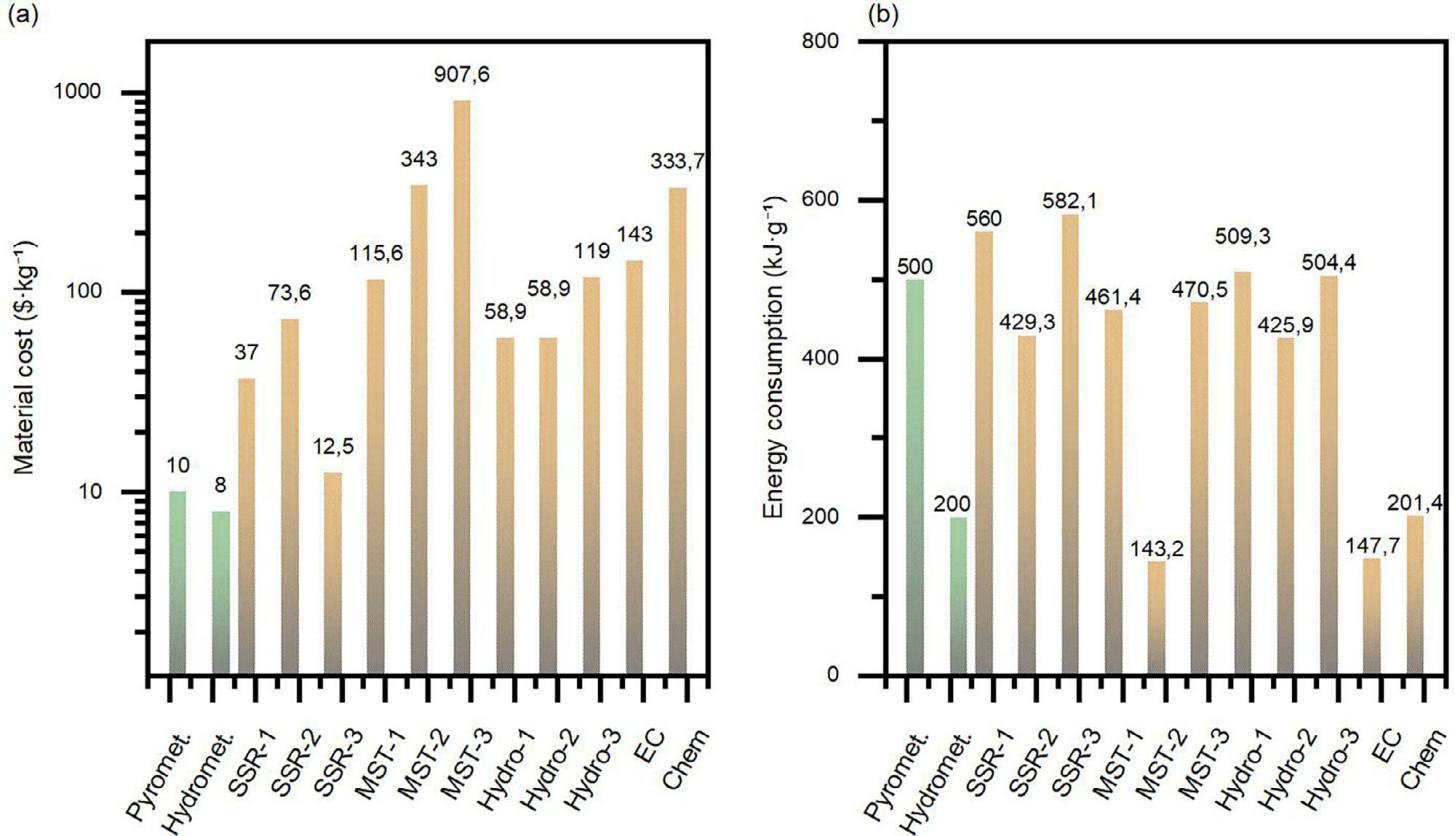
Comparative analysis of laboratory‐stage material costs (a) and energy consumption (b) for direct recycling vs. pyrometallurgy and hydrometallurgy. SSR—solid‐state relithiation; Hydro—hydrothermal relithiation; MST—molten‐salt thermochemistry; Chem—chemical relithiation; EC—electrochemical relithiation.

Figure 2 compares the median material costs and energy consumption associated with the various relithiation methods with the corresponding values obtained for large‐scale pyrometallurgical and hydrometallurgical recycling processes. Given the wide variation in material cost and energy consumption in the literature, we selected upper bound estimates for pyrometallurgy and hydrometallurgy from sources [23, 101, 172, 173, 180, 181, 182, 183, 184, 185, 186, 187] to provide a consistent and conservative benchmark for comparison. These maximum values are 500 kJ·g^−^^1^ and 10$∙kg^−1^ for pyrometallurgy and 200 kJ·g^−1^ and 8$∙kg^−1^ for hydrometallurgy. The costs mentioned for recycling materials are derived from lab‐scale studies, where reagent prices are based on small‐quantity laboratory‐grade purchases rather than bulk industrial pricing. This comparison reveals key differences between traditional recycling methods and new relithiation techniques, demonstrating the economic advantages of the new technologies.

When the recycling technology is scaled up from laboratory to industrial scale, the cost of 1 kg of cathode material can decrease significantly due to economies of scale, bulk purchasing of reagents, reduced overheads per unit throughput, and higher process efficiencies. Numerous techno‐economic analyses show that industrialization of cathode recycling can reduce material and processing costs by 5–20 times or more, depending on the method and the specific cost drivers such as energy supply, labor, capital equipment, and consumables [188, 189, 190, 191]; industrial recycling routings tend to achieve $0.9–4.1 kg^−1^ for direct recycling compared with substantially higher values for pyrometallurgical/hydrometallurgical routes at small scale [192].

For solid‐state and hydrothermal methods, industrial pricing of Li_2_CO_3_/LiOH and bulk utilities results in a material‐cost reduction factor of 8–15×, as demonstrated in TEA models for cathode manufacturing and recycling lines [193, 194, 195]. For molten‐salt routes, the achievable reduction is more modest because lithium salts remain the dominant cost driver even at bulk scale [196]. Electrochemical and chemical routes benefit least from scale, with only 2–4× reductions, due to consumable electrolytes and expensive mediators [192, 197]. However, scale‐up introduces distinct cost and energy bottlenecks. In all relithiation methods, the sintering steps required for recrystallization and activation of the cathode material remain the main factors affecting overall energy consumption, often accounting for 70%–90% of the total energy consumption of the process (Table S1 and Ref. [198]) due to high temperatures (≈700°C–900°C). Without replacing these stages with low‐temperature or high‐performance alternatives, total energy consumption remains comparable to that of pyrometallurgical methods. Another key factor in scaling is the limitations of throughput capacity and residence time in the reactor. For example, laboratory Hydro processes often use high liquid‐to‐solid ratios (1:20–1:100), which, when directly transferred to industrial volumes, imply reactors, pumping energy, and solvent reserves that are several orders of magnitude larger, making simple scale transfer economically less feasible without optimizing the use of reagents and recirculation flows. Similarly, MST processes using molten salts require careful thermal management to avoid energy losses and salt degradation in large volumes, as well as reactor volume optimization—a non‐trivial engineering challenge that affects both capital and operating costs. Even after optimization, achievable industrial S/L ratios remain 5–10× lower than SSR, increasing reactor volumes and thermal losses. SSR, conversely, scales straightforwardly with batch furnaces and belt calcination lines already used in cathode production. Meanwhile, EC and Chem relithiation methods, which nominally eliminate the need for high temperatures in more cases, entail other scaling costs associated with consumable electrolytes, mediators, and power electronics, which increase linearly with increasing throughput and can offset the benefits of reduced furnace energy consumption. These recurring consumable costs are a significant barrier to achieving the same scaling factors as SSR or Hydro methods.

In summary of this section, the diverse strategies developed for direct regeneration of spent cathode materials demonstrate substantial potential for sustainable, cost‐effective, and performance‐preserving recycling. Across the literature dataset, SSR and Hydro emerge as the most economically attractive and statistically reliable routes, with high median Group‐I scores (≈89 pts) and narrow IQRs (typically ∼3–6 pts for SSR‐1/2 and Hydro‐1/2), indicating a robust central tendency that is only weakly affected by reporting variability and outliers. In particular, SSR‐1 (Li_2_CO_3_) remains a practical benchmark due to low reagent costs and process maturity, while SSR‐3 achieves the lowest median material cost by exploiting surface‐derived lithium (electrolyte‐derived contaminants or self‐adsorbing lithium precursors) without fundamentally compromising overall economic performance. In contrast, molten salt thermochemistry demonstrates significantly weaker stability: although MST‐1 may be competitive in certain cases, the wide IQR (≈25 points) indicates high sensitivity to eutectic composition and excess reagent, and MST‐2/3 are further degraded by expensive organic substances or high salt consumption. Electrochemical and chemical methods retain clear advantages in terms of energy consumption (especially EC), but their lower median *S_Group I_* (≈72 points for EC; ≈78 points for Chem), combined with a wider spread (IQR ≈16–21 points) reflect the ongoing additional costs of consumable electrolytes, high‐purity salts, and expensive redox mediators. In general, the combination of median values with robustness descriptors (IQR, 95% CI of the median, and sensitivity ΔLOO) shows that SSR and Hydro not only provide high economic performance but also the most reproducible evidence bases in the literature, while MST, EC, and Chem are more dependent on specific experimental decisions and therefore require more careful process optimization and clearer reporting for confident prioritization of scaling.

### 3.2 General Comparative Cost Analysis of Cathode Relithiation Methods (Group I)

In addition to the detailed analysis of specific subcategories of relithiation strategies presented in Section 3.1, Section 3.2 provides a more generalized view of the different approaches. This broader view allows for a holistic comparison of all of the described relithiation methods, regardless of their categorization by lithium source or treatment conditions. By aggregating the data without dividing them into narrowly defined subgroups (e.g., SSR‐1, SSR‐2), we aim to identify general trends and performance measures that are representative of each method as a whole. This approach is particularly useful for identifying the comparative advantages and limitations of reticulation methods at the methodological level, which is important for selecting optimal pathways for scaling and industrial deployment.

**FIGURE 3:**
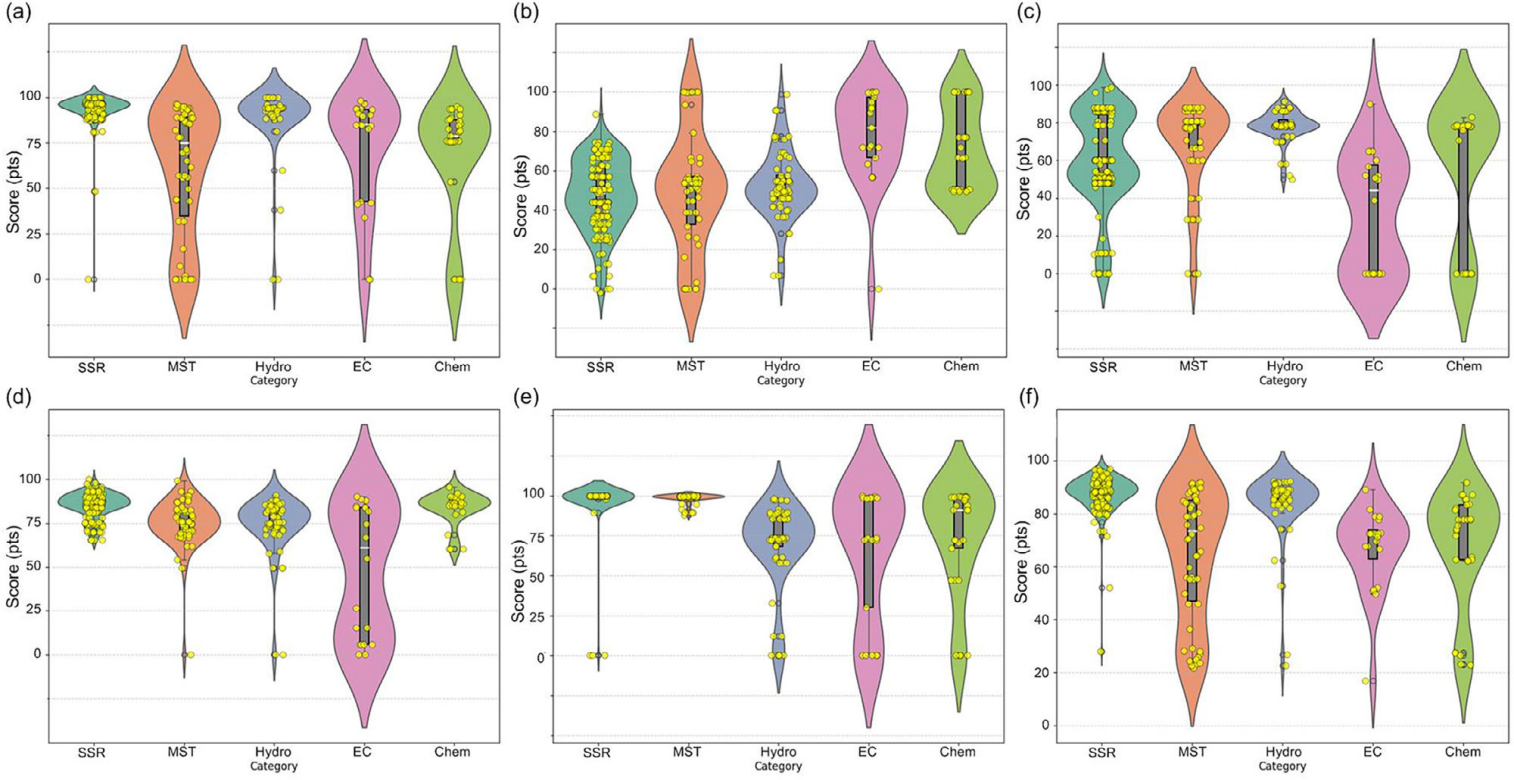
Violin‐boxplots of the score deviations of the four relithiation methods for the following criteria: (a) material costs; (b) energy consumption; (c) key equipment costs; (d) process duration; (e) space, and (f) total average scores. SSR—solid‐state relithiation; Hydro—hydrothermal relithiation; MST—molten‐salt thermochemistry; Chem— chemical relithiation; EC—electrochemical relithiation.

Across the five Group I criteria (Figure 3a–e; Table S4), the robustness analysis shows that the “best” method depends on which cost factor it focuses on and, crucially, whether the data reported in the literature is statistically stable. Estimates of material costs are highly reproducible for SSR and Hydro, with medians of 96.3 and 94.1, respectively, very narrow interquartile ranges (IQR) of 2.51 and 2.18, respectively, and a leave‐one‐out sensitivity close to zero. This indicates that their cost rankings are insensitive to outliers. In contrast, MST and, in particular, EC/Chem show significantly wider spreads (e.g., IQR EC 48.4 and IQR chemistry 32.83) and higher LOO sensitivity (up to 0.58–0.96). This means that their economic conclusions depend to a greater extent on a small, heterogeneous subset of publications. In terms of energy consumption, the SSR/Hydro/MST cluster falls within the lower median range (44.03–53.79), demonstrating moderate variability. This is consistent with the fact that the high‐temperature crystallization stage dominates energy consumption when present. The Chem and EC methods are the least energy‐intensive. The EC method received the highest score (89.4 points) due to its operation at room temperature, typically without a high‐temperature annealing step. The Chem method shows a moderate average score of 66.5 points, reflecting the variability in energy utilization data across publications. The wide interquartile ranges (IQRs) and elevated ΔLOO values for EC and Chem (Table S4) suggest that claims of low energy consumption depend on cell configuration, electrolyte selection, and assumptions about subsequent heat treatment. In terms of equipment, Hydro and MST show relatively stable medians (78.7–80.7) with moderate variation, while EC and Chem show the greatest uncertainty, as some publications receive zero scores for design when the required equipment exceeds the upper limit of the accepted range of scores (i.e., extremely high equipment costs), making their medians less representative and ΔLOO higher. Finally, process duration and space requirements are the least reliable differentiators for EC (very large IQR and highest ΔLOO, Table S4), while SSR remains consistently fast (median 86.17; narrow IQR 7.63; low ΔLOO 0.17) and space‐efficient (median 99.84; IQR 0.09; ΔLOO 0.00), making it the most stable option for throughput‐oriented scaling. Overall, the combined median–IQR–CI–ΔLOO profile demonstrates that SSR and Hydro provide the most reliable evidence base for decision‐making, while MST/EC/ Chem require more rigorous standardization of reagent use, system definition, and reporting before their apparent advantages can be confidently extrapolated to industrial process design.

### 3.3 Competitiveness Comparison of Solid‐State and Hydrothermal Regeneration with Conventional Recycling Pathways

To facilitate consistent and meaningful comparison of economic indicators, we have converted all published estimates of costs and profits for SSR technology to a common basis in dollars per kilogram of CAM. For this purpose, we assumed a cathode active material content of approximately 31% of the total cell mass, which is consistent with established estimates of battery composition in modern lithium‐ion cells [199]. This normalization allows for a direct comparison between SSR cost estimates from various sources and the selected cost indicators for pyrometallurgical (∼10$·kg^−1^ CAM) and hydrometallurgical (∼8$·kg^−1^ CAM) processing used in this study. The following section compiles and summarizes SSR cost data from recent technical and economic studies, expressed in US dollars per kg of CAM, and compares it with traditional processing benchmarks. Where necessary, costs originally stated on a different basis have been pragmatically recalculated using the above‐mentioned mass fraction of CAM to ensure comparability.

**1. Hydrothermal recycling cost comparison**. Numerous technical and economic analyses of hydrothermal methods show a slightly lower cost range than in the case of pyrometallurgy and hydrometallurgy. For example: (i) in direct low‐temperature hydrothermal relithiation at a throughput of 10 000 t·yr^−1^ where 4 m LiOH aqueous solution with addition of a 1%–3% (v/v) of green additive (ethanol, hydrogen peroxide or ethylene glycol) was used, the total recycling cost was modeled at approximately 6.8$·kg^−1^ CAM, slightly below both pyrometallurgical and hydrometallurgical costs [32]; (ii) in H_2_O_2_ containing aqueous direct regeneration for NMC532 cathodes, EverBatt‐based assessment showed higher revenue (≈ 6.9$·kg^−1^) than conventional methods [149]; (iii) in a comparative EverBatt 2020 evaluation across ethanol solution containing lithium nitrate solvothermal regeneration exhibited costs (∼6.9$·kg^−1^) that were still below many classical benchmarks when accounting for product value [156]; (iv) A urea thermal melting direct regeneration method reported the lowest cost among direct approaches (≈ 6.32$·kg^−1^) with a high profit of ∼2.03$·kg^−1^, again below the cost levels of pyrometallurgy and hydrometallurgy [157].

Taken together, these analyses suggest that hydrothermal regeneration methods can offer cost advantages over conventional pyrometallurgical (≈ 10$·kg^−1^) and hydrometallurgical (≈ 8$·kg^−1^) recycling under modeled industrial conditions. In the available literature, hydrothermal variants typically fall within a cost range below 7$·kg^−1^ at functional unit scales relevant to industry, consistently below the traditional metallurgy benchmarks.

However, when making such comparisons, it is necessary to take into account the scalability and current maturity of the industry: (i) many estimates of hydrothermal costs are based on EverBatt models or laboratory parameters adjusted for industrial performance, rather than data measured at industrial plants [32, 156, 157]. (ii) hydrothermal processes often require the use of specialized equipment (e.g., pressure vessels, controlled water environments), which can entail capital expenditures (CAPEX) and operating expenses (OPEX) that are not fully reflected in the model forecasts. Therefore, although lower specific operating costs are reported in the literature, the total cost of scaling up hydrothermal recycling to several kilotons will depend on reactor economics, feedstock logistics, and integration with existing extraction and purification infrastructure.

Thus, the available technical and economic data suggest that hydrothermal regeneration is a cost‐effective alternative to hydrometallurgical and pyrometallurgical recycling, which cost ∼8$·kg^−1^ and ∼10$·kg^−1^, respectively. A number of studies have noted that the costs of hydrothermal regeneration are generally lower. This is also consistent with the results of a laboratory assessment of the material costs (Figure 2a) and the assumption that costs can be reduced by 5–10 times when moving from a laboratory to an industrial scale.

**2. Solid‐state recycling cost comparison**. For direct recycling via solid‐state methods, a growing body of techno‐economic analyses indicates that SSR can be cost‐competitive to conventional hydrometallurgical (∼8$·kg^−1^ CAM) and pyrometallurgical (∼10$·kg^−1^ CAM) benchmarks. Among recent SSR‐type estimates, a number of studies report regenerated CAM costs below or close to the hydrometallurgical benchmark. In particular, 6.71$·kg^−1^ and 8.06$·kg^−1^ were reported for LCO and black mass/NCM regeneration scenarios [89, 91], respectively. A SSR method using CaO at a cost of 6.5$·kg^−1^ was also presented, indicating that even when additional reagents are required for impurity removal or process stabilization, SSR can remain relatively low‐cost one [78]. Other SSR variants are within the pyrometallurgical benchmark or slightly above it. For instance, an EverBatt‐based closed‐loop evaluation reported 9.54$·kg^−1^ for homogenizing phase‐component regeneration of S‐NCM811 [200]. Meanwhile, several examples of direct SSR modernization show that costs range from ∼$11 to $13 per kg, meaning that cost competitiveness may depend on specific additives and the volume of accounting adopted for plant operation and pre‐treatment [76, 106]. Altogether, the best available data on SSR costs indicate an approximate range of 6.5–13.2$·kg^−1^ based on recent direct regeneration studies. The practical limit for SSR scaling is determined not only by the presence of a solid‐state stage, but also by whether the SSR process flow can support low reagent consumption, high product yield, and limited auxiliary processing while maintaining the structural value of the cathode.

### 3.4 Review of Publications from an Electrochemical Performance Perspective

While Section 3.1 considered regeneration strategies from a cost‐effective perspective, focusing on reagent cost, energy consumption, and integral process efficiency, this section focuses on the functional recovery of cathode materials, which ultimately determines their potential for practical reuse. Indeed, a regeneration method that is cost‐effective but does not restore key electrochemical properties of the active material may not be suitable for closed‐loop recycling in real‐world applications. And at the same time, if the regeneration method restores the electrochemical performance at a high cost, it is also unsuitable for practical applications.

**TABLE 2:** Comparative summary of dominant degradation mechanisms in layered oxide cathodes (LCO, NMC532/622, and NMC811).

Mechanism / Aspect	NMC811	NMC532 / NMC622	LiCoO_2_
Surface reactivity / electrolyte decomposition / gas evolution	Lower onset potential for gas evolution (≈4.0 V), pronounced release of CO_2_/CO/O_2_ during charging; stronger interfacial parasitic reactions [208, 211, 212].	More stable up to ≈4.3–4.4 V; less pronounced gas release and interfacial reactivity [208, 211, 212].	Stable under moderate voltages, but above 4.6 V carbonate electrolytes degrade with CO_2_/CO evolution and dynamic CEI formation [86, 213, 214, 215].
Surface reconstruction (rock‐salt / spinel formation)	Rapid transformation of surface layers into rock‐salt/spinel phases; thick and poorly conducting interphases [211, 216, 217].	Reconstruction occurs more slowly and with smaller penetration depth [211, 216, 217].	Overcharge drives O3→H1‐3→O1 transitions and CoO/Co_3_O_4_ phases; thin rock‐salt /spinel layers may form, facet‐dependent rock‐salt reconstruction reported [218, 219, 220].
Ni^2^^+^ migration / cation mixing (Li/Ni disorder)	Significant Li/Ni intermixing; lower I(003)/I(104) XRD ratio; Ni^2^^+^ migration into Li layers even at moderate delithiation [211, 216, 217, 221, 222].	Higher I(003)/I(104) ratio; less cation disorder and improved structural ordering [211, 217, 221].	Li/Co mixing limited; Co migration/dissolution significant only at deep overcharge (> 174% SOC); rock‐salt layers delay Co migration [218, 219].
Crack formation / mechanical degradation	Severe intra‐ and intergranular cracking due to ≈5% unit cell volume change upon cycling; accelerated impedance rises [211, 216, 222].	Lower lattice strain (≈1%–2% volume change), reduced crack propagation, slower impedance growth [211, 216, 222].	Microcracks appear mainly under severe overcharge or prolonged cycling; rock‐salt‐modified LCO shows suppressed cracking [86, 218, 219].
Oxygen release / lattice oxygen instability	Lower onset potential for oxygen release (≈4.0–4.2 V); stronger exothermic reactions upon heating [211, 216, 222].	Oxygen release initiates at higher potentials (> 4.3 V); oxygen lattice more stable [211, 216, 222].	Oxygen instability above 4.6 V; oxygen radicals trigger electrolyte oxidation; rocket‐salt layers capture lattice O and suppress release [215, 219, 220].
Transition metal dissolution	More pronounced dissolution of Ni/Co/Mn; stronger cross‐talk leading to SEI destabilization on the anode [208, 216, 222].	Less transition metal dissolution; slower degradation of anode SEI [208, 216, 222].	Significant Co dissolution at deep overcharge; rocket‐salt ‐engineering reduces Co loss and CEI thickness [215, 219, 220].

#### 3.4.1 Mixed‐Oxide Cathode Degradation Mechanisms

The electrochemical characteristics of regenerated layered oxide cathodes are inherently determined by degradation mechanisms that occur during their lifespan. These mechanisms are partly common to all layered oxides, and they determine to a critical extent the degree to which regeneration strategies can restore the original functionality. The main degradation pathways include structural phase transitions, oxygen loss in the lattice, dissolution of transition metals, surface reconstruction into electrochemically inactive phases, interphase film growth, and particle destruction [201, 202, 203, 204, 205]. Below, we briefly describe the main degradation modes for typical cathode families, serving as a conceptual bridge to the analysis of electrochemical characteristics in section 3.4.2. A comparative summary of the dominant degradation mechanisms is presented in Table 2.

**FIGURE 4:**
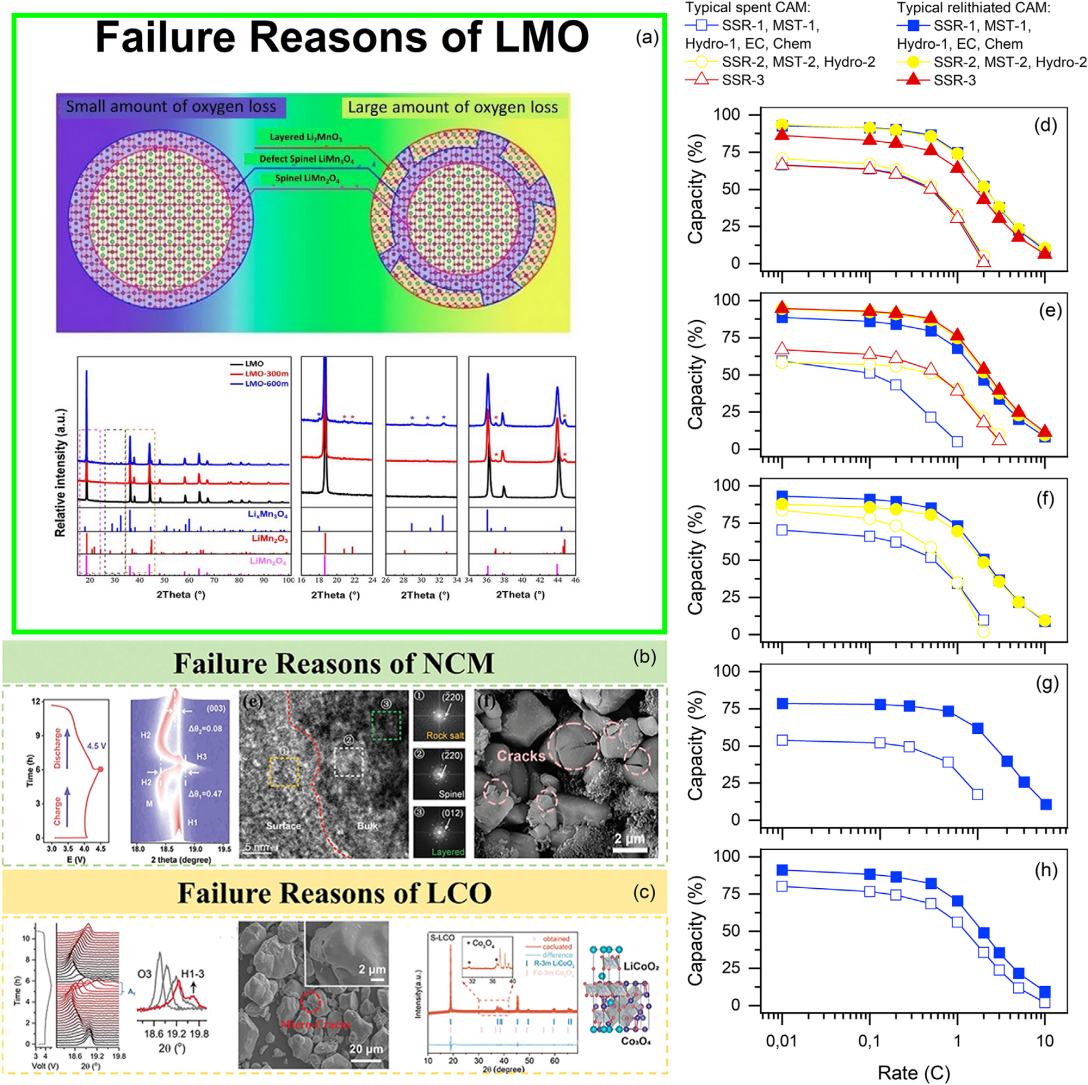
Failure mechanisms of LMO (a): *(Top)* Schematic of Mn migration on the LiMn_2_O_4_ surface, leading to the formation of defect‐spinel LiMn_3_O_4_ (I4_1_/amd) and layered Li_2_MnO_3_ (C2/m) structures. *(Bottom)* XRD patterns of LiMn_2_O_4_ heated at 600°C for 300 and 600 min under argon flow, where blue and red asterisks denote reflections corresponding to defect‐spinel LiMn_3_O_4_ (I4_1_/amd) and layered Li_2_MnO_3_ (C2/m) [207], respectively. Reproduced with permission [207]. Copyright 2025 American Chemical Society. Failure mechanisms of NMCs (b): *(Left to right)* In situ XRD patterns (17.8–19.7°) with corresponding voltage curves for NCM during charge/discharge (3.0–4.5 V) [201]. HRTEM images of S‐NCM with FFT patterns (below) corresponding to the dashed‐box regions [201]. SEM images of NCM [201]. Reproduced from [201] under CC BY 4.0. Failure mechanisms of LCO (c): *(Left to right)* In situ XRD patterns and galvanostatic charge/discharge curves of LCO cathodes during initial cycling [201]. XRD Rietveld refinement results for S‐LCO with its crystal structure shown on the right [201]. Reproduced from [201] under CC BY 4.0. Simulated rate performance based on Table 3 using the equation from the publication by Tian et al. [56], comparing typical cathode materials before and after relithiation via five relithiation methods: SSR (d), MST (e), Hydro (f), EC (g), and Chem (h). SSR—solid‐state relithiation; Hydro—hydrothermal relithiation; MST—molten‐salt thermochemistry; Chem—chemical relithiation; EC—electrochemical relithiation.

**LMO degradation mechanism**. Under long‐term cycling, the surface of spinel‐type LMO materials can undergo local structural distortions, initiating the transformation of the spinel framework into a layer‐like structure [202, 205, 206] (Figure 4a). This phenomenon is attributed to the migration of Mn ions through the tetrahedral sites, which is facilitated by the gradual oxygen loss. The resulting Mn^3^^+^ disproportionation into Mn^2^^+^ and Mn^4^^+^ alters the local coordination environment and triggers phase separation at the surface and near‐surface regions. Eventually, these distortions propagate into the bulk, disrupting the structural integrity. In addition, the formation of intermediate phases such as LiMn_3_O_4_ and Li_2_MnO_3_ contributes to the loss of active lithium and decreased reversibility. These transformations lead to an increase in charge transfer resistance, suppression of lithium mobility, and a capacity decrease.

**NMC degradation mechanism**. In NMC‐type layered oxides, long‐term cycling induces irreversible phase transitions (H1–M–H2–H3–H2–H1) with the H3 phase notably causing abrupt c‐axis contraction [204, 208] (Figure 4b). In combination with cation mixing (Li^+^/Ni^2^^+^ exchange) due to similar ionic radii, this leads to the formation of spinel‐ and rock‐salt‐like surface phases. These changes disrupt the layered structure, limit lithium diffusion, and increase internal stresses. The penetration of the rock salt phase into the bulk leads to the formation of microcracks and distortion of the crystal lattice, which further accelerates the capacity decrease and kinetic deterioration.

**LCO degradation mechanism**. In LiCoO_2_, structural degradation is dominated by slab gliding and interlayer repulsion during lithium extraction [203, 204, 208] (Figure 4c). Above ∼4.5–4.6 V, the O3 → H1–3 phase transition induces repeated c‐axis expansion/contraction, generating mechanical stress and microcracks. Simultaneously, lattice oxygen oxidation produces reactive oxygen species that drive electrolyte decomposition and facilitate irreversible oxygen loss. These processes eventually promote surface transformation into Co_3_O_4_ spinel and rock‐salt phases. In addition, cobalt dissolution into the electrolyte and subsequent deposition on the anode further destabilize the SEI and aggravate capacity decay.

**Comparative perspective**. While these degradation modes differ in their onset potential and severity, they are mechanistically interconnected and largely shared across layered chemistries. However, systems with a high nickel content and, in particular, NMC811, require separate consideration. Compared to compositions with average nickel content, such as NMC532 and NMC622, NMC811 exhibits the same basic degradation pathways (surface reconstruction, cation mixing, oxygen evolution, cracking, and dissolution of transition metals), but these processes occur earlier, proceed more aggressively, and often reinforce each other. Due to the increased Ni content, deeper redox activity of oxygen and weaker metal‐oxygen bonding, cathodes with high Ni content are prone to faster oxygen loss, severe cation disorder (especially Ni^2^^+^ migration to Li sites), and increased microcracking under cycling. Indeed, DFT studies show spontaneous O_2_ release through the formation of surface oxygen holes in delithated Ni oxides [209] and non‐stoichiometry kinetics facilitate easier mixing of Li/ Ni in phases with high nickel content [210]. For this reason, the failure mechanisms of NMC811 can be described as “amplified” or “accelerated” versions of those in lower‐Ni analogues, resulting in faster impedance rise, poorer cycling stability and reduced thermal safety. Table 2 summarizes the main degradation pathways in LCO, NMC532/622 and NMC811, focusing on both their common features and the amplification of nickel‐dependent mechanisms in high‐nickel systems. Based on this table, Section 3.4.2 discusses how different regeneration strategies affect these degradation modes and evaluates their effectiveness in restoring electrochemical performance. Section 3.4.3 then critically assesses whether methods optimized for LCO and NMC with medium Ni content can be transferred to NMC811.

#### 3.4.2 Comparative Electrochemical Performance Analysis of Regeneration Methods

To estimate the electrochemical performance, we analyzed published studies reporting the electrochemical characteristics of cathode materials regenerated by various methods. The results are summarized in Table 3, which presents the ranges and median values of key parameters: state of health (SoH), rate capability, cycling stability, as well as the integrated performance criteria (SGroup II). This criterion reflects how well a particular method, in general or in particular, recovers the electrochemical characteristic of the cathode material compared to others and serves as a composite indicator for further development and balancing of a particular publication or method. Table 3 gives an insight into what electrochemical characteristics a cathode material regenerated by a certain method would have, based on results already published in the scientific literature.

To quantify the degree of rate capability recovery, we used the kinetic model proposed by Tian et al. (2019) [55, 56, 223], which characterizes rate performance by two parameters: *τ* (characteristic time) reflecting kinetic limitations and *n* (exponent) indicating the nature of the rate‐limiting mechanism. According to this model, a value of *n* → 0.5 corresponds to diffusion‐limited behavior while values approaching *n* → 1 indicate resistance‐limited behavior caused by poor electronic or ionic conductivity, interfacial degradation, or structural damage. After evaluating *n* and *τ* for all publications, we obtained their median values for each of the relithiation methods. These median values, combined with the median SoH values (Table 3) based on the equation from [55, 56, 223], allowed us to simulate what the most typical c‐rate curve of a cathode material would look like before and after regeneration (Figure 4d–h). Despite the variety of cathode compositions, including LMO, NMCs, LCO, and the different dominant degradation mechanisms (Figure 4a–c), we observe a remarkable consistency in the *n* values before regeneration, with most cases ranging from 0.90 to 1.03. This coincidence suggests that degradation in all cases studied is mainly driven by resistive constraints regardless of material type or degradation history.

**TABLE 3:** A summary of electrochemical performance for grouped relithiation methods.

Method variations	Dominant degradation mechanisms	SoH, % range and median		*τ*, h range and median		Cycling performance	*S_Group II_*, pts Median	Ref.
		Spent	Regenerated	Spent	Regenerated			
			**Solid‐state relithiation (SSR)**					
SSR‐1	2 > 4 > 3 > 5 > 1 > 6	5.7%–100%, Median 62.8%	41.1%–100%, Median 91.5%	2.9∙10^−3^ – 2.8 h Median 0.48 h	3.2∙10^−3^–1.6 h Median 0.05 h	Cycles: 30–500, mоde 100. Currents: 0.1C–10C, mоde 1C. Capacity retention: 35.6%–100%, median 89.5%	5.6–86.6 pts Median 61.5 pts	[25, 26, 27, 62, 63, 64, 65, 66, 67, 68, 69, 70, 71, 72, 73, 74, 75, 76, 77, 78, 79, 80, 81, 82, 83, 84, 85, 86, 87, 88]
SSR‐2	2 = 3 > 4 > 5 > 6 > 1	12.7%–100.0%, Median 65.7%	71.4%–100%, Median 94.5%	0.085–1.1 h Median 0.42 h	1.0∙10^−3^–0.34 h Median 0.026 h	Cycles: 30–300, mоde 100. Currents: 0.15C–1C, mоde 1C. Capacity retention: 61.3–94.5%, median 79.0%	30.6–95.3 pts Median 60.0 pts	[25, 63, 68, 84, 89, 90, 91, 92, 93, 94, 95, 96, 97, 98, 99, 100]
SSR‐3	2 > 3 = 4 > 6 > 1 = 5	3.35%–92.7%, Median 62.0%	52.5%–100%, Median 88.5%	0.05–4.7 h Median 0.64 h	1.6∙10^−3^–0.37 h Median 0.062 h	Cycles: 50–500, mоde 200. Currents: 0.33C–5C, mоde 1C. Capacity retention: 38.8–93.5%, median 80.5%	4.5–99.0 pts Median 44.0 pts	[63, 72, 82, 101, 102, 103, 104, 105, 106]
			**Molten‐salt thermochemistry (MST)**					
MST‐1	2 = 3 > 5 > 4 > 6 > 1	18.8%–82.8%, Median 62.2%	68.0%–100.0%, Median 90.3%	0.016–4.3 h Median 0.43 h	1.0∙10^−3^–0.256 h Median 0.029 h	Cycles: 100–500, mоde 200. Currents: 0.5C–1C, mоde 1C. Capacity retention: 28.8–92.0%, median 83.1%.	13.7–80.9 pts Median 55.0 pts	[107, 108, 109, 110, 111, 112, 113, 114, 115, 116, 117, 118, 119, 120, 121, 122, 123]
MST‐2	2 = 3 > 5 > 4 > 1 > 6	4.0%–91.0%, Median 62.4%	78.5%–100.0%, Median 96.0%	0.210–3.8 h Median 0.3 h	2.5∙10^−3^–0.112 h Median 0.037 h	Cycles: 100–200, mоde 100. Currents: 0.1C–1C, mоde 0.5C. Capacity retention: 31–95.6%, median 84.3%.	40.2–86.2 pts Median 69.5 pts	[28, 124, 125, 126, 127, 128, 129, 130, 131]
MST‐3	2 = 3 = 5 > 4 > 6 > 1	32.0%–98.0%, Median 51.0%	90.3%–100.0%, Median 98.0%	0.019–0.580 h Median 0.261 h	6.5∙10^−3^–0.068 h Median 0.010 h	Cycles: 100–300, mоde 100. Currents: 0.2C–1C, mоde 1C. Capacity retention: 82.1–95.5%, median 88.0%.	67.0–78.1 pts Median 72.1 pts	[29, 30, 132, 133, 134, 135, 136]
			**Hydrothermal relithiation (Hydro)**					
Hydro‐1	2 = 5 > 3 > 6 > 4 > 1	6.3%–95.0%, Median 69.2%	70.5%–100.0%, Median 93.1%	0.018–4830 h Median 0.223 h	1.9∙10^−3^–0.134 h Median 0.032 h	Cycles: 50–300, mоde 100. Currents: 0.1C–1C, mоde 1C. Capacity retention: 40.5–98.7%, median 89.5%.	8.6–95.6 pts Median 48.2 pts	[31, 33, 137, 138, 139, 140, 141, 142, 143, 144, 145, 146, 147, 148]
Hydro‐2	2 = 3 > 5 > 6 > 4 > 1	46.6%–93.8%, Median 83.0%	81.4%–100.0%, Median 87.7%	0.085–2.2 h Median 0.49 h	0.012–0.043 h Median 0.022 h	Cycles: 50–200, mоde 100. Currents: 0.1C–1C, mоde 1C. Capacity retention: 26.9–95.4%, median 88.5%.	11.3–78.6 pts Median 30.5 pts	[32, 75, 149, 150, 151, 152, 153, 154, 155]
Hydro‐3	2 = 3 = 6 > 4 = 5 > 1	20.0%–55.5%, Mean 54%	77.1%–96.8%, Median 92.3%	1 – 2.8 h (Insufficient data)	0.045–0.080 (Insufficient data)	Cycles: 100–200, mоde 100. Currents: 0.2C–1C, mоde 0.2C. Capacity retention: 61.2–79.3%, median 74.0%.	74.3–86.2 pts Mean 80.0 pts (Insufficient data)	[156, 157, 158, 159]
			**Electrochemical relithiation (EC)**					
EC	2 > 3 > 1 = 6 > 5 > 4	10.0%–100.0%, Median 67.9%	40.9%–100.0%, Median 93.7%	0.040–0.930 h Median 0.59 h	0.012–0.160 h Median 0.100 h	Cycles: 50–600, mоde 100. Currents: 0.1C–5C, mоde 0.2C. Capacity retention: 40.0–100.0%, median 92.3%.	3.4–72.2 pts Median 41.0 pts	[19, 20, 21, 160, 161, 162, 163, 164, 165, 166, 167, 168, 169, 170]
			**Chemical relithiation (Chem)**					
Chem	2 = 3 > 4 = 5 > 1 > 6	56.0%–100.0%, Median 80.7%	77.0%–100.0%, Median 91.3%	0.036–1.75 h Median 0.207 h	8.4∙10^−3^–0.048 h Median 0.021 h	Cycles: 50–150, mоde 100. Currents: 0.5C–3C, mоde 1C. Capacity retention: 35.7 – 94.5%, median 82.4%.	9.0–71.0 pts Median 57.0 pts	[22, 23, 24, 171, 172, 173, 174, 175, 176, 177, 178]

Although the parameter *n* provides an indication of the dominant mechanism limiting the rate, its values remain clustered around unity for different cathode compositions, indicating a predominantly resistive origin of degradation. However, *n* alone does not reflect differences in the internal kinetics of charge transfer. This role is played by the characteristic time constant *τ*, which directly reflects the effective timescale of charge/discharge processes and integrates the contributions of electrical resistance, ion diffusion, and interfacial kinetics. Thus, studying *τ* allows us to distinguish between materials and regeneration strategies that appear similar in terms of *n* but differ significantly in their transport dynamics. This decrease in *τ* is interpreted as an indicator of faster charge transport kinetics in accordance with the original analysis by Tian et al. [55, 56, 223]. Since *τ* reflects the timescale over which capacity begins to decay under increasing rate (i.e., how rapidly the system becomes rate‐limited), its reduction suggests that the regenerated materials exhibit improved charge‐transfer efficiency. This improvement is likely due to the recovery of both bulk conductivity through structural restoration of the active material and interfacial conductivity through removal of passivating layers (e.g., CEI) and restoration of conductive pathways between particles. Moreover, the greater the difference between *τ* of the regenerated and spent cathode material, the more preferable is the relithiation method, as it demonstrated greater efficiency in recovering rate capability. In our work, the quantitative value is expressed by the *S_c‐rate_* criteria, which is a component of the *S_Group II_* integral criteria. According to Table 3, the efficiency in recovery rate capability qualitatively decreases in the series: Hydro‐2> SSR‐2> MST‐1> SSR‐3> Chem > SSR‐1> MST‐2> Hydro‐1> EC despite the fact that the capacity retention values can be close to each other. Notably, although MST‐3 exhibits the highest apparent performance in this metric, it is excluded from the ranking due to the insufficient statistical representativeness of the available dataset.

The ranked order of dominant degradation mechanisms in Table 3 shows consistent agreement with the electrochemical descriptors from Tian's model [55, 56, 223]. It is noteworthy that surface reconstruction (No. 2) leads the ranking for almost all relithiation methods; this predominance correlates with the observation that the *n* values are clustered close to 1, indicating that resistive limitations (rather than diffusion) prevail in degraded cathodes. The formation of a thick or disordered surface layer of rock salt/spinel is widely considered to be a detrimental consequence of structural collapse: it introduces electronic and ionic resistance, blocks Li^+^ pathways, and impairs capacity recovery, and accordingly, many regeneration protocols are explicitly aimed at its removal or suppression. However, it should not be assumed that all rock salt layers are universally harmful; their electrochemical impact depends on parameters such as thickness, crystallinity, defect content, and coherence with the underlying lattice [224, 225, 226]. In carefully designed systems, ultrathin coherent layers can contribute to surface stabilization, but only if they themselves do not become a rate‐limiting factor.

The differences in the degree of *τ* recovery between methods reflect how well each technique handles the second and third mechanisms. SSR (notably SSR‐1/2), which highly values cation disorder (No. 3) and crack formation (No. 4), typically produces a more pronounced reduction in *τ*, consistent with the restoration of bulk conductivity and the reduction of diffusion pathways. In contrast, MST and Hydro tend to prioritize oxygen release/oxygen loss at the surface (No. 5) immediately after No. 2–3, while Hydro‐1 uniquely places No. 5 among the top priorities along with No. 2 (2 = 5> 3> 6> 4> 1), reflecting a stronger emphasis on suppressing oxygen‐related degradation pathways. Consequently, Hydro‐2 and MST‐1 demonstrate a reduction in *τ* comparable to SSR‐2, while EC demonstrates the smallest average improvement (*τ* remains relatively high after treatment). Electrochemical methods sometimes prioritize the dissolution of transition metals (No. 6), promoting cycling stability but with limited impact on *τ* if structural and resistive defects remain unresolved. This coupling between mechanistic rankings and the Tian‐model parameters underscores a nuanced picture: while *n* functions as a fingerprint of the limiting mechanism (resistive vs diffusive), *τ* quantitatively measures how effectively regeneration mitigates specific degradation channels. Integrating mechanistic rankings (Table 3; Figures S2 and S3) with *n, τ*, and SoH therefore establishes a more robust framework for comparing relithiation strategies.

Beyond rate capability, an equally important indicator of regeneration quality is SoH and cycling stability of the regenerated cathode materials. These parameters determine the restoration degree of the original material functionality and its capacity retention under long‐term cycling that are expressed as relithiation efficiency in form of an integral criteria *S_Group II_*. A method‐by‐method comparison of *S_Group II_* scores reveals the following ranking of relithiation effectiveness:

– **Hydro‐3** (80.0 pts): This method shows the best integrated electrochemical performance among all analyzed approaches. It achieves high SoH (92.3%), excellent high‐rate capability changes but its cyclic stability is poor enough (74.0%). However, the amount of data to evaluate some parameters was insufficient. Therefore, solvothermal treatment is worth paying attention to as one of the promising regeneration methods, which requires additional research and a set of statistical data for better comparison with other methods.
– **MST‐3** (72.1 pts): Following Hydro‐3, MST‐3 also shows excellent SoH recovery (98.0%) and enough high‐capacity retention (88.0%) as well as one of the best rate changes among all methods to be second in the ranking. These results indicate that MST‐3 effectively recovers both lithium content and fast charge–discharge rate.
– **MST‐2** (69.5 pts): This method achieves high SoH (96.0%), high cycling stability (84.3%), and above‐average rate, reflecting the advantages of using mixed lithium sources in molten organic media.
– **SSR‐1** (61.5 pts): The most efficient among SSR methods. This method results in high SoH (91.5%) and good cycling stability (89.5%), but relatively weaker rate performance, which lowers its total effectiveness.
– **SSR‐2** (60.0 pts): SSR‐2 provides SoH of 94.2%, cycling retention of 79.0% and well recovered rate capabilities.
– **Chem** (57.0 pts): Despite a relatively simple process, Chem exhibits high SoH recovery (91.3%), sufficient cycling stability (82.4%), and fairly low recovery of rate capabilities.
– **MST‐1** (55.0 pts): MST‐1 exhibits moderate SoH (90.3%), low cyclic stability (83.1%), and average speed of operation, yielding an average integral score. While not as high performing as MST‐2 and MST‐3, it still provides functional recovery suitable for certain reuse scenarios.
– Other relithiation methods have *S_Group II_* values of about 50 or lower. All of these methods show one high, one moderate, and one relatively low value for the three individual performance parameters. As a result, their integrated performance score remains relatively low when compared to the previously discussed methods. These directions require additional efforts to improve the electrochemical performance of the relithiation material.

#### 3.4.3 Applicability of Relithiation Methods to Ni‐Rich Cathodes

When applying relithiation strategies to cathodes with high nickel content, it is important to note that although the six degradation mechanisms (1–6) identified for LCO and NMC532/622 remain valid, they are much more aggressive in materials with high nickel content (Table 2). Therefore, for the successful transfer of relithiation methods to high‐nickel systems, it is necessary not only to mimic the LCO/NMC approaches, but also to counteract the stronger driving forces of all mechanisms (Table 3). A practical way to select and adapt relithiation strategies for high‐nickel‐content layered cathodes is to assess the degree of similarity between this method and the original synthesis method for high‐nickel‐content materials. Modern synthesis of high‐nickel NMC/NCA is based on strictly controlled solid‐phase or flux‐assisted reactions, which typically involve two‐stage heating (high‐temperature grain growth followed by low‐temperature ordering), an oxygen‐rich atmosphere, and carefully controlled temperature gradients [224, 227, 228]. Methods that replicate these key features typically require fewer adjustments when transitioning to high‐nickel synthesis.

SSR and MST are conceptually closest to traditional synthesis. In both cases, lithium is reinserted at elevated temperatures in an oxidizing atmosphere, allowing the transition metal layers to be rearranged and the oxygen sublattice to be restored [224, 227, 229]. Consequently, these methods can often be transferred almost directly from LCO or medium‐Ni systems, provided that the process parameters comply with the restrictions associated with high Ni content (e.g., holding time below ≈ 800°C, control of heating rate, and maintenance of partial oxygen pressure). SSR variants differ in the method of lithium delivery: SSR‐1 (Li_2_CO_3_) is simple but sensitive to oxygen loss; SSR‐2 (LiOH) is more reactive but sensitive to moisture; SSR‐3 (pre‐coating) minimizes lithium gradients and is therefore particularly well suited for cathodes with high nickel content. MST‐2 and MST‐3 use oxidizing or functionalized melts similar to the synthesis of high‐nickel materials from molten salt or flux, and with proper control can stabilize oxygen, restore microstructure, and passivate interfaces.

Methods that do not reflect synthesis conditions but target similar defect groups can also serve as a reliable basis for adaptation. Figure S2 shows the frequency of eliminated degradation mechanisms (No. 1–6) that are considered by each group of methods. SSR and MST demonstrate consistently broad coverage across all six mechanisms, albeit with different internal weight distributions. Hydrothermal techniques are inherently gentler and demonstrate high activity against mechanisms 2, 3, and partially 5 with minimal introduction of new stresses, making them excellent candidates for low‐temperature structure restoration with controlled subsequent O_2_ annealing. Chemical and electrochemical methods exhibit dual behavior depending on whether sintering is applied as a subsequent step. When followed by a thermal treatment, Chem and EC can correct a comparable number of degradation mechanisms to SSR and MST, addressing not only lithium deficiency but also structural defects such as surface reconstruction, cation disorder, and, in some cases microcracks and oxygen instability (Table S1).

In contrast, when Chem or EC methods are used without subsequent sintering, their effectiveness is mainly limited to lithium replenishment and surface‐related processes, primarily affecting mechanisms 1, 5, and 6, with a partial contribution to surface reconstruction (No. 2), while structural defects such as cation displacement and crack formation (No. 3 and 4) remain largely unchanged. This leads to two key conclusions. First, methods structurally analogous to Ni‐rich synthesis (SSR, MST) can be transferred with minimal modification, provided strict thermal and atmospheric windows are observed. Second, methods that effectively eliminate dominant nickel‐rich degradation mechanisms (2, 3, 5, 4, 6), as shown in Figure S2, are strong candidates for adaptation, even if their physical basis differs from synthesis. Hybrid sequences (e.g., EC or Chem for lithium replenishment followed by sintering for structural rearrangement and coating) combine the advantages of gentle lithium recovery and reliable defect elimination, offering the most balanced pathway for the regeneration of degraded nickel‐containing electrodes.

### 3.5 General Comparative Performance Analysis of Relithiation Methods (Group II)

**FIGURE 5:**
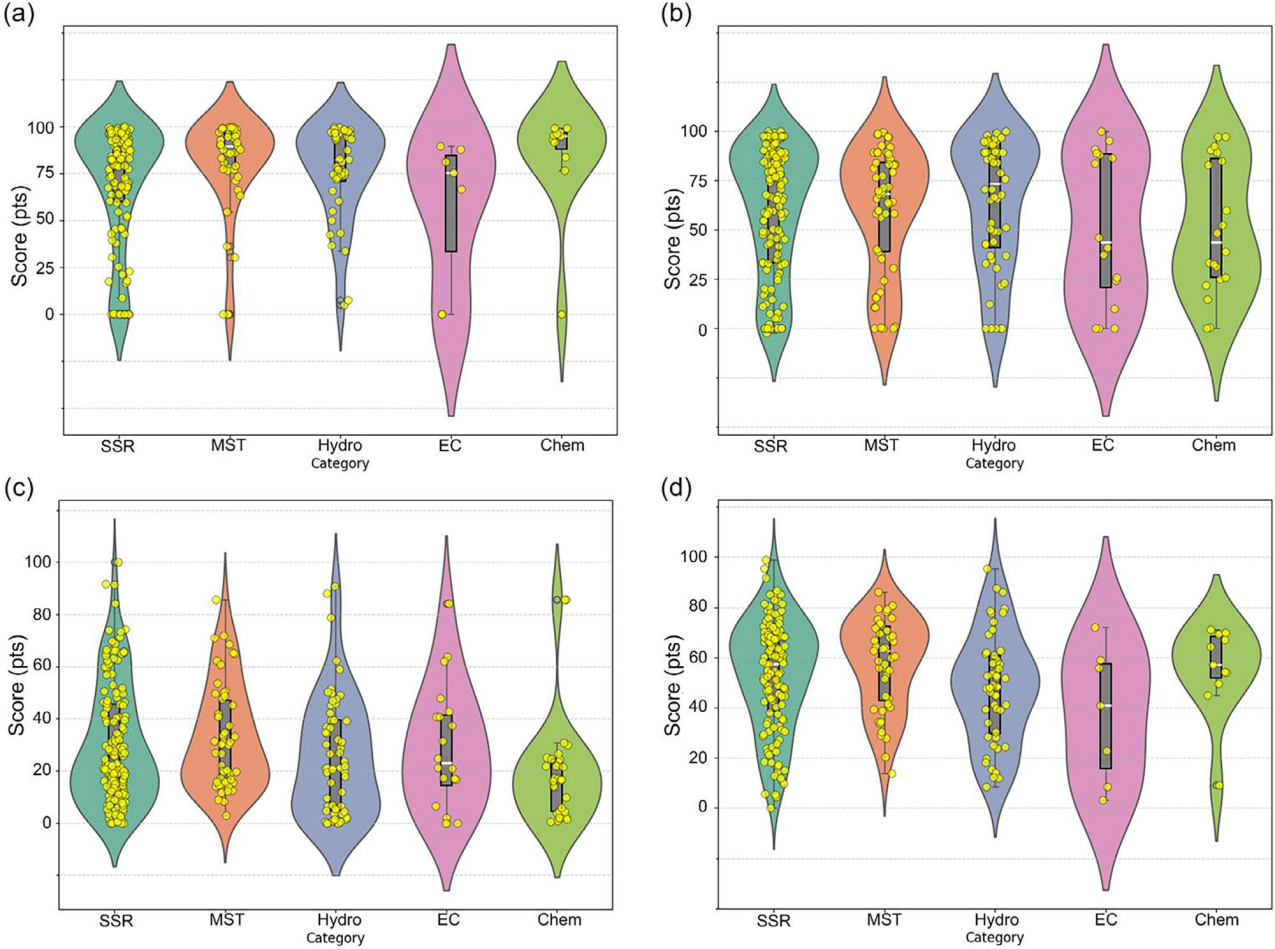
Violin‐boxplots illustrating the score deviations for five relithiation methods with results grouped according to the following criteria: (a) rate capability, (b) cyclic stability, (c) capacity, and (d) the total average score of Group II. SSR—solid‐state relithiation; Hydro—hydrothermal relithiation; MST—molten‐salt thermochemistry; Chem—chemical relithiation; EC—electrochemical relithiation.

Rate‐capability recovery and cycling stability were compared across the main relithiation methodes (Figure 5a,b; Table S5). The median *S_c‐rate_* values follow Chem (95.1) > MST (89.8) > SSR (85.5) > Hydro (82.4) > EC (75.4), indicating that chemically assisted and molten salt pathways provide the most effective restoration of high‐rate performance. In contrast, the *S_cycling_* medians exhibit a different hierarchy, with EC showing the highest cycling stability (84.0), followed by Hydro (73.4), SSR (69.4), and MST (68.4), while Chem yields the lowest median (44.0). The superior performance of EC aligns with findings from our prior work [48], where we similarly evaluated relithiation techniques for lithium iron phosphate (LFP) and observed a phenomenon similar to electrochemical annealing at room temperature. This effect was confirmed in [230] and aimed to enhance the healing of defects in the crystal structure by reducing the activation energy. However, an explanation for the excellent cyclic stability observed for ECs in layered oxide materials is not found. Therefore, further studies are needed to elucidate the effect of electrochemical treatment on the layered oxide cathode materials cyclic stability. The worse result of Chem may be due to the fact that it is based on the regeneration of relatively undamaged electrode materials, i.e., the publication presents relatively good quality materials with weak degradation. This limits the possibility of significant improvement in cycling performance after regeneration.

The differences in the EC and Chem median scores vs. MST, SSR, and Hydro can be attributed to fundamental differences in their respective driving forces. While MST, SSR, and Hydro methods rely primarily on high‐temperature thermal activation to facilitate the relithiation reaction, EC and Chem utilize different mechanisms based on an external electric field and a chemical potential gradient between a lithium source and the cathode material, respectively. Thus, MST, SSR, and Hydro show remarkable similarity in their average performance, reflecting common temperature‐dependent reaction pathways. Specifically, all three techniques involve heating the material, which plays a crucial role in restoring the crystalline structure, facilitating lithium diffusion and mitigating defects. It's plausible that an optimal temperature range, promoting comparable levels of structural regeneration, is achieved across these methods, albeit through distinct processing parameters. Furthermore, the degree to which the crystal structure can be restored, particularly when dealing with heavily degraded cathode materials, may impose an inherent limit on the achievable improvement in cyclic stability. The methods may also have similarities in how they influence particle size and morphology. Lastly, the potential trade‐off between enhanced lithium diffusion and temperature‐induced material degradation is another factor to consider. It is possible that all three methods reach an equivalent equilibrium point between these competing effects, resulting in similar overall performance.

The results of the capacity change assessment are shown in Figure 5c. Among the evaluated relithiation strategies, MST demonstrates the highest median capacity change (30.0), indicating the most significant recovery of discharge capacity when applied to severely degraded cathode materials (SoH ≲ 60%). EC demonstrates an intermediate median score (23.0) comparable to solid‐state relithiation (SSR, 22.8) and hydrothermal treatment (Hydro, 21.0), which mainly work with materials in the intermediate SoH range (≈60–80 In contrast, chemical relithiation has the lowest average score (17.0), indicating that its effect is mainly limited to cathodes with a high residual state of health (SoH) (> 80%), where capacity recovery is essentially limited and improvements mainly arise from surface stabilization rather than the elimination of volume defects.

Given that Group II is an integrated criterion, its reliability depends on the completeness and internal consistency of the underlying electrochemical descriptors. In particular, electrochemical relithiation methods are currently represented by fewer studies that provide a complete set of rate and capacity data, which may lead to a bias in equilibrium aggregation. To address this limitation, we explicitly assessed the statistical robustness of Group II estimates using additional reliability metrics, including interquartile range (IQR), 95% median confidence interval, and leave‐one‐out (ΔLOO) sensitivity.

Figure 5d summarizes the integrated Group II score, defined as the equally weighted average of rate capability, cycling stability, and capacity‐change criteria (1/3 each), and therefore captures the overall electrochemical regeneration effect rather than any single performance dimension. When analyzing the results together with robustness statistics (Table S5), SSR and MST show comparable central tendencies (medians of 61.1 and 64.0, respectively) with similar wide interquartile ranges (≈29 points) and close 95% confidence intervals, indicating that their apparent difference in performance is not significant at the median estimate level. Chem occupies an intermediate position with a median of 57.00, moderate dispersion (IQR 21.0), and relatively high sensitivity using the “leave one out” method (ΔLOO 0.61), indicating that its integrated effectiveness is more dependent on individual studies than in the case of SSR or MST. Hydro has a lower median (48.2) with a wider spread (IQR 31.0), which corresponds to greater variability between hydrothermal implementations. EC has a limited evidence base and, accordingly, shows the highest sensitivity (ΔLOO 0.72) and the highest variance (IQR 52.63), which means that its median (41.0) is significantly more dependent on individual studies than is the case with thermally supported methods. A direct comparison of ΔLOO values shows a clear gradient of statistical reliability, decreasing from Hydro (ΔLOO = 0.38) and MST/SSR (ΔLOO ≈ 0.44–0.45) to Chem (ΔLOO 0.61) and reaching a minimum for EC (ΔLOO = 0.72), reflecting the gradually increasing sensitivity of the median score to individual data points. Thus, when considered together, IQR, 95% confidence intervals of the median, as well as ΔLOO SSR and MST demonstrate the most balanced statistical stability, combining moderate data distribution with stable and clearly defined median values. Hydro demonstrates excellent median stability (lowest ΔLOO) but a more significant interquartile spread, indicating higher variability between studies despite a stable central tendency. Chem and especially EC demonstrate a gradual decline in stability with increasing sensitivity and dispersion. Thus, in general, MST, SSR, and Hydro show the best results in terms of performance recovery of degraded cathode materials.

### 3.6 Comparative Analysis of CO_2_ Emission Relithiation Methods

**FIGURE 6:**
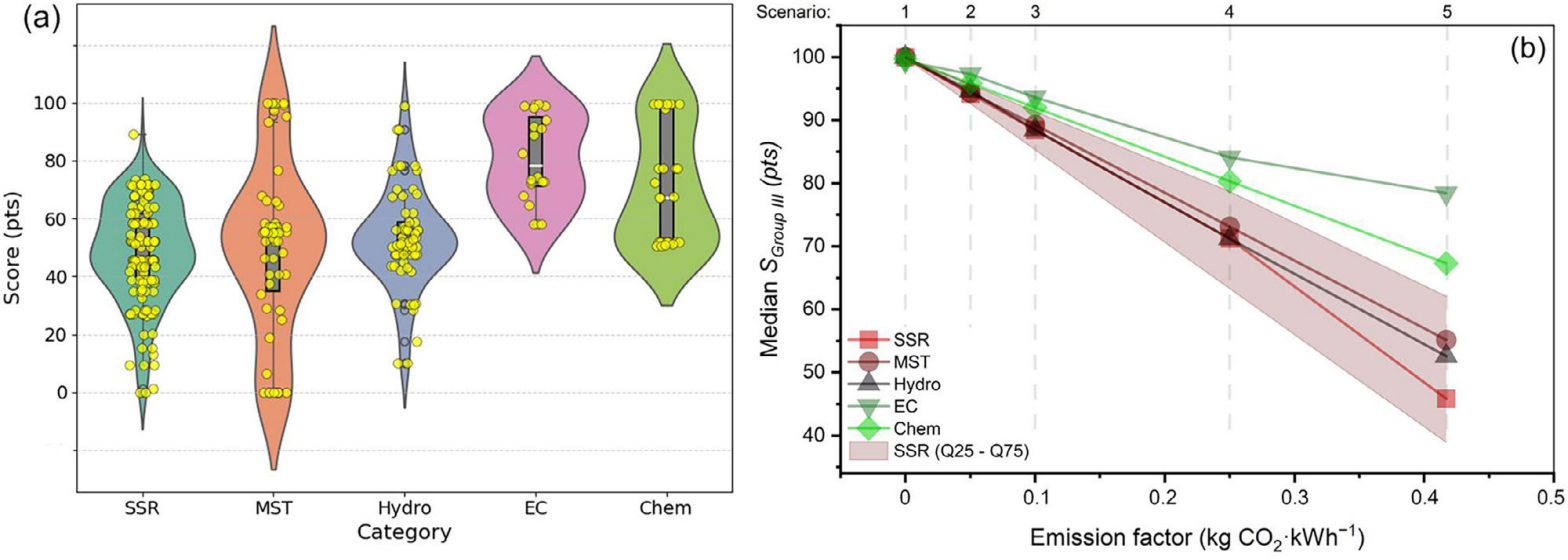
Total boxplot average scores of Group III (a). Median Group III scores as a function of the electricity emission factor (EF) for different relithiation methods (b). Shaded area indicates the interquartile range (Q25–Q75) of the SSR baseline to illustrate typical literature dispersion without overloading the Figure Scenario 1–5 correspond to EF = 0, 0.05, 0.10, 0.25, and 0.417 kg CO_2_·kWh^−1^, respectively. SSR—solid‐state relithiation; Hydro—hydrothermal relithiation; MST—molten‐salt thermochemistry; Chem—chemical relithiation; EC—electrochemical relithiation.

The scoring of the discussed approaches was based on CO_2_‐equivalent emissions from energy consumption and materials used in the relithiation process of layered oxides (Figure 6), following the GHG emissions criteria framework. The median values of total average scores (Group III) for relithiation methods show ratios similar to those of the energy consumption criteria. This correlation is explained by the small contribution of material‐related CO_2_ emissions to the total carbon footprint. The CO_2_ released during chemical reactions in the relithiation process is negligible compared to energy‐related CO_2_ emissions from fuel combustion. Thus, at the subgroup level, the regeneration methods can be arranged in order of the amount of CO_2_ released during the regeneration process: EC<MST‐2<Chem<SSR‐2<MST‐1<Hydro‐2≈Hydro‐1<SSR‐3<SSR‐1≈Hydro‐3<MST‐3. This ranking indicates that the lowest energy consuming methods (e.g., EC) exhibit the smallest carbon footprint, whereas high‐temperature processes are associated with the highest CO_2_ emissions. Therefore, the application of high‐temperature treatment, even as an additional step in the cathode material regeneration process, leads to a significant increase in energy consumption and CO_2_ emissions. The results highlight the importance of optimizing process conditions to reduce the environmental impact of cathode material regeneration.

At the method level, EC and Chem relithiation demonstrate higher average CO_2_ emissions than (SSR), indicating a lower average CO_2_ footprint. EC demonstrates the highest average value (78.45 vs. 45.83 for SSR), but this improvement is accompanied by a decrease in robustness (Table S5). Compared to SSR (IQR = 23.01; ΔLOO = 2.2), EC shows a slightly higher dispersion (IQR = 24.10) and almost twice the sensitivity (ΔLOO = 4.08), indicating a stronger dependence on individual highly effective studies. Chem also outperforms SSR in terms of median score (67.35), but shows a wider spread (IQR = 26.91 vs. 23.01) and an increased ΔLOO value (3.9 vs. 2.2), which corresponds to higher variability between studies. In contrast, Hydro and MST occupy an intermediate position: their median values (52.61 and 55.18, respectively) are higher than those of SSR, while their robustness values remain broadly comparable to those of the solid state (ΔLOO = 2.5–3.1; 95% CI(median) = 50.20–55.95 for Hydro and 52.3–57.1 for MST). The higher heterogeneity of EC and Chem compared to SSR is largely due to the inconsistent application of sintering after relithiation. In many EC/Chem studies, lithium recovery is accompanied by high‐temperature heat treatment to restore crystallinity and soften surface reconstruction, while in other studies, sintering is avoided to minimize energy consumption. This discrepancy leads to significant differences in carbon footprint within the same group of methods, which directly contributes to higher IQR and ΔLOO values compared to SSR. In contrast, SSR inherently includes sintering as a mandatory step, which stabilizes energy consumption across different publications and provides a more reliable median value.

To gain a deeper understanding of the sustainability trends discussed above, scenarios were analyzed with changes in the emissions factor (EF) for electricity from a carbon‐free limit to the baseline energy system mix (Figure 6b; Table S7). The selected scenarios cover the materials‐only limit (EF→0), low‐carbon electricity representative of renewable/nuclear‐dominant supply (EF = 0.05–0.10), a decarbonized medium‐low grid (EF = 0.25), and the baseline grid mix used throughout this work (EF = 0.417), thereby covering the transition from negligible to dominant electricity‐related CO_2_ contributions [231, 232, 233]. When EF→0, all methods converge to near‐perfect scores (median ≈ 100), indicating negligible emissions associated with materials and that the differences observed in the baseline dataset are mainly due to energy consumption. As EF increases to 0.05–0.10, median values decrease but remain high for all routes, with EC and Chem consistently maintaining the highest values. At higher EF values (0.25–0.417), the differences between the groups of methods become more pronounced: methods in which heat treatment dominates (SSR, MST, Hydro) fall within a narrow range of lower median values due to the universal contribution of high‐temperature processing, while EC and Chem retain a clear advantage due to their lower dependence on required sintering. The scenario trends directly confirm the stability analysis presented above. Although EC and Chem outperform SSR in terms of median Group III values for all realistic EF values, they also exhibit a wider interquartile range, reflecting their stronger dependence on recycling implementation (Table S6). Inconsistent application of sintering after relithiation leads to large fluctuations in energy demand and, as a result, to differences in carbon footprints within these groups of methods. In contrast, SSR inherently includes sintering as a fixed step, which stabilizes energy consumption and leads to a more consistent median across all scenarios. Overall, the scenario analysis shows that the qualitative ranking of relithiation methods is robust in terms of electricity carbon oxide emission. In practice, the scenarios point to two levers for decarbonization: (i) decarbonization of the energy system is primarily beneficial for routes dominated by thermal energy (SSR/MST/Hydro), which converge to high values only at low EF electricity, and (ii) process standardization (avoiding unnecessary annealing or reducing its intensity) is most important for EC/Chem, whose average advantage is maintained at all EF values, but whose variability increases when implementation differs between studies.

While a comparison based on CO_2_ equivalent shows that chemical and electrochemical relithiation are the most energy‐efficient and low‐carbon options, an additional toxicity assessment (Section S2, Table S1, and Figure S4) reveals a different trend. The average *S_tox_* values for SSR, MST, Hydro, and EC methods are clustered around 33.3 (BM‐2), which corresponds to moderate hazard, while Chem methods show a significantly lower value of around 22 (BM‐1–BM‐2) due to the use of reactive organolithium compounds, redox mediators, and polar aprotic solvents. Thus, the method with one of the lowest carbon footprints also carries the greatest intrinsic chemical hazard. Conversely, electrochemical relithiation combines low carbon emissions and low toxicity (BM‐2–BM‐3), making it the most balanced option in terms of environmental impact and toxicology.

### 3.7 Integrated Performance Assessment and Perspective Directions for Relithiation Technologies

**FIGURE 7:**
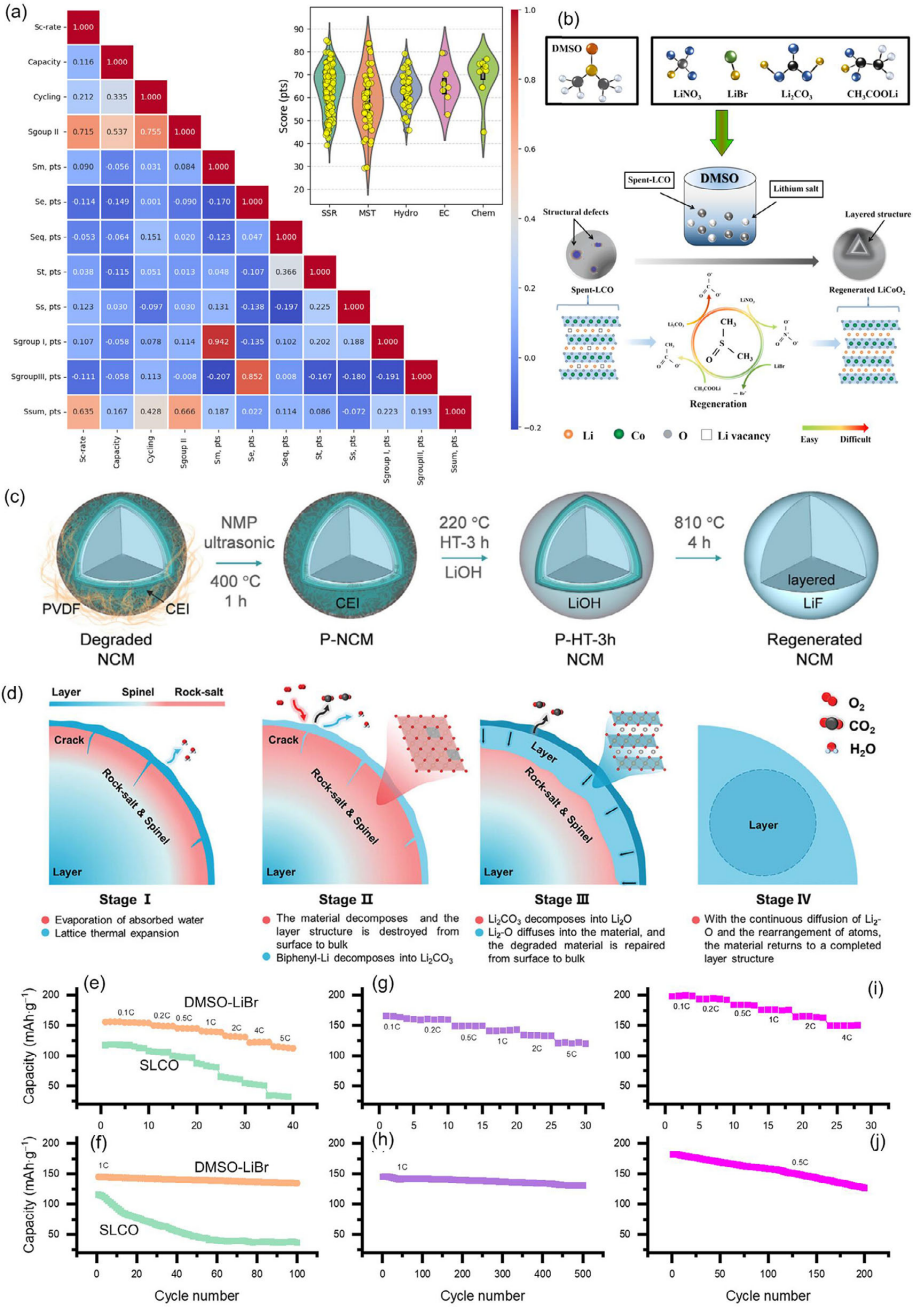
Summarized metrics for four relithiation methods (a); the schematic diagram auto‐oxidative relithiation [22] (b); Reproduced with permission [22]. Copyright 2025 Elsevier. Illustration of the process of regeneration degraded cathode materials through the hydrothermal synergistic molten salt method [146] (c); Reproduced with permission [146]. Copyright 2025 Elsevier. Schematic of the four stages of (R‐NCM83) regeneration process [105] (d); Reproduced with permission [105]. Copyright 2025 John Wiley and Sons. Rate and cycling performance of regenerated materials: [22] (DMSO‐LiBr) (e,f); [146] (regenerate) (g,h); [105] (R‐NCM83) (i,j). SSR—solid‐state relithiation; Hydro—hydrothermal relithiation; MST—molten‐salt thermochemistry; Chem—chemical relithiation; EC—electrochemical relithiation.

Figure 7a shows the Pearson correlation matrix of the major variables for the assessment of CAMs recovery methods. Strong positive intercorrelations are seen between the subscales for *S_Group II_*, which also display high levels of internal consistency (e.g., r = 0.731 with *S_cycling_*) and are positively correlated with the total *S_sum_* score (r = 0.568), suggesting that it contributes significantly to the overall score. *S_Group I_* is also highly correlated with *S_m_* (r = 0.974) and *S_sum_* (r = 0.571). This indicates such cost effects on the efficient relithiation routes that are obtaining better high score methods. However, no significant correlations were found between independent three criteria groups, such as *S_Group I_*, *S_Group II_* and *S_Group III_*. The lack of interdimensional relationships strongly emphasizes the need for a comprehensive, multidimensional evaluation of recasting methods. It also suggests that the conditions and parameters used during recasting cannot reliably predict the resulting electrochemical properties or the overall industrial scalability of a particular method. A comparative analysis of the total averaged scores (inset in Figure 7a) for Group I, II, and III identifies the most promising relithiation methods. Among the four evaluated methods, the Chem method achieves the highest average score (72.3 points), which highlights its efficiency. However, it should be considered that most Chem‐based studies involve a hybrid approach combining chemical relithiation with subsequent sintering. This means that its superiority may be due to synergistic effects.

A comparison of central tendencies and robustness metrics reveals significant differences between the various relithiation methods. The three temperature‐controlled methods produce similar median *S_sum_* values (Tables S4 and S8): SSR, 65.7 points; Hydro, 63.4 points, and MST, 59.6 points, indicating comparable overall performance. However, the robustness of their data varies considerably. Hydro shows the smallest distribution (IQR 8.6; ΔLOO 0.3), indicating stable performance across studies. SSR also shows stable behavior (IQR 14.5; ΔLOO 0.1). By contrast, MST shows a significantly wider distribution (IQR 15.3; ΔLOO 1.2), reflecting its higher sensitivity to specific experimental implementations. At the method variants level, several ones outperform their parent methods, but differ in terms of robustness. SSR‐2 achieves a higher median score of 66.4 points while maintaining good stability (IQR 8.4 points; ΔLOO 0.1), indicating a reproducible performance advantage. MST‐2 achieves an even higher median score (69.8), but with significantly greater variability (IQR 15.6; ΔLOO 0.4), depending more on process details.

In addition, Table 4 shows the repairing time as an interpretive metric for industrial throughput. Although this is already embedded in *S_Group I_* via process duration, highlighting it separately clarifies the time footprint of each relithiation method for line capacity planning. In our dataset, solid‐state and chemical methods typically exhibit the shortest overall cycles: 11.6 h is the median value for Chem, and 9.0 h is for median value for SSR‐3. Hydrothermal and molten salt routes fall in the mid‐range due to autoclave/dual‐step schedules and frequent postanneals, as well as the frequent post‐anneals. In contrast, electrochemical relithiation is the slowest due to the prolonged current‐driven stages. From an industrial perspective, these time profiles refine our Group I assessment. SSR‐3 has the shortest cycle time at 9.0 h, while Chem, at 11.6 h, is comparable to SSR‐1/SSR‐2 rather than being markedly faster. Hydro and MST are mid‐range due to their autoclave/dual‐step schedules and frequent post‐anneals. EC is the slowest due to its prolonged current‐driven stages, so it would benefit from reactor parallelization (multi‐electrode stacks/flow cells) or operating at a higher current density to reduce cycle time.

**TABLE 4:** A summary of Group I—III criteria and total average score for grouped relithiation methods.

Method variations	*S_Group I_*, pts Median	*S_Group II_*, pts Median	*S_Group III_*, pts Median	*S_sum_*, pts Median	Repairing time, h	Ref.
	**Solid‐state relithiation (SSR)**					
SSR‐1	79.8–96.2 pts Median 88.6 pts	5.6–86.6 pts Median 61.5 pts	0.0–73.5 pts Median 45.7 pts	39.2–79.8 pts Median 65.3 pts	0.1 – 30.0 Median 12.5	[25, 26, 27, 62, 63, 64, 65, 66, 67, 68, 69, 70, 71, 72, 73, 74, 75, 76, 77, 78, 79, 80, 81, 82, 83, 84, 85, 86, 87, 88]
SSR‐2	27.9–92.2 pts Median 87.0 pts	0.0–95.4 pts Median 60.2 pts	15.4–73.7 pts Median 58.4 pts	44.5–85.2 pts Median 66.5 pts	8.5 – 34.4 Median 12.3	[25, 63, 68, 84, 89, 90, 91, 92, 93, 94, 95, 96, 97, 98, 99, 100]
SSR‐3	73.6–97.1 pts Median 90.0 pts	4.5–99.0 pts Median 44.0 pts	9.6–64.5 pts Median 50.5 pts	45.8–79.1 pts Median 56.3 pts	4.0 – 30.2 Median 9.0	[63, 72, 82, 101, 102, 103, 104, 105, 106]
	**Molten‐salt thermochemistry (MST)**					
MST‐1	28.1–92.3 pts Median 79.3 pts	13.7–80.9 pts Median 55.0 pts	0.0–100.0 pts Median 52.1 pts	39.7–75.1 pts Median 56.5 pts	7.0 – 31.5 Median 17.5	[107, 108, 109, 110, 111, 112, 113, 114, 115, 116, 117, 118, 119, 120, 121, 122, 123]
MST‐2	23.2–89.1 pts Median 69.0 pts	40.2–86.2 pts Median 65.9 pts	0.0–99.9 pts Median 87.0 pts	23.3–83.8 pts Median 69.8 pts	12.7 – 29.5 Median 20.0	[28, 124, 125, 126, 127, 128, 129, 130, 131]
MST‐3	21.7–89.9 pts Median 52.6 pts	58.6–76.0 pts Median 72.1 pts	0.0–48.2 pts Median 3.4 pts	29.6–62.1 pts Median 49.6 pts	26.5 – 50.7 Median 34.3	[29, 30, 132, 133, 134, 135, 136]
	**Hydrothermal relithiation (Hydro)**					
Hydro‐1	80.2–92.3 pts Median 88.8 pts	8.6–95.6 pts Median 48.2 pts	17.6–78.2 pts Median 50.5 pts	45.9–75.4 pts Median 63.9 pts	4.5 – 41.0 Median 17.5	[31, 33, 137, 138, 139, 140, 141, 142, 143, 144, 145, 146, 147, 148]
Hydro‐2	22.6–92.2 pts Median 88.2 pts	11.8–78.6 pts Median 30.5 pts	10.0–98.8 pts Median 50.8 pts	54.2–76.0 pts Median 59.7 pts	12.0 – 28.5 Median 21.0	[32, 75, 149, 150, 151, 152, 153, 154, 155]
Hydro‐3	62.5–83.4 pts Median 78.8 pts	74.3–79.4 pts Mean 76.9 pts (Insufficient data)	28.2–67.8 pts Median 40.8 pts	68.2–79.2 pts Mean 69.1 pts (Insufficient data)	12.5 – 50.5 Median 35.0	[156, 157, 158, 159]
	**Electrochemical relithiation (EC)**					
EC	16.8–89.2 pts Median 71.9 pts	3.4–72.2 pts Median 41.0 pts	58.1–99.5 pts Median 85.7 pts	52.7–79.6 pts Mean 64.6 pts	5.3 – 94.0 Median 35.5	[19, 20, 21, 160, 161, 162, 163, 164, 165, 166, 167, 168, 169, 170]
	**Chemical relithiation (Chem)**					
Chem	22.6–92.3 pts Median 77.8 pts	8.5–95.6 pts Median 57.0 pts	10.0–98.8 pts Median 67.5 pts	45.9–79.2 pts Median 72.3 pts	4.5 – 20.0 Median 11.6	[22, 23, 24, 171, 172, 173, 174, 175, 176, 177, 178]

These results can consider the perspective directions to develop:

– *Fundamental research* should address mechanistic gaps in EC‐ and Chem‐relithiation, particularly the role of sintering in stabilizing regenerated cathodes. Nowadays, chemical and electrochemical methods are the most unestablished directions that can bring outstanding results into relithiation technologies. The number of publications is significantly lower than those ones of SSR or Hydro, and the Group I, II, III scores are also heterogeneous, giving unexpected direction to develop. Thus, fundamental research has to clarify relation between relithiation reactions and further sintering steps and their synergic impact on electrochemical performance of recycled cathode materials.

From a mechanistic and process‐engineering standpoint, electrochemical relithiation appears to be one of the balanced and conceptually versatile method for direct cathode regeneration. In the current dataset for mixed‐oxide systems, EC achieves a median of 71.9 points in Group I (direct production cost) and 64.6 points in Group III (environmental impact), confirming its inherently moderate material and energy demand and its favorable sustainability profile. Although the available data for Group II (electrochemical characteristics) is still too limited for a statistically valid comparison, existing reports consistently indicate that EC provides high cycle stability (*S_cycling_* ≈ 87.5 points). A similar situation appeared in our previous work on LFP where EC demonstrated high energy efficiency, excellent cycling stability, and the lowest carbon footprint median scores. However, no LFP relithiation method was found that possessed all the desired characteristics, which puts EC on a comparable level of attractiveness with other methods. Thus, the appeal of this method may be due to the specific degradation mechanisms that it effectively eliminates. When implemented without subsequent sintering step, it primarily replenishes lithium and partially reconstructs the surface phase, effectively mitigating mechanisms 2 (surface reconstruction), 3 (cation mixing), and, in some cases, 5 (interphase reactions or oxygen release). This low‐temperature method is attractive for slightly degraded electrodes in which the volume lattice remains largely intact. Conversely, when combined with mild sintering, EC promotes structural rearrangement and oxygen sublattice recovery, extending recovery to mechanisms 4 (crack healing) and 6 (transition metal dissolution). Overall, although electrochemical relithiation is not yet a universal solution, its mechanical flexibility, low environmental impact and ability to function as a mild means of replenishing lithium content make it a promising and practically applicable method for direct regeneration.

– *Industrial R&D* could support Chem, Hydro, SSR, MST methods for pilot‐scale validation, given their balance of performance and scalability. To do this, industrials companies should pay attention on publications with the highest scores obtained among corresponding relithiation methods. These are such as [22] (DMSO‐LiBr), [146] (Regenerate), [105] (R‐NCM83), and [126] (BEU‐80) received 76.8, 75.4, 79.1, and 77.4 points, respectively.

In the study by [22], an innovative auto‐oxidative relithiation process for LiCoO_2_ (LCO) cathode regeneration was demonstrated, employing LiBr as a lithium source and DMSO as both solvent and oxygen donor (Figure 7b). The key innovation lies in the synergistic action of this auto‐oxidative system: DMSO's high ionic facilitates strong Coulombic interactions between constituent ions, while its inherent volatility creates an oxidative atmosphere essential for cathode regeneration. Crucially, DMSO's nucleophilic properties enable efficient Li^+^ release from LiBr, acting as a high‐charge‐flux medium for Li^+^ and O^2^^−^ transport into the degraded cathode lattice. This system outperforms alternatives (e.g., Li_2_CO_3_, CH_3_COOLi, or LiNO_3_ in DMSO) due to its superior solubility, reducibility, and oxidation kinetics. The process achieves dual restoration—lithiation and crystallographic repair—under mild conditions (ambient pressure, low temperature), avoiding aggressive reagents or energy‐intensive steps. Electrochemical testing revealed exceptional recovery: the regenerated LCO exhibited 90.79% capacity retention compared to commercial benchmarks, while rate capability improved by 14× vs. spent LCO (Figure 7e). Structural analysis confirmed the elimination of Co_3_O_4_ impurities and crack healing, attributed to DMSO's oxidative carrier function. Compared to the general Chem method, [22] (DMSO‐LiBr) demonstrates slightly higher values across all evaluated criteria. Its scores for Groups I–III are 83.6, 69.3, and 77.5 points, respectively, which slightly exceed the Chem median values (77.8, 57.0, and 67.5 points). As a result, *S_sum_* is 76.8 compared to 72.3 points for the median case. This indicates that the DMSO‐LiBr chemical relithiation method demonstrates slightly better results in all key aspects compared to most of the described chemical regeneration methods. While this method shows potential for industrial scale‐up of LCO recycling, its applicability to other cathode chemistries (e.g., NCM, LFP) remains untested. Further research should explore mechanistic adaptations for Ni‐ or Mn‐rich systems, solvent recovery for circularity, and cost‐benefit analysis vs. hydrometallurgical routes. Nevertheless, this work establishes a paradigm for sustainable direct regeneration, aligning with low‐carbon recycling mandates.

The study by [146] demonstrates an effective two‐stage relithiation strategy combining hydrothermal treatment and molten salt calcination for regenerating degraded NMC523 cathodes. As illustrated in Figure 7c, the process begins with thorough pretreatment involving N‐methyl‐2‐pyrrolidone ultrasonic cleaning and annealing to remove surface contaminants (polyvinylidene fluoride/carbon residues) while reducing particle size. Subsequent hydrothermal treatment enables efficient Li^+^ diffusion into the cathode lattice under controlled temperature/pressure conditions, forming a uniform LiOH surface layer that preserves the original particle morphology.

The critical regeneration occurs during high‐temperature calcination (810°C, O_2_/air atmosphere) through two parallel reaction pathways: (1) direct relithiation via LiOH (Li_1−x_Ni_0.5_Co_0.2_Mn_0.3_O_2_ + xLiOH → LiNi_0.5_Co_0.2_Mn_0.3_O_2_ + 0.5xH_2_O + 0.25xO_2_) and (2) molten salt‐assisted mechanism through in situ formed Li_2_CO_3_ (Li_1−x_Ni_0.5_Co_0.2_Mn_0.3_O_2_ + 0.5xLi_2_CO_3_ → LiNi_0.5_Co_0.2_Mn_0.3_O_2_ + 0.5xCO_2_ + 0.25xO_2_). Remarkably, this approach successfully restores the layered crystalline structure throughout the entire phase region, as confirmed by comprehensive structural characterization. The regenerated cathodes exhibit exceptional electrochemical performance, maintaining 90.8% capacity retention up to 500 cycles.

The method's universality is demonstrated through successful application to various degraded samples, including commercial spent NMC532, all showing restored rate capability and > 90% capacity retention. The preserved LiOH surface layer plays a crucial role in reconstructing the rock‐salt phase during high‐temperature treatment. Compared with the hydrothermal approaches summarized in Table 4, [146] (Regenerate) shows a markedly superior electrochemical performance, significantly exceeding the *S_Group II_* scores of Hydro (48.2 pts). Its economic efficiency (S_Group I_ = 88.6 pts) is Hydro (88.2 pts) slightly higher indicating a comparable cost level. Environmentally, the method (S_Group III_ = 42.2 pts) performs slightly below Hydro‐1 (50.5 pts) and Hydro‐2 (50.8 pts) because of higher thermal energy use, but still better than Hydro‐3 (40.8 pts). Overall, the S_sum_ = 75.4 pts confirms that the regeneration process delivers the best balance among all hydrothermal approaches, matching comparable economic efficiency, achieving the highest electrochemical recovery, and maintaining competitive environmental performance.

The developed relithiation process ([105] (R‐NCM83)) utilizes a biphenyl‐Li/ tetrahydrofuran solution synthesized from spent lithium metal battery anodes to enable homogeneous cathode regeneration under exceptionally mild conditions (room temperature, ambient pressure). As illustrated in Figure 7d, this innovative approach facilitates uniform coating of degraded cathode particles, simultaneously addressing lithium deficiency and structural defects, including antisite disorders and phase transformations through subsequent thermal annealing. This methodology represents a significant advancement over conventional regeneration techniques by combining waste lithium recycling with cathode restoration while eliminating the need for extreme processing conditions. The experimental procedure involves three critical steps: synthesis of the lithium source through dissolution of spent Li foil in a biphenyl/THF solution (1:1 molar ratio) under inert atmosphere, controlled coating of degraded cathode material with precise lithium stoichiometry (1:0.8–1.4 = Li:cathode ratio) and final structural reconstruction through optimized thermal treatment (700°C–850°C, O_2_/air atmosphere, 5–10 h). This comprehensive process yields regenerated cathodes with exceptional electrochemical performance, as demonstrated by the 181.6 mAh∙g^−1^ initial capacity achieved for NCM83, approaching the 188.2 mAh∙g^−1^ value of pristine material. The regenerated cathodes exhibit superior rate capability (149.5 mAh∙g^−^^1^ at 4C) and enhanced cycling stability (80.7% capacity retention after 150 cycles at 0.5C), significantly outperforming their pristine counterparts in long‐term performance metrics.

Compared with the solid‐state approaches summarized in Table 4, [105] (R‐NCM83) demonstrates the most pronounced improvement in overall performance, primarily due to its exceptionally high electrochemical performance score. Its S_Group II_ = 99.0 pts greatly surpasses the median values of SSR (61.1 pts), highlighting the outstanding rate capability and cycling stability restoration. Economically, the regeneration process (S_Group I_ = 73.6 pts) remains lower than SSR (88.4 pts) in general, reflecting additional costs associated with the lithium‐organic precursor. Environmentally, its S_Group III_ = 64.5 pts is higher than all SSR medians (45.7–58.4 pts), indicating a moderate reduction in specific energy consumption and improved resource utilization. Overall, with S_sum_ = 79.0 pts against the typical 56–66 pts for the SSR strategies, this one provides the best combination of high electrochemical recovery and acceptable cost‐environment trade‐off.

The study by [126] presents a direct and low‐temperature regeneration technique for spent LiCoO_2_ (SLCO) cathode materials using a novel deep eutectic solvent (DES) system composed of betaine, ethylene glycol lithium, and urea (BEU). This innovative method operates under exceptionally mild conditions – 80 °C and atmospheric pressure, offering a promising alternative to conventional energy‐intensive pyrometallurgical and hydrothermal recycling processes. The regeneration mechanism utilizes a network of hydrogen bonds in DES to selectively replenish lithium and valence correct cobalt in the degraded LCO lattice. The process promotes directional healing of structural defects and lithium vacancies. The relithiated material exhibited restored lattice integrity and a uniform Li distribution, with the Co valence state returning predominantly to +3. Electrochemical testing of the regenerated material demonstrated excellent performance: an initial discharge capacity of 209.9 mAh∙g^−1^ and capacity retention of 87.1% after 200 cycles at 1C. Rate performance was also significantly improved, with 165.9 mAh∙g^−1^ achieved at a 5C rate and 94.9% recovery when returning to 0.1C. In comparison, the degraded SLCO material exhibited only 20.9% retention after 200 cycles, highlighting the extent of functional recovery.

Compared with the molten‐salt approaches summarized in Table 4, [126] (BEU‐80) demonstrates a distinctly different balance of characteristics, combining moderate economic and electrochemical performance with outstanding environmental characteristics. Its *S_Group II_* = 66.6 points exceeds the average values for MST‐1 (55.0 points) and is close to MST‐2 (65.9 points), while remaining lower than MST‐3 (72.1 points). Economically, the method (S_Group I_ = 65.6 pts) is lower than MST‐1 (79.3 pts) but slightly lower than MST‐2 (69.0 pts) and above MST‐3 (52.6 pts), indicating a moderate process cost that remains competitive within MST method. Environmentally, the BEU‐80 route achieves an exceptional S_Group III_ → 100 pts, significantly exceeding the median scores of all MST approaches (55.0–72.1 pts), due to its operation at only 80°C and the use of harmless eutectic components with low volatility. Overall, the resulting S_sum_ = 81.0 pts exceeds the 49–70 pts typical of conventional molten‐salt systems, demonstrating that the BEU‐80 process offers the most sustainable and energy‐efficient variant of MST relithiation.

### 3.8 Evolution of Cathode Relithiation Research: Literature Trends and Critical Trade‐Offs in Cost, Performance, and Sustainability (2018–2025)

**FIGURE 8:**
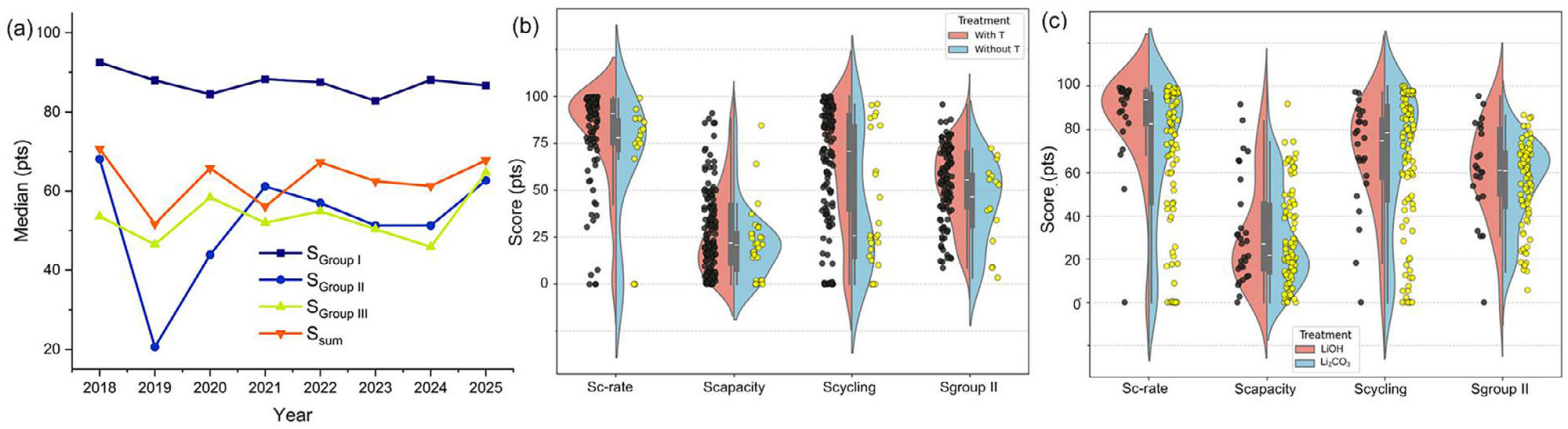
Annual trends in relithiation's cost‐performance‐environment triad (2018–2024) (a); impact of post‐relithiation sintering on the electrochemical performance of cathode materials (b); impact of lithium precursor (LiOH vs. Li_2_CO_3_) on the electrochemical behavior of relithiated cathode materials (c).

Our results provide insights into the trends of critical parameters—recycling cost, electrochemical performance of the recovered materials, and CO_2_ emissions—based on data extracted from recent scientific publications (Figure 8a). These trends are then compared to the current landscape of lithium‐ion battery production and recycling, as outlined in the introduction. The analysis is based on the median values of scores calculated for each year. The median was selected as a statistically robust measure, less sensitive to outliers and asymmetry in the data distribution compared to the mean. This is particularly important for accurately representing typical trends when the data do not follow a normal distribution. Even with the presence of isolated publications reporting abnormally high or low values, the median maintains its representativeness for the majority of studies. The analysis of median values across the three key criterion groups reveals important trends in the development of cathode material relithiation technologies. The data highlight the existence of trade‐offs between process cost, recovery of electrochemical properties, and environmental sustainability, which have become more pronounced in recent years.

**Production Costs (Group I)** show relative stability, maintaining scores between 82.8 and 88.1 from 2020–2025, following an initial decline from the 2018 peak of 92.5 (Figure 8a). This plateau suggests that current approaches have reached limitations in cost reduction, likely due to the fundamental challenges of cathode material regeneration. The marginal fluctuations may reflect shifting balances between capital and operational expenditures in different process designs.

**Performance recovery (Group II)** shows a declining median trend from 68.1 in 2018 to 51.3 in 2023–2024 (Figure 8a) and sharp increase after 2025 to 62.7 pts. This trend does not indicate a generalized loss of relithiation method efficiency, but rather reflects how different degradation mechanisms affect the electrochemical performance of cathode materials and how the set of mechanisms under consideration has evolved (Figure S5). Early studies (2018–2020) focused mainly on volume/compositional defects (Tables 2 and 3): lithium deficiency (mechanism 2), cation mixing/TM migration (mechanism 3) and bulk phase reconstruction (mechanism 4), where correction typically leads to large relative changes in Group II parameters (capacity, τ‐based value and degradation constant k_c_ at cycling). As the field diversifies (2021–2024), the proportion of work devoted to surface/interfacial processes, namely CEI growth/surface passivation (mechanism 5) and surface reconstruction/oxygen vacancy formation (mechanism 6), has increased significantly (Figure S5). However, the 2025 dataset shows a partial shift back toward research focused on bulk processes: mechanism 2 = 23.7%, mechanism 3 = 20.7%, and mechanism 4 = 18.7%, while surface/interfacial mechanisms remain significant (mechanism 5 = 13.5% and mechanism 6 = 11.8%; 25.3% combined). These mechanisms often degrade kinetics more than absolute capacity and often start from less severely degraded states of bulk materials, so the relative improvement between degraded and regenerated electrodes becomes smaller (e.g., more modest changes in *τ* and *k*), even when absolute performance after regeneration is comparable or improved. Accordingly, as the literature shifted toward surface/interfacial degradation modes, the median *S_Group II_* tended to decline due to smaller reported relative deltas between the degraded and regenerated states. Notably, the 2025 dataset shows a partial reversal of this trend: *S_Group II_* increases, consistent with the higher share of bulk‐related mechanisms (2–4) in 2025, for which relithiation and structural repair typically yield larger relative increases in capacity and kinetic descriptors. Overall, the year‐to‐year variations in *S_Group II_* are therefore best interpreted not as a loss of method efficiency, but as a consequence of mechanism mix effects and the changing baseline severity of degradation in the studied materials.

**Environmental Impact (Group III)** exhibits a steady deterioration from 53.6 in 2018 to 45.9 in 2024 (Figure 8a) with a sharp change in trend to 64.7 points in 2025. This negative trend until 2024 indicated an increase in the carbon footprint of new methods, which was likely the result of more energy‐intensive processing stages. The steady decline raises serious questions about the long‐term sustainability of current approaches. However, the increase in the median estimate for group 3 indicates an improvement in the average energy efficiency of regeneration methods.

The *S_sum_* criteria reflects the temporal dynamics of the overall efficiency of cathode regeneration. There has been a decrease in the median *S_sum_* from 70.7 (2018) to 61.3 (2024), with pronounced lows in 2019 (51.7) and 2021 (56.1) (Figure 8a). In contrast, the updated dataset for 2025 shows an increase in *S_sum_* to ∼67–68, interrupting the previously observed downward trend. The recovery coincides with a more balanced contribution from Groups I–III, rather than the dominance of a single group in terms of performance, indicating improved integration of regeneration strategies. These observations suggest that recent literature reflects a shift toward more consistent optimization of cathode regeneration rather than individual metrics. The existing literature indicates that simultaneously reducing costs, restoring productivity, and reducing environmental impact remains a challenge for regeneration based on re‐lithiation. Observed trends show that gradual optimization of existing methods is insufficient for continuous improvement in overall efficiency. Therefore, future progress will require comprehensive approaches that simultaneously consider chemical processes, energy efficiency, and materials engineering, rather than isolated optimization of individual performance indicators.

Considering the widespread use of a subsequent sintering step following relithiation in many studies, a critical question arises regarding the necessity of this step, given that sintering is one of the most energy‐intensive stages, significantly contributing to the process's overall energy consumption and corresponding carbon dioxide emissions. Therefore, Figure 8b presents boxplots comparing the performance evaluations of studies that included the sintering step. This comparison was carried out exclusively for Group II criteria, as the justification for the sintering stage is directly linked to the need for enhanced electrochemical properties of the regenerated cathode material [234, 235].

Although the comparison covers all oxide cathode materials rather than strictly homogeneous groups (e.g., LCO is compared against LCO and LMO against LMO) and materials regenerated without subsequent sintering are compared against all materials regenerated with sintering, the comparison of median distribution values remains valid. This validity arises from the statistical robustness of median values in mitigating the influence of outliers and variations inherent to different cathode chemistries [236, 237]. Furthermore, robustness analysis confirms the stability of central trends: for the with‐T subset, the sensitivity of the “leave one out” method is low across all indicators (ΔLOO(median) ≤ 0.6), indicating that the medians do not depend on individual studies. In contrast, the without‐T subset shows significantly higher sensitivity for *S_c‐rate_* and *S_Group II_* (ΔLOO ≈ 2.5–3.0), which is consistent with smaller sample sizes and wider distribution. The SSR relithiation pathway was excluded from this particular comparison because it is mainly indicated as the primary regeneration pathway.

As shown in Figure 8b (Table S9), the inclusion of the temperature stage is associated with systematically higher median values, which is most evident in terms of speed capability and cyclic stability. The average speed *S_c‐rate_* increases from 77.8 (without T) to 94.7 (with T), and the average cyclic stability *S_cycling_* increases from 25.8 to 86.6, demonstrating that heat treatment is a dominant factor contributing to the recovery of kinetics and stabilization of regenerated cathodes. In contrast, the median *S_capacity_* changes only slightly (from 20.8 to 22.0), indicating that the temperature step primarily improves kinetic/structural factors rather than low‐rate capacity recovery. In line with these trends, the composite index *S_Group II_* increases from 46.5 (without T) to 57.9 (with T). From a mechanical point of view, this behavior corresponds to the established role of heat treatment in stimulating recrystallization, reducing volume/surface disorder, and improving lithium‐ion transport pathways and electronic connectivity, which primarily has a positive effect on speed characteristics and cycle stability, and not just on capacity [234, 235, 238].

However, given the considerable impact of sintering on Group III criteria, particularly concerning energy consumption and greenhouse gas emissions, it is imperative to optimize this step. To mitigate the environmental burden while maintaining performance benefits, it is recommended to lower the sintering temperature and reduce the duration of thermal treatment. Additionally, implementing advanced sintering equipment with higher energy‐to‐heat conversion efficiency, such as microwave or spark plasma sintering, could further minimize energy consumption and emissions. These optimizations would ensure that the benefits of sintering in enhancing electrochemical performance are achieved without compromising the environmental sustainability of the relithiation process.

In SSR relithiation, the most commonly used lithium precursors are Li_2_CO_3_ and LiOH. We quantitatively assessed how the lithium source affects the electrochemical performance of relithiated CAM (Figure 8c; Table S9). In terms of rate capability, LiOH shows a higher central tendency (median rate *S_c‐rate_* is 93.6 compared to 82.4 for Li_2_CO_3_), and the LiOH dataset is also more internally consistent (IQR 14.5 compared to 50.0), indicating even more reproducible results across fewer studies than for Li_2_CO_3_. Regarding cycle stability, the medians are comparable (*S_cycling_* 74.6 for LiOH vs. 78.2 for Li_2_CO_3_), with LiOH again showing a narrower spread (IQR 26.6 vs. 49.1). In addition, the median capacity recovery for LiOH is slightly higher (*S_capacity_* is 27.0 vs. 22.5), although both precursors show significant dispersion (IQR 30.0 for LiOH and 31.4 for Li_2_CO_3_), reflecting high sensitivity. The aggregate median score is practically indistinguishable between studies with two types of lithium precursors. *S_Group II_* is 60.0 (LiOH) and 61.5 (Li_2_CO_3_) with overlapping confidence intervals (53.7–82.4 vs. 57.4–66.6, respectively), indicating that, based on the available literature, the average electrochemical characteristics of recovered CAMs cannot be statistically distinguished between SSRs using LiOH or Li_2_CO_3_. Therefore, the precursor choice should be determined by the subsequent application of CAMs and economic feasibility.

These quantitative trends can be rationalized by considering the intrinsic physicochemical properties of the lithium precursors and their behavior during high‐temperature lithiation. The studies [239, 240] have demonstrated that LiOH exhibits lower decomposition temperature and superior stability, making it particularly suitable for NCM cathode preparation and facilitating the formation of materials with more uniform particle distribution. The preferential performance of LiOH in nickel‐rich layered oxide cathodes (NCA, NCM) can be attributed to several key factors: (1) enhanced tap density and crystallinity achieved at lower sintering temperatures, (2) reduced Li^+^/Ni^2^^+^ cation mixing, which improves structural stability and (3) minimized formation of residual lithium compounds (e.g., Li_2_CO_3_, Li_2_O) that can adversely affect slurry processing and cell performance. These advantages are particularly pronounced in high‐nickel compositions (Ni ≥ 60%), establishing LiOH as the preferred lithium source for high‐energy‐density cathode regeneration in next‐generation battery applications.

### 3.9 Promising Alternatives to Li‐Ion: Growing Research and Future Recycling Needs

Lithium‐ion batteries (LIBs) currently dominate the energy storage landscape, powering everything from portable electronics to electric vehicles. However, their widespread adoption has exposed critical challenges, including resource scarcity, environmental concerns from toxic components like cobalt, and complex recycling processes [241]. As the demand for sustainable energy storage grows, alternative battery technologies—such as zinc‐ion (ZIBs) and sodium‐ion (SIBs) batteries—are gaining traction due to their inherent safety, lower cost, and reduced environmental impact [242]. Yet, the rapid development of these emerging technologies necessitates proactive planning for their end‐of‐life management to avoid the recycling bottlenecks currently faced by LIBs.

**FIGURE 9:**
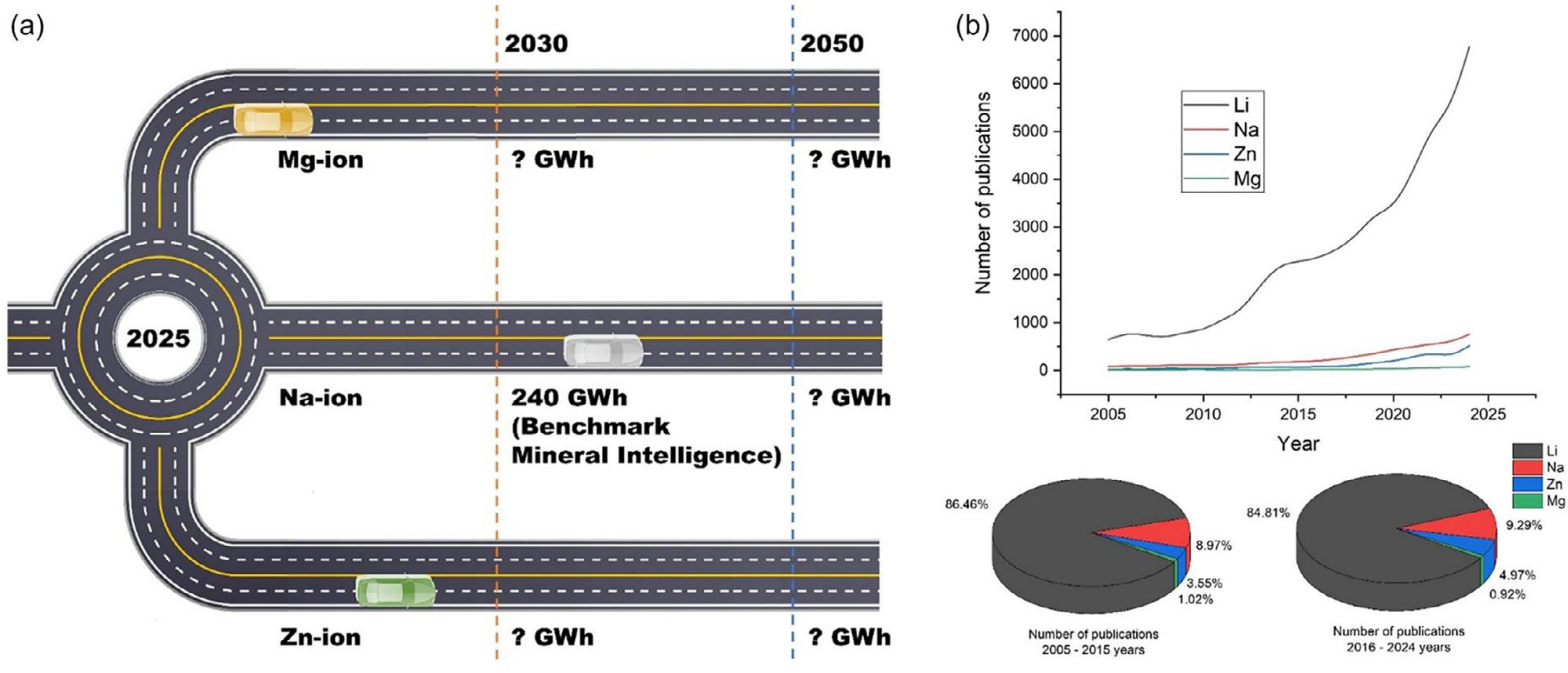
Projected trajectories of emerging battery chemistries and their recycling potential toward 2050; Dynamics of Li‐, Na‐, Zn‐, and Mg‐ion battery materials publications from 2005 to 2024 based on Science Direct data.

Zinc‐ion batteries (ZIBs), for instance, leverage abundant materials like zinc and aqueous electrolytes, offering a compelling balance of energy density (75–85 Wh∙kg^−1^) and operational safety [243]. While their performance trails behind LIBs (180–230 Wh∙kg^−1^), ZIBs achieve theoretical capacities up to 589 mAh∙g^−1^ with vanadium oxide cathodes [244] and demonstrate practical potential in CR2032 coin cells (458.7 Wh∙kg^−1^ [245]). These characteristics make them particularly promising for stationary storage and wearable electronic [246], where safety and cost outweigh the need for ultrahigh energy density. Similarly, sodium‐ion batteries (SIBs) are poised for commercialization with projected demand reaching 240 GWh by 2030 (Benchmark Mineral Intelligence). However, the recycling infrastructure for these alternatives remains underdeveloped, mirroring the delayed recycling efforts that now plague LIBs (Figure 9a).

This section highlights the urgency of integrating recycling strategies into the development lifecycle of next‐generation batteries. By analyzing publication trends (Figure 9b), we demonstrate the accelerating research interest in ZIBs and SIBs, underscored by advances in cathode materials like δ‐MnO_2_ and layered vanadium oxides [247, 248]. Yet, without parallel advancements in recycling technologies—such as optimizing electrolyte systems (e.g., ZnSO_4_ vs. Zn(CF_3_SO_3_)_2_ [249])—these systems risk repeating the sustainability challenges of their predecessors. We argue for a proactive approach: designing batteries with circularity in mind, optimizing material recovery processes, and establishing regulatory frameworks to ensure scalable recycling solutions are in place before mass adoption.

The transition to post‐Li‐ion batteries is not just a technical evolution but a systemic shift requiring harmonized efforts across research, industry, and policy. By addressing recycling needs today, we can secure the environmental and economic benefits of these promising alternatives tomorrow.

### 3.10 Future Roadmap

**FIGURE 10:**
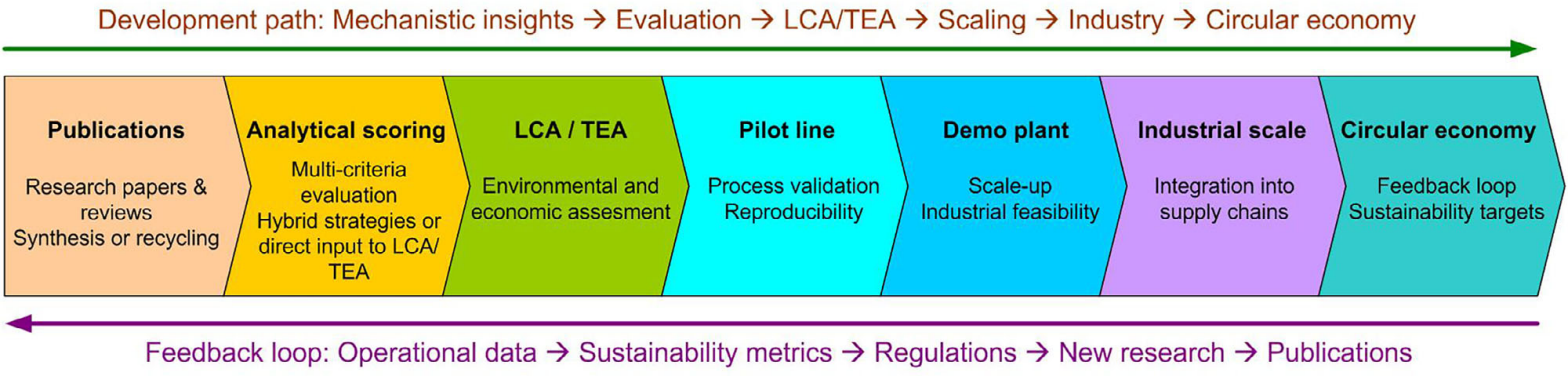
Roadmap for advancing direct relithiation methods illustrating the dual dynamics of the development path (green, top) and the feedback loop (purple, bottom).

The pathway from laboratory studies to industrial deployment of direct relithiation methods can be conceptualized as a multi‐stage roadmap (Figure 10). At the first stage, academic publications and reviews provide fragmented insights into reaction mechanisms and experimental conditions. Here, progress requires a deeper mechanistic understanding, which can be reinforced by systematic quantification of electrochemical properties. In our framework, Group II criteria serve as a numerical proxy for service‐relevant performance, enabling direct correlation between physicochemical changes in the material and shifts in capacity, cycling stability, or power capability. Applied longitudinally, this approach also captures chemistry‐specific or temporal research trends, thereby bridging descriptive studies with predictive process design.

The path from laboratory research to industrial implementation of direct relithiation methods can be represented as a multi‐stage roadmap. In the first stage, academic publications and reviews provide a fragmentary picture of reaction mechanisms and experimental conditions. Here, progress requires a deeper understanding of the mechanisms, which can be supported by a systematic quantitative assessment of electrochemical properties. In our concept, Group II criteria serve as a numerical indicator of performance, allowing a direct correlation to be established between physicochemical changes in the material and changes in capacity, cycle stability, or power.

The second stage involves transforming individual case studies into analytical works with multi‐criteria scoring. This framework not only enables a systematic ranking of publications within each relithiation method but also highlights the most promising development trajectories across different approaches. In cases where several methods achieve comparable total scores, the analysis of sub‐criteria can serve as a basis for constructing hybrid process strategies that combine the advantages of individual approaches. For example, in our previous work on LiFePO_4_ regeneration [48], we proposed a hybrid scheme integrating electrochemical and hydrothermal relithiation. The rationale was that electrochemical relithiation could restore lithium stoichiometry and cycling stability while hydrothermal treatment was more effective at reconstructing particle morphology and improving rate capability. As a result, it was expected that the combined strategy would enhance the advantages of both methods, ensuring increased efficiency while reducing resource costs, demonstrating how multi‐criteria assessment can serve as a guideline for the rational development of new regeneration protocols.

Before laboratory methods are advanced to pilot trials, they should undergo life‐cycle assessment and techno‐economic analysis. These studies provide a critical link between experimental metrics and industrial practice, ensuring that only the most sustainable and economically viable pathways are prioritized. Here, multi‐criteria scoring plays a supporting role: it rapidly screens a wide body of literature and identifies the best candidates, but LCA/TEA provides the rigorous, quantitative basis for decision‐making. In our recent study on V_2_O_5_ cathodes [250], we applied multi‐criteria evaluation to identify the most promising laboratory methods and subsequently carried out a techno‐economic assessment. This analysis yielded realistic cost estimates in the range of 135–177$/kWh, depending on reagent costs and synthesis duration. This demonstrates how our scoring framework can act as a rapid pre‐selection tool while full LCA/TEA confirms the feasibility of specific methods under industrially relevant economic and sustainability constraints.

After receiving support from LCA/TEA, the most viable and highest‐scoring methods should be tested under conditions that meet industrial requirements. This begins with pilot lines where reproducibility, productivity, and operability can be evaluated and continues with demonstration plants to confirm scalability and competitiveness compared to existing processing technologies. At this stage, feedback from operational data and sustainability metrics is fed back into research, closing the loop and aligning academic research with industrial needs and circular economy goals.

Finally, these stages converge in pilot lines, demonstration plants, and ultimately industrial implementation with feedback loops returning operational and environmental data to the assessment system. The ultimate goal of this roadmap is not just industrial implementation, but integration into circular economy strategies, where iterative learning at various scales ensures that direct relithiation evolves from laboratory proof of concept to reliable and sustainable technology.

## 4 Conclusions

**Group I—Techno‐economic efficiency**. SSR and Hydro demonstrate the highest average technical and economic indicators, with SSR‐1/2/3 (S_Group I_ = 88.6/87.0/90.0; IQR = 5.2/6.2/11.5) and Hydro‐1/2 (88.8/88.2; IQR = 3.7/2.9). MST‐1 may be competitive but less reliable (79.3; IQR 25.4). EC has the lowest energy consumption (E = 143.0 kJ·g^−1^) but still suffers from high material costs (147.7$·kg^−1^) and processing time, resulting in a lower score in Group I (71.9; IQR 16.0). Chem combines moderate energy consumption (E = 333.7 kJ·g^−1^) with high material costs (201.4$·kg^−1^), resulting in an intermediate score (77.8; IQR 21.0) and remaining competitive within Group I due to simple equipment and short active stages. Overall, robustness varies greatly across methods: Hydro‐1/2 shows the lowest dispersion (IQR 2.9–3.7), and SSR‐1/2 shows similar stability (5.2–6.2), while MST/EC/Chem is significantly more variable (16.0–25.4).

**Group II—Electrochemical performance**. Generally, MST and SSR demonstrate the highest scores in Group II, with a *S_Group II_* median of 64.0 and 61.1 pts for MST and SSR, respectively, followed by Chem (57.0 pts), Hydro (48.2 pts), and EC (41.0 pts). Chem and MST lead in terms of individual scores, which *S_c‐rate_* medians are 95.1 and 89.8 pts, respectively, while EC leads in terms of cyclic stability (*S_cycling_* = 84.0 pts), followed by Hydro (73.4 pts), SSR (69.4 pts), and MST (68.4). Chem has the lowest cyclic stability (44.0 pts). Capacity recovery is highest for MST (30.0 pts), while EC/SSR/Hydro are comparable (23.0/22.8/21.0 pts), and Chem is the lowest (17.0 pts), which is consistent with Chem being more commonly applied to less severely degraded CAM. The robustness analysis confirms these main trends: SSR and MST have similar dispersion (IQR ≈ 29) and close 95% CI, while EC shows the largest distribution (IQR 52.6) and highest sensitivity (ΔLOO 0.72); Hydro has the lowest ΔLOO (0.38) but a wider IQR (31.0), indicating variability between methods despite a stable median. For Ni‐rich cathodes (e.g., NMC811+), the most applicable strategies are those that replicate key elements of high‐nickel synthesis, like SSR and MST, directly or Hydro combined with controlled thermal treatment in O_2_ atmosphere to restore oxygen sublattice stability and cation order. Chem and EC require short thermal “polishing” to extend CAM lifetime by replenishing lithium and partially restoring the surface.

**Group III — Environmental footprint and toxicology**. As material‐related CO_2_ emissions are minor, the emissions track is energy‐related with the following subgroup order: EC < MST‐2< Chem < SSR‐2< MST‐1< Hydro‐2 ≈ Hydro‐1< SSR‐3< SSR‐1 ≈ Hydro‐3< MST‐3. Method medians: EC shows the lowest footprint (78.5 pts) due to fewer sintering steps; Chem shows an average of 67.4 pts; Hydro, MST, and SSR show lower averages (52.6, 55.2, and 45.8 pts, respectively), reflecting the near‐universal use of sintering. Robustness differs depending on the method: SSR shows relatively stable central tendencies (IQR 23.01; ΔLOO 2.2), while EC and Chem show higher variability and sensitivity (EC: IQR 24.10; ΔLOO 4.08; Chem: IQR 26.91; ΔLOO 3.9), reflecting inconsistencies in thermal step implementation after relithiation. Hydro and MST occupy an intermediate position, with medians above SSR and broadly comparable robustness (Hydro 95% CI(median) 50.20–55.95; MST 52.3–57.1; ΔLOO ∼2.5–3.1). The toxicology results (S_tox_) indicate that the SSR/MST/Hydro/EC cluster is near 33.3 pts (BM‐2; moderate hazard), whereas the Chem cluster trends toward 22 pts (BM‐1/BM‐2, strong‐to‐moderate hazard) due to the presence of reactive organolithium species, redox mediators, and polar aprotic solvents. Therefore, EC offers the best combined CO_2_–toxicity balance (low footprint, BM‐2–BM‐3), while the low‐CO_2_ potential of Chem must be paired with mediator/solvent redesign to reach BM‐3/BM‐4 safety.

**Exemplars and integrated scores**. The three method medians are close: SSR ≈ 65.7, Hydro ≈ 63.4, and MST ≈ 59.6 pts. Chem leads the integrated assessment with *S_sum_* ≈ 72.3 pts. EC shows *S_sum_* ≈ 64.6 points despite a significantly smaller number of publications. However, this method remains an acceptable alternative for restoring CAM within the current comparison system, although it requires additional time for re‐verification after new studies appear. No method of relithiation for research or subsequent implementation in production should be rejected without prior consideration of the specific conditions. Selection should be based on scalability, flowsheet complexity, and factory suitability. High‐performing exemplars illustrate practical design solutions. Chem [22] (DMSO–LiBr), *S_sum_* ≈ 76.8 pts, is auto‐oxidative and involves a low‐temperature LCO relithiation process that couples Li^+^ replenishment with crystallographic repair, avoiding furnace‐heavy steps. Hydro [146] (Regenerate), *S_sum_* ≈ 75.4 pts) involves hydrothermal prelithiation, followed by a brief molten‐salt anneal that restores layered NMC523 with >90% capacity retention. SSR [105] (R‐NCM83), *S_sum_* ≈ 79.0 pts, involves a recycled biphenyl‐Li/THF coating and a short anneal that achieves a rate/cycling performance close to the benchmark (Group II ≈ 99) for Ni‐rich materials. MST [126] (BEU‐80), *S_sum_* ≈ 81.0 pts, involves a benign DES at 80°C for LCO, delivering top Group III (∼100) performance with competitive electrochemistry.

**Optimization of sintering processes**. Sintering improves electrochemical performance with Group II metrics increasing by 10–25 points. However, this step produces the highest CO_2_ emissions value and contributing 40%–60% of the total environmental impact. Future studies should investigate short‐term sintering techniques, such as microwave or spark plasma sintering, or optimize process parameters (e.g., temperature, time) to identify combinations that maintain performance recovery while reducing environmental impact.

**Preferential usage of LiOH over Li_2_CO_3_**. In SSR relithiation, Li_2_CO_3_ and LiOH are the dominant lithium precursors. LiOH provides a higher average rate capability (93.6 vs. 82.4 pts) and significantly improved reproducibility (IQR 14.5 vs. 50.0). Cycling stability shows comparable medians (74.6 vs. 78.2 pts), but LiOH again demonstrates lower dispersion (IQR 26.6 vs. 49.1). Capacity recovery is slightly higher for LiOH (27.0 vs. 22.5 pts), while both precursors remain sensitive to processing conditions. Importantly, *S_Group II_* is not statistically separable (60.0 vs. 61.5 pts with overlapping 95% CIs), implying that either precursor can achieve similar average Group II performance. From an economic perspective, within Group I, SSR‐2 (LiOH) is associated with lower average energy consumption than SSR‐1 (Li_2_CO_3_) (429.3 vs. 560.0 kJ·g^−1^), while maintaining a similarly high techno‐economic indicator (*S_Group I_* 87.0 vs. 88.6). Thus, the choice of precursor should be determined by the subsequent application of CAM and economic feasibility at a specific plant or region.

**Holistic, innovation‐driven roadmap**. Between 2018 and 2024, the *S_sum_* score declined from 70.7 to 61.3, mainly due to a decline in Group II (to ∼51.3 in 2023–2024) and a weakening of Group III's influence (45.9 in 2024). The 2025 update interrupts this downward trend: *S_sum_* rises to ∼67–68 on the back of a partial recovery in Group II and a notable improvement in Group III, indicating that progress on several criteria is achievable but not yet sustainable. This non‐monotonic evolution motivates the development of a roadmap that links dominant degradation mechanisms to Group II‐relevant metrics, uses Group I/II/III assessments to develop complementary hybrid solutions, and promotes only those technology pathways that pass realistic TEA/LCA checks for pilot demonstration and scaling. LCA checks for pilot demonstration and scaling. Stage 1 (mechanism → metrics): improve mechanistic understanding and quantify performance using Group II as a service‐relevant proxy (e.g., capacity (*Q*), c‐rate (*τ*), and cycling (*k_c_*), establishing a link between physical and chemical repair to electrochemical gains. Stage 2 (multi‐criteria design): use Group I/II/III scoring to rank options and create hybrids where the sub‐criteria complement each other (for example, EC/Chem for stoichiometry control and a brief sintering for bulk repair). Stage 3 (LCA/TEA gate): only flowsheets that meet sustainability and cost thresholds under realistic assumptions should be advanced Stage 4 (pilot → demonstration → scaling up): validate reproducibility, throughput, and operability; feed operational and environmental data back into the scoring framework to close the loop and inform iterations. Within this strategy, EC and Chem remain promising due to their low thermal stoichiometry control, but targeted optimization of lithium diffusion kinetics, phase transformations, and surface reconstruction is required. Economic targets include: reducing the cost of EC materials to increase Group I above ∼70 while maintaining the 94.7 kJ·g^−^^1^ advantage; simplifying Chem flowsheets and mediators to reduce the ∼238$·kg^−^^1^ burden; designing “EC → short SSR” or “Hydro → EC” sequences that decouple room‐temperature recovery from a brief high‐temperature polishing.

## Author Contributions

E.B. contributed to conceptualization, methodology, formal analysis, investigation, resources, supervision, data curation, validation, writing of the original draft, writing, reviewing, and editing. E.E. contributed to formal analysis, writing, reviewing, and editing. A.S. and V.K. contributed to formal analysis. A.P. contributed to investigation, formal analysis, data curation, and writing of the original draft. S.E. and L.S. contributed to the writing of the original draft. Y.K.G. contributed to writing, reviewing, and editing. V.R. contributed to conceptualization, methodology, formal analysis, investigation, data curation, validation, visualization, writing of the original draft, writing, reviewing, and editing.

## Funding

The work was performed in accordance with the state assignments of Federal Research Center of Problems of Chemical Physics and Medicinal Chemistry, Russian Academy of Sciences (No. 125033104607‐0 (search, analysis and processing of data from literature concerning performance criteria (discharge rate, cyclic stability and productivity) of materials relithiated by sintering and drying methods) and 124013000692‐4 (search, analysis and processing of data from literature concerning performance criteria (discharge rate, cyclic stability and productivity) of materials relithiated by electrolysis and hydrothermal treatment or molten salt thermochemistry).

## Conflicts of Interest

The authors declare no conflicts of interest.

## Supporting information




**Supporting File**: advs74373‐sup‐0001‐SuppMat.docx.

## Data Availability

The data that support the findings of this study are available from the corresponding author upon reasonable request.
