# Supplementary material for: Direct Recycling of Mixed‐Oxide Cathodes: Balancing Cost, Performance and Environmental Trade‐Offs
Source: Adv Sci (Weinh). 2026 Feb 16;13(17):e19076. doi: 10.1002/advs.202519076 (PMC13042407; doi:10.1002/advs.202519076)
Supplement: Supplementary file 1 — Supporting File: advs74373‐sup‐0001‐SuppMat.docx. [file ADVS-13-e19076-s001.docx]

**Supporting information**

**Direct Recycling of Mixed-Oxide Cathodes: Balancing Cost, Performance and Environmental Trade-offs**

**Evgenii Beletskii ^1,2*^, Elizaveta Evshchik ^2^, Anna Shikhovtseva ^2^, Valery Kolmakov ^2^, Andrey Popov ^3^, Svetlana Eliseeva ^3^, Lyubov Shmygleva ^2^,** **Yurii K. Gun'ko^4^, Valentin Romanovski ^5,*^**

^1^ MllT Key Laboratory of Critical Materials Technology for New Energy Conversion and Storage, School of Chemistry and Chemical Engineering, Harbin Institute of Technology, Harbin 150001, China

^2^ Federal Research Center for Chemical Physics and Medicinal Chemistry, Russian Academy of Sciences, Academician Semenov Avenue 1, Chernogolovka, Moscow Region 142432, Russia

^3^ Institute of Chemistry, St. Petersburg State University, Universitetskaya Nab. 7/9, St. Petersburg 199034, Russia

^4^ Department School of Chemistry, CRANN and AMBER Research Centres, Institution Trinity College Dublin, College Green, Dublin 2, Ireland

^5^ Department of Materials Science and Engineering, University of Virginia, Charlottesville, VA 22904, USA

***** Corresponding authors:

Dr. Evgenii Beletskii [beletskyev@icp.ac.ru](mailto:beletskyev@icp.ac.ru), [belochkin@yandex.ru](mailto:belochkin@yandex.ru)

Dr. Valentin Romanovskii [rvd9ar@virginia.edu](mailto:rvd9ar@virginia.edu)

**List of Tables/Figures**.

**Table S1.** Summary of literature-reported relithiation methods and their quantitative evaluations

| Reference | Year | Method | Type | Method variants | Degradation mechanisms | Li-source | Sintering | S_Group I_, pts | S_Group II_, pts | S_GroupIII_, pts | S_sum_, pts | S_tox_, pts |
| --- | --- | --- | --- | --- | --- | --- | --- | --- | --- | --- | --- | --- |
| ^[1]^ (DMSO-LiNO3) | 2022 | Chem | LCO |  | 1,2,3,4,5 |  | yes | 82.3 | 57.0 | 77.5 | 72.3 | 38.6 |
| ^[1]^ (DMSO-LiBr) | 2022 | Chem | LCO |  | 1,2,3,4,5 |  | yes | 83.6 | 69.3 | 77.5 | 76.8 | 37.8 |
| ^[1]^ (DMSO-Li2CO3) | 2022 | Chem | LCO |  | 1,2,3,4,5 |  | yes | 91.9 | 45.0 | 77.4 | 71.4 | 41.1 |
| ^[1]^ (DMSO-CH3COOLi) | 2022 | Chem | LCO |  | 1,2,3,4,5 |  | yes | 85.5 | 54.1 | 77.2 | 72.3 | 38.9 |
| ^[2]^ (x=0,1 M DBTQ) | 2021 | Chem | NMC 111 |  | 2,3,4,5 |  | yes | 85.5 | - | 52.0 | - | 23.9 |
| ^[2]^ (x=0,2 M DBTQ) | 2021 | Chem | NMC 111 |  | 2,3,4,5 |  | yes | 83.5 | - | 52.0 | - | 23.5 |
| ^[2]^ (x=0,3 M DBTQ) | 2021 | Chem | NMC 111 |  | 2,3,4,5 |  | yes | 81.6 | - | 52.0 | - | 23.0 |
| ^[2]^ (x=0,5 M DBTQ) | 2021 | Chem | NMC 111 |  | 2,3,4,5 |  | yes | 77.8 | - | 52.0 | - | 22.3 |
| ^[2]^ (10 min x=0,5 M DBTQ) | 2021 | Chem | NMC 111 |  | 2,3,4,5 |  | yes | 77.9 | - | 52.0 | - | 22.3 |
| ^[2]^ (30 min x=0,5 M DBTQ) | 2021 | Chem | NMC 111 |  | 2,3,4,5 |  | yes | 77.9 | - | 52.0 | - | 22.3 |
| ^[2]^ (60 min x=0,5 M DBTQ) | 2021 | Chem | NMC 111 |  | 2,3,4,5 |  | yes | 77.8 | - | 52.0 | - | 22.3 |
| ^[3]^ (RLCO) | 2021 | Chem | LCO |  | 2,3,5 |  | no | 27.5 | - | 99.6 | - | 4.2 |
| ^[3]^ (RNMC622) | 2021 | Chem | NMC 622 |  | 2,3,5 |  | no | 27.6 | - | 99.6 | - | 4.2 |
| ^[4]^ (R-FL-LCO-HT) | 2024 | Chem | LCO |  | 1,2,3,4,5,6 | | yes | 23,1 | - | - | - | - |
| ^[4]^ (R-FL-LCO-Co-HT) | 2024 | Chem | LCO |  | 1,2,3,4,5,6 | | yes | 23,0 | - | - | - | - |
| ^[5]^ (R-LMO) | 2022 | Chem | LMO |  |  |  | no | 63.5 | 54.4 | 98.0 | 72.0 | 16.4 |
| ^[6]^ (NMC811 after regeneration) | 2024 | Chem | NMC 811 |  | 2,3,5 |  | no | 26.5 | 9.0 | 99.6 | 45.0 | 14.5 |
| ^[7]^ (RLCO-8h) | 2024 | Chem | LCO |  | 2,3,4,5 |  | yes | 78.2 | 64.2 | 51.3 | 64.6 | 1.0 |
| ^[8]^ (LPC-2) | 2024 | EC | LMO/NMC | | 1, 2, 3, 6 |  | no | 69,7 | 58.9 | 82.5 | 70.4 |  |
| ^[9]^ (Recycled) | 2020 | EC | LCO |  | 1, 2, 3 |  | no | 89.2 | - | 72.9 | - | 29.4 |
| ^[10]^ (R-NMC) | 2024 | EC | NMC 111 |  | 2, 3 |  | no | 72.8 | - | 72.2 | - | 26.2 |
| ^[11]^ | 2020 | EC | LCO |  | 2, 6 |  | - | - | - | 74.4 | - |  |
| ^[12]^ (R-NMC) | 2023 | EC | NMC 111 |  | 1 |  | no | 66.7 | #ЗНАЧ! | 99.2 | - |  |
| ^[13]^ (D-LCO SOH 90% re-Li) | 2021 | EC | LCO |  | 2, 3, 4, 6 |  | no | 67.8 | 22.9 | 98.8 | 63.2 | 32.4 |
| ^[13]^ (D-LCO SOH 70% re-Li) | 2021 | EC | LCO |  | 2, 3, 4, 6 |  | no | 67.8 | 72.2 | 98.8 | 79.6 | 0.1 |
| ^[14]^ (Regenerated-LCO) | 2011 | EC | LCO |  | 2, 6 |  | no | 16.8 | - | - | - | 0.1 |
| ^[15]^ (LCO-0.93) | 2024 | EC | LCO |  | 2, 3, 6 |  | yes | 52.0 | - | 58.1 | - | 0.1 |
| ^[15]^ (LCO-1.2) | 2024 | EC | LCO |  | 2, 3, 6 |  | yes | 50.9 | - | 58.1 | - |  |
| ^[15]^ (LCO-1.1) | 2024 | EC | LCO |  | 2, 3, 6 |  | yes | 51.3 | - | 58.1 | - | 2.7 |
| ^[16]^ (AC) | 2020 | EC | NMC 413623 | | 1, 2, 3, 5 |  | no | 72,5 | - | - | - | - |
| ^[16]^ (DEC) | 2020 | EC | NMC 413623 | | 1, 2, 3, 5 |  | no | 72,7 | - | - | - | - |
| ^[16]^ (IPA) | 2020 | EC | NMC 413623 | | 1, 2, 3, 5 |  | no | 72,7 | - | - | - | - |
| ^[16]^ (PC) | 2020 | EC | NMC 413623 | | 1, 2, 3, 5 |  | no | 72,5 | - | - | - | - |
| ^[17]^ (R-D-NCM) | 2024 | EC | NMC 622 |  | 2, 3, 6 |  | no | 49.6 | - | 99.5 | - | 9.5 |
| ^[18]^ (R-LCO) | 2020 | EC | LCO |  | 2, 3 |  | yes | 71.3 | - | 72.9 | - | 9.5 |
| ^[19]^ (HS180) | 2023 | Hydro | NMC 151570 | Hydro-1 | 2, 3, 4, 5 |  | yes | 89.0 | 41.3 | 50.8 | 60.4 | 34.7 |
| ^[19]^ (HS200) | 2023 | Hydro | NMC 151570 | Hydro-1 | 2, 3, 4, 5 |  | yes | 89.0 | 48.5 | 50.6 | 62.7 |  |
| ^[19]^ (HS220) | 2023 | Hydro | NMC 151570 | Hydro-1 | 2, 3, 4, 5 |  | yes | 89.0 | 51.7 | 50.4 | 63.7 | 44.9 |
| ^[20]^ (BHLCOE) | 2022 | Hydro | LCO | Hydro-1 | 1, 2, 4, 5, 6 | | yes | 88,6 | 8.5 | 53.4 | 50.2 | 12.7 |
| ^[20]^ (BHLCOE)@LATP) | 2022 | Hydro | LCO | Hydro-1 | 1, 2, 4, 5, 6 | | yes | 80,2 | 54.2 | 53.4 | 62.6 | 12.7 |
| ^[21]^ (R-NCM-15% Al) | 2023 | Hydro | NMC 111 | Hydro-1 | 2, 3, 5 |  | yes | 88.2 | 19.6 | 45.1 | 51.0 | - |
| ^[22]^ (R-NCM-1h) | 2024 | Hydro | NMC 532 | Hydro-1 | 2, 3, 5, 6 |  | yes | 92.3 | 44.9 | 47.7 | 61.6 | 5.7 |
| ^[22]^ (R-NCM-2h) | 2024 | Hydro | NMC 532 | Hydro-1 | 2, 3, 5, 6 |  | yes | 92.2 | 45.1 | 47.7 | 61.6 | - |
| ^[22]^ (R-NCM-4h) | 2024 | Hydro | NMC 532 | Hydro-1 | 2, 3, 5, 6 |  | yes | 92.0 | 53.5 | 47.7 | 64.4 | 4.7 |
| ^[22]^ (R-NCM-6h) | 2024 | Hydro | NMC 532 | Hydro-1 | 2, 3, 5, 6 |  | yes | 91.8 | 52.2 | 47.7 | 63.9 | - |
| ^[22]^ (R-NCM-8h) | 2024 | Hydro | NMC 532 | Hydro-1 | 2, 3, 5, 6 |  | yes | 91.6 | 49.0 | 47.7 | 62.8 | 3.0 |
| ^[22]^ (R-NCM-10h) | 2024 | Hydro | NMC 532 | Hydro-1 | 2, 3, 5, 6 |  | yes | 91.4 | 48.0 | 47.7 | 62.4 | 3.0 |
| ^[22]^ (R-NCM-12h) | 2024 | Hydro | NMC 532 | Hydro-1 | 2, 3, 5, 6 |  | yes | 91.2 | 47.9 | 47.7 | 62.3 | 3.0 |
| ^[23]^ (HS-C-NCM111-GA) | 2022 | Hydro | NMC 111 | Hydro-2 | 2, 3, 4, 5 |  | yes | 87.1 | - | 50.2 | - | 3.0 |
| ^[23]^ (HS-C-NCM622-GA) | 2022 | Hydro | NMC 111 | Hydro-2 | 2, 3, 4, 5 |  | yes | 87.1 | - | 50.2 | - | 3.0 |
| ^[24]^ (HLCO-NM-5%) | 2023 | Hydro | LCO | Hydro-2 | 2, 3, 5, 6 |  | yes | 85.9 | 52.9 | 43.6 | 60.8 | 32.9 |
| ^[24]^ (HLCO-NM-0%) | 2023 | Hydro | LCO | Hydro-2 | 2, 3, 5, 6 |  | yes | 87.5 | 39.7 | 43.7 | 57.0 | - |
| ^[25]^ (Li2CO3-850) | 2021 | Hydro | NMC 111 | Hydro-2 | 2, 3, 5 |  | yes | 88.2 | 28.5 | 50.2 | 55.6 | - |
| ^[25]^ (Li2CO3-750) | 2021 | Hydro | NMC 111 | Hydro-2 | 2, 3, 5 |  | yes | 88.5 | 18.2 | 62.1 | 56.3 | 35.0 |
| ^[25]^ (Li2CO3-650) | 2021 | Hydro | NMC 111 | Hydro-2 | 2, 3, 5 |  | yes | 88.7 | 15.5 | 70.3 | 58.2 | 39.2 |
| ^[25]^ (Li2CO3-550) | 2021 | Hydro | NMC 111 | Hydro-2 | 2, 3, 5 |  | yes | 88.9 | 13.5 | 76.8 | 59.7 |  |
| ^[25]^ (LiOH-850) | 2021 | Hydro | NMC 111 | Hydro-2 | 2, 3, 5 |  | yes | 88.3 | 23.8 | 50.3 | 54.1 | 19.0 |
| ^[25]^ (LiOH-750) | 2021 | Hydro | NMC 111 | Hydro-2 | 2, 3, 5 |  | yes | 88.6 | 24.4 | 62.1 | 58.4 | 19.0 |
| ^[25]^ (LiOH-650) | 2021 | Hydro | NMC 111 | Hydro-2 | 2, 3, 5 |  | yes | 88.8 | 14.1 | 70.3 | 57.7 | 19.0 |
| ^[25]^ (LiOH-550) | 2021 | Hydro | NMC 111 | Hydro-2 | 2, 3, 5 |  | yes | 89.0 | 11.8 | 76.8 | 59.2 | 19.0 |
| ^[26]^ (rCAM) | 2023 | Hydro | NMC 111 | Hydro-1 | 1, 2, 3, 5, 6 | | yes | 85,1 | 12.3 | 51.9 | 49.8 | 18.4 |
| ^[27]^ (0.02 M HT LMO) | 2020 | Hydro | LMO | Hydro-1 |  |  | no | 84.4 | 34.0 | 78.2 | 65.5 | 18.4 |
| ^[27]^ (0.1 M HT LMO) | 2020 | Hydro | LMO | Hydro-1 |  |  | no | 84.4 | 39.4 | 78.2 | 67.4 | - |
| ^[27]^ (0.2 M HT LMO) | 2020 | Hydro | LMO | Hydro-1 |  |  | no | 84.4 | 39.2 | 78.2 | 67.3 | 13.3 |
| ^[28]^ (H-A) | 2022 | Hydro | NMC532 | Hydro-1 | 2, 3, 5 |  | yes | 86.1 | 61.5 | 57.2 | 68.3 | 18.3 |
| ^[29]^ (Regenerate) | 2021 | Hydro | NMC532 | Hydro-1 | 1, 2, 3, 5, 6 | | yes | 88,3 | 95.6 | 42.2 | 75.4 | 18.3 |
| ^[29]^ (Regenerate-W) | 2021 | Hydro | NMC532 | Hydro-1 | 1, 2, 3, 5, 6 | | yes | 87,6 | 87.6 | 17.6 | 64.3 | - |
| ^[30]^ | 2023 | Hydro | NMC532 | Hydro-3 | 2, 3, 5, 6 |  | yes | - | - | 51.1 | - | 18.4 |
| ^[31]^ (Recycled) | 2024 | Hydro | NMC 111 | Hydro-1 | 2, 3, 5, 6 |  | yes | 86.0 | - | 41.7 | - | 21.2 |
| ^[32]^ (Regenerated) | 2023 | Hydro | NMC151570 | Hydro-1 | 2, 3, 4, 5 |  | yes | 87.0 | 59.1 | 43.2 | 63.1 | 33.3 |
| ^[33]^ (R-NMC) | 2023 | Hydro | NMC553015 | Hydro-1 | 2, 3, 4, 5, 6 | | yes | 85,5 | 57.9 | 55.7 | 66.4 | 18.4 |
| ^[34]^ (R-S-NCM) | 2024 | Hydro | NMC532 | Hydro-3 | 1, 2, 3, 4, 5, 6 | | yes | 83,4 | - | 30.7 | - | 18.4 |
| ^[34]^ (F-NCM) | 2024 | Hydro | NMC532 | Hydro-3 | 1, 2, 3, 4, 5, 6 | | yes | 83,4 | - | 30.7 | - | - |
| ^[34]^ (UR-S-NCM ) | 2024 | Hydro | NMC532 | Hydro-3 | 1, 2, 3, 4, 5, 6 | | yes | 62,5 | - | 28.2 | - | 23.0 |
| ^[35]^ (R-LNMO) | 2024 | Hydro | LiNi0.5Mn1.5O4 | Hydro-1 | 2, 3, 4, 5 |  | yes | 82.3 | 25.0 | 30.4 | 45.9 | 0.7 |
| ^[35]^ (R-LNMO-4) | 2024 | Hydro | LiNi0.5Mn1.5O4 | Hydro-1 | 2, 3, 4, 5 |  | yes | 82.2 | 41.1 | 30.4 | 51.3 | 0.7 |
| ^[36]^ (R-NCM) | 2025 | Hydro | NMC622 | Hydro-3 | 1, 2, 3, 4, 5, 6 | | yes | 74,3 | 74.3 | 56.0 | 68.2 | - |
| ^[36]^ (R-NCM-5%LTO) | 2025 | Hydro | NMC622 | Hydro-3 | 1, 2, 3, 4, 5, 6 | | yes | 74,3 | 79.4 | 56.0 | 69.9 | 19.0 |
| ^[37]^ (RHP-LiNO3) | 2023 | Hydro | LCO | Hydro-2 | 1, 2, 3, 5, 6 | | no | 26,8 | 52.9 | 90.9 | 56.9 | - |
| ^[37]^ (RAP-LiNO3) | 2023 | Hydro | LCO | Hydro-2 | 1, 2, 3, 5, 6 | | no | 52,7 | 57.5 | 90.9 | 67.0 | 26.0 |
| ^[38]^ (AM-30) | 2022 | Hydro | LCO | Hydro-1 | 2, 3, 4, 5, 6 | | yes | 89,5 | 60.7 | 56.2 | 68.8 | - |
| ^[38]^ (AM-45) | 2022 | Hydro | LCO | Hydro-1 | 2, 3, 4, 5, 6 | | yes | 89,4 | 62.8 | 56.2 | 69.5 | 32.8 |
| ^[38]^ (AM-60) | 2022 | Hydro | LCO | Hydro-1 | 2, 3, 4, 5, 6 | | yes | 89,4 | 55.4 | 56.2 | 67.0 | 0.6 |
| ^[38]^ (AM-120) | 2022 | Hydro | LCO | Hydro-1 | 2, 3, 4, 5, 6 | | yes | 89,3 | 46.2 | 56.2 | 63.9 | - |
| ^[38]^ (AHLCOE) | 2022 | Hydro | LCO | Hydro-1 | 2, 3, 4, 5, 6 | | yes | 89,1 | 48.2 | 54.8 | 64.0 | 19.0 |
| ^[39]^ (Regenerated NCM) | 2024 | Hydro | NCM 532 | Hydro-1 | 1, 2, 3, 4, 5, 6 | | yes | 86,3 | 62.1 | 52.0 | 66.8 | 19.0 |
| ^[40]^ (w/o H2O2) | 2024 | Hydro | NCM 532 | Hydro-2 | 1, 2, 3, 4, 5, 6 | | yes | 87,0 | - | 10.0 | - | 19.0 |
| ^[40]^ (w H2O2) | 2024 | Hydro | NCM 532 | Hydro-2 | 1, 2, 3, 4, 5, 6 | | yes | 22,6 | - | 10.0 | - | - |
| ^[41]^ (Hydro 180, 12h + 800, 4h) | 2018 | Hydro | LCO | Hydro-2 | 2, 3, 4, 5, 6 | | yes | 88,0 | 78.2 | 53.0 | 73.0 | - |
| ^[41]^ (Hydro 220, 4h + 800, 4h) | 2018 | Hydro | LCO | Hydro-2 | 2, 3, 4, 5, 6 | | yes | 88,8 | 77.7 | 51.2 | 72.6 | 18.4 |
| ^[42]^ (MS-SA 2h) | 2019 | MST | NCM 532 | MST-1 | 2, 3, 5 |  | yes | 88.0 | 13.7 | 46.4 | 49.4 |  |
| ^[42]^ (MS-SA 4h) | 2019 | MST | NCM 532 | MST-1 | 2, 3, 5 |  | yes | 87.8 | 27.7 | 46.4 | 54.0 | 18.4 |
| ^[43]^ (R-NCM-LiB-C2) | 2020 | MST | NMC 111 | MST-2 | 2, 3, 5 |  | yes | 27.0 | - | 76.8 | - | - |
| ^[44]^ (R-NCM523) | 2023 | MST | NCM 532 | MST-3 | 2, 3, 4, 5 |  | yes | 49.8 | 72.6 | 0.0 | 40.8 | 33.3 |
| ^[45]^ (RNCM) | 2023 | MST | NCM 532 | MST-2 | 2, 3, 4, 5 |  | yes | 83.3 | - | 0.0 | - | 10.7 |
| ^[46]^ (D-LCO-R-H) | 2022 | MST | LCO |  | 2, 3, 4, 6 |  | yes | 25.8 | 72.5 | 52.2 | 50.2 | 10.1 |
| ^[47]^ (R-NCM523) | 2022 | MST | NCM 532 | MST-1 | 2, 3, 4, 5, 6 | | yes | 28,0 | 79.7 | 52.1 | 53.3 | 33.3 |
| ^[48]^ (RA-NCM) | 2021 | MST | NCM 532 | MST-2 | 2, 3, 5 |  | yes | 23.2 | 64.6 | 0.0 | 29.3 | 80.9 |
| ^[49]^ (H-NCM) | 2023 | MST | NCM 532 | MST-2 | 2, 3, 4, 5 |  | yes | 24.8 | 86.2 | 97.2 | 69.4 | 2.1 |
| ^[49]^ (O-NCM) | 2023 | MST | NCM 532 | MST-2 | 2, 3, 4, 5 |  | yes | 23.7 | 79.7 | 95.3 | 66.2 | - |
| ^[49]^ (E-NCM) | 2023 | MST | NCM 532 | MST-2 | 2, 3, 4, 5 |  | yes | 65.7 | 78.3 | 99.1 | 81.0 | 16.4 |
| ^[50]^ (RC) | 2020 | MST | NCM 532 | MST-3 | 2, 3, 5 |  | yes | 21.7 | 66.9 | 0.0 | 29.6 | 4.5 |
| ^[51]^ (Regenerated) | 2020 | MST | LCO | MST-3 | 2, 3, 4, 5 |  | yes | 22.6 | - | 0.0 | - | 12.1 |
| ^[52]^ (R-NCM) | 2023 | MST | NCM 532 | MST-1 | 2, 3, 4, 5, 6 | | yes | 64,2 | 38.6 | 58.3 | 53.7 | 20.2 |
| ^[52]^ (R-NCM-Al 3%) | 2023 | MST | NCM 532 | MST-1 | 2, 3, 4, 5, 6 | | yes | 64,0 | 72.8 | 58.3 | 65.0 | - |
| ^[53]^ (Re-NMC-2) | 2023 | MST | NMC 622 | MST-3 | 2, 3, 5, 6 |  | yes | 24.3 | - | 18.9 | - | - |
| ^[54]^ (R-NCA) | 2022 | MST | NCA | MST-3 | 2, 3, 4, 5 |  | yes | 56.3 | 75.3 | 28.4 | 53.3 | 4.4 |
| ^[54]^ (R-NCM) | 2022 | MST | NCM 532 | MST-3 | 2, 3, 4, 5 |  | yes | 55.2 | 75.9 | 6.4 | 45.8 | 22.7 |
| ^[55]^ (MS0.8) | 2020 | MST | NCM 532 | MST-1 | 2, 3, 4, 5, 6 | | yes | 70,4 | - | 40.9 | - | 7.9 |
| ^[55]^ (MS1.1) | 2020 | MST | NCM 532 | MST-1 | 2, 3, 4, 5, 6 | | yes | 75,8 | - | 40.9 | - | - |
| ^[55]^ (MS4) | 2020 | MST | NCM 532 | MST-1 | 2, 3, 4, 5, 6 | | yes | 86,6 | - | 40.9 | - | 7.9 |
| ^[56]^ (BEU-60) | 2023 | MST | LCO | MST-2 | 2, 3, 5 |  | no | 84.9 | 40.2 | 99.9 | 75.0 | 13.1 |
| ^[56]^ (BEU-80) | 2023 | MST | LCO | MST-2 | 2, 3, 5 |  | no | 84.9 | 66.6 | 99.9 | 83.8 | 13.1 |
| ^[56]^ (BEU-100) | 2023 | MST | LCO | MST-2 | 2, 3, 5 |  | no | 84.9 | 55.7 | 99.9 | 80.2 | 13.1 |
| ^[57]^ (R-S1) | 2020 | MST | NCM 532 | MST-1 | 2, 3, 4, 5 |  | yes | 89.9 | 29.2 | 0.0 | 39.7 | 34.0 |
| ^[57]^ (R-S2) | 2020 | MST | NCM 532 | MST-1 | 2, 3, 4, 5 |  | yes | 91.5 | 34.4 | 0.0 | 41.9 | 34.0 |
| ^[57]^ (R-MS) | 2020 | MST | NCM 532 | MST-1 | 2, 3, 4, 5 |  | yes | 90.3 | 53.1 | 0.0 | 47.8 | 34.0 |
| ^[58]^ (RNCM) | 2024 | MST | NCM 532 | MST-1 | 2, 3, 4, 5, 6 | | yes | 91,7 | 51.4 | 55.5 | 66.2 | 33.3 |
| ^[58]^ (LTO@RNCM) | 2024 | MST | NCM 532 | MST-1 | 2, 3, 4, 5, 6 | | yes | 91,6 | 64.0 | 55.5 | 70.4 | 33.3 |
| ^[59]^ (RLCO750) | 2023 | SSR | LCO | SSR-3 | 2, 3, 4, 6 |  |  | 82.0 | 42.6 | 38.9 | 54.5 |  |
| ^[59]^ (RLCO850) | 2023 | SSR | LCO | SSR-3 | 2, 3, 4, 6 |  |  | 81.7 | 49.1 | 27.5 | 52.8 | 33.3 |
| ^[59]^ (RLCO950) | 2023 | SSR | LCO | SSR-3 | 2, 3, 4, 6 |  |  | 81.3 | 43.5 | 13.1 | 46.0 | 28.5 |
| ^[59]^ (RLCO100) | 2023 | SSR | LCO | SSR-3 | 2, 3, 4, 6 |  |  | 82.4 | 78.6 | 52.1 | 71.0 |  |
| ^[59]^ (RLCO103) | 2023 | SSR | LCO | SSR-3 | 2, 3, 4, 6 |  |  | 82.3 | 75.6 | 52.1 | 70.0 | 33.3 |
| ^[59]^ (RLCO105) | 2023 | SSR | LCO | SSR-3 | 2, 3, 4, 6 |  |  | 82.3 | 72.3 | 52.1 | 68.9 | 33.3 |
| ^[59]^ (RLCO110) | 2023 | SSR | LCO | SSR-3 | 2, 3, 4, 6 |  |  | 82.1 | 74.6 | 52.1 | 69.6 | 33.3 |
| ^[19]^ (SS850-4 ) | 2023 | SSR | NMC 151570 | SSR-1 | 2, 3, 4, 5 | Li2CO3 |  | 96.1 | - | 50.4 | - | 33.3 |
| ^[19]^ (SS850-12) | 2023 | SSR | NMC 151570 | SSR-1 | 2, 3, 4, 5 | Li2CO3 |  | 93.0 | - | 50.3 | - | 33.3 |
| ^[41]^ (750, 12 h) | 2018 | SSR | LCO | SSR-1 | 2, 3, 4, 6 | Li2CO3 |  | 93.4 | 68.1 | 63.5 | 75.0 | 33.3 |
| ^[41]^ (850, 4 h) | 2018 | SSR | LCO | SSR-1 | 2, 3, 4, 6 | Li2CO3 |  | 93.9 | 66.2 | 52.1 | 70.7 | - |
| ^[41]^ (850, 8 h) | 2018 | SSR | LCO | SSR-1 | 2, 3, 4, 6 | Li2CO3 |  | 93.5 | 68.3 | 52.1 | 71.3 | - |
| ^[41]^ (850, 12 h) | 2018 | SSR | LCO | SSR-1 | 2, 3, 4, 6 | Li2CO3 |  | 93.1 | 68.4 | 52.1 | 71.2 | 33.3 |
| ^[41]^ (850, 18 h) | 2018 | SSR | LCO | SSR-1 | 2, 3, 4, 6 | Li2CO3 |  | 92.5 | 69.1 | 52.1 | 71.2 |  |
| ^[41]^ (850, 24 h) | 2018 | SSR | LCO | SSR-1 | 2, 3, 4, 6 | Li2CO3 |  | 91.9 | 65.9 | 52.1 | 70.0 | 33.3 |
| ^[41]^ (950, 12 h) | 2018 | SSR | LCO | SSR-1 | 2, 3, 4, 6 | Li2CO3 |  | 92.7 | 69.2 | 37.7 | 66.5 | 33.3 |
| ^[60]^ (R-NCM523) | 2021 | SSR | NMC 532 | SSR-2 | 2, 3, 6 | LiOH |  | 92.2 | 57.7 | 58.4 | 69.4 | 33.3 |
| ^[61]^ (HLCO100) | 2020 | SSR | LCO | SSR-2 | 2, 3, 4 | LiOH |  | 92.0 | - | 58.4 | - | 33.3 |
| ^[61]^ (HLCO103) | 2020 | SSR | LCO | SSR-2 | 2, 3, 4 | LiOH |  | 91.9 | - | 58.4 | - | 33.3 |
| ^[61]^ (HLCO105) | 2020 | SSR | LCO | SSR-2 | 2, 3, 4 | LiOH |  | 91.6 | - | 58.4 | - | - |
| ^[61]^ (CLCO100) | 2020 | SSR | LCO | SSR-1 | 2, 3, 4 | Li2CO3 |  | 93.7 | - | 58.3 | - | 33.3 |
| ^[61]^ (CLCO103) | 2020 | SSR | LCO | SSR-1 | 2, 3, 4 | Li2CO3 |  | 93.6 | - | 58.3 | - | - |
| ^[61]^ (CLCO105) | 2020 | SSR | LCO | SSR-1 | 2, 3, 4 | Li2CO3 |  | 93.4 | - | 58.3 | - | 33.3 |
| ^[62]^ (B-LCO-MA) | 2023 | SSR | LCO | SSR-1 | 2, 3, 4, 5 | Li2CO3 |  | 86.9 | 54.8 | 45.8 | 62.5 | 33.3 |
| ^[62]^ (R-LCO-MA) | 2023 | SSR | LCO | SSR-1 | 2, 3, 4, 5 | Li2CO3 |  | 86.9 | 57.5 | 45.8 | 63.4 | 33.3 |
| ^[63]^ (R-NCM800) | 2021 | SSR | NMC 111 | SSR-3 | 2, 3 |  |  | 95.2 | 34.8 | 38.8 | 56.3 | 33.3 |
| ^[63]^ (R-NCM850) | 2021 | SSR | NMC 111 | SSR-3 | 2, 3 |  |  | 95.0 | 47.6 | 32.6 | 58.4 | - |
| ^[63]^ (R-NCM900) | 2021 | SSR | NMC 111 | SSR-3 | 2, 3 |  |  | 94.9 | 33.4 | 26.3 | 51.5 | 7.3 |
| ^[64]^ (MA-1.2-800) | 2018 | SSR | NMC 111 | SSR-1 | 2, 3, 4 | Li2CO3 |  | 89.3 | 47.0 | 58.3 | 64.9 | - |
| ^[64]^ (non MA-1.2-800) | 2018 | SSR | NMC 111 | SSR-1 | 2, 3, 4 | Li2CO3 |  | 93.4 | 23.7 | 58.3 | 58.5 | - |
| ^[64]^ (MA-1.1-800) | 2018 | SSR | NMC 111 | SSR-1 | 2, 3, 4 | Li2CO3 |  | 89.6 | 23.0 | 58.3 | 56.9 | - |
| ^[64]^ (MA-1.15-800) | 2018 | SSR | NMC 111 | SSR-1 | 2, 3, 4 | Li2CO3 |  | 89.4 | 40.4 | 58.3 | 62.7 | - |
| ^[65]^ (Repaired LiCoO2) | 2021 | SSR | LCO | SSR-2 | 2, 3, 4 | LiOH |  | 79.3 | 48.9 | 45.9 | 58.0 | 33.3 |
| ^[66]^ (Relithiated) | 2024 | SSR | NMC 532 | SSR-2 | 2, 3, 4, 5 | LiOH |  | 86.3 | - | 67.9 | - | 33.3 |
| ^[67]^ (Regenerated) | 2023 | SSR | NMC 631 | SSR-1 | 2 | Li2CO3 |  | 92.7 | 61.5 | 37.7 | 64.0 |  |
| ^[68]^(LiOH-added R-LMO) | 2024 | SSR | LMO | SSR-2 |  | LiOH |  | 76.9 | - | 71.1 | - | - |
| ^[69]^ (Flash) | 2024 | SSR | LCO | SSR-1 | 2, 6 | Li2CO3 |  | 95.8 | - | 58.2 | - | - |
| ^[70]^ (RLCO) | 2024 | SSR | LCO | SSR-1 | 1, 2, 3, 4, 5,6 | Li2CO3 |  | 92.8 | 71.1 | 45.8 | 69.9 | - |
| ^[70]^ (LCO-1%) | 2024 | SSR | LCO | SSR-1 | 1, 2, 3, 4, 5,6 | Li2CO3 |  | 92.6 | 75.6 | 45.8 | 71.3 | 33.3 |
| ^[70]^ (LCO-2%) | 2024 | SSR | LCO | SSR-1 | 1, 2, 3, 4, 5,6 | Li2CO3 |  | 92.3 | 73.2 | 45.8 | 70.4 | - |
| ^[70]^ (LCO-3%) | 2024 | SSR | LCO | SSR-1 | 1, 2, 3, 4, 5,6 | Li2CO3 |  | 92.0 | 71.0 | 45.8 | 69.6 | 33.3 |
| ^[71]^ (Regenerated NCM 622) | 2021 | SSR | NMC 622 | SSR-1 | 2, 3, 4, 5 | Li2CO3 |  | 92.0 | 58.4 | 20.0 | 56.8 | 33.3 |
| ^[72]^ (RPC) | 2024 | SSR | NMC 532 | SSR-1 | 2, 4, 3 | Li2CO3 |  | 93.0 | - | 52.1 | - | 30.4 |
| ^[72]^ (RSC) | 2024 | SSR | NMC 532 | SSR-1 | 2, 4, 3 | Li2CO3 |  | 84.3 | - | 1.3 | - | 29.1 |
| ^[73]^ (4h) | 2024 | SSR | NMC 532 | SSR-3 | 1, 2, 4, 5, 6 | |  | 97,1 | 22.2 | - | - | - |
| ^[73]^ (6h) | 2024 | SSR | NMC 532 | SSR-3 | 1, 2, 4, 5, 6 | |  | 96,9 | 21.2 | - | - | - |
| ^[73]^ (8 h) | 2024 | SSR | NMC 532 | SSR-3 | 1, 2, 4, 5, 6 | |  | 96,7 | 27.3 | - | - | - |
| ^[74]^ (LNCM-800) | 2021 | SSR | NMC 532 | SSR-1 | 2, 3, 4 | Li2CO3 |  | 89.6 | - | 58.1 | - | - |
| ^[74]^ (LNCM-950) | 2021 | SSR | NMC 532 | SSR-1 | 2, 3, 4 | Li2CO3 |  | 89.0 | - | 37.6 | - | - |
| ^[74]^ (LNCM-1000) | 2021 | SSR | NMC 532 | SSR-1 | 2, 3, 4 | Li2CO3 |  | 88.7 | - | 27.0 | - | - |
| ^[75]^ (3-RNCM800) | 2024 | SSR | NMC 622 | SSR-2 | 2, 3, 4, 5 | LiOH |  | 90.2 | 32.9 | 58.4 | 60.5 | - |
| ^[75]^ (3-RNCM750) | 2024 | SSR | NMC 622 | SSR-2 | 2, 3, 4, 5 | LiOH |  | 89.6 | 60.3 | 63.6 | 71.2 | 22.4 |
| ^[75]^ (3-RNCM700) | 2024 | SSR | NMC 622 | SSR-2 | 2, 3, 4, 5 | LiOH |  | 89.2 | 30.6 | 67.9 | 62.6 | 22.4 |
| ^[76]^ (H-LCO) | 2023 | SSR | LCO | SSR-1 | 1, 2, 4, 5, 6 | Li2CO3 |  | 93.1 | 42.9 | 52.1 | 62.7 |  |
| ^[76]^ (HS-LCO) | 2023 | SSR | LCO | SSR-1 | 1, 2, 4, 5, 6 | Li2CO3 |  | 92.4 | 57.4 | 41.5 | 63.8 | 33.3 |
| ^[77]^ (RLCO) | 2024 | SSR | LCO | SSR-1 | 1, 2, 3, 4, 5, 6 | Li2CO3 |  | 93.5 | 85.6 | 52.1 | 77.1 | 33.3 |
| ^[78]^ (R-LCO) | 2024 | SSR | LCO | SSR-1 | 1, 2, 3, 4, 5, 6 | Li2CO3 |  | 88.2 | 44.8 | 45.8 | 59.6 | 33.7 |
| ^[78]^ (R-LCO-0.02A) | 2024 | SSR | LCO | SSR-1 | 1, 2, 3, 4, 5, 6 | Li2CO3 |  | 88.2 | 62.9 | 45.8 | 65.6 | 33.7 |
| ^[78]^ (R-LCO-0.05A) | 2024 | SSR | LCO | SSR-1 | 1, 2, 3, 4, 5, 6 | Li2CO3 |  | 88.1 | 59.3 | 45.8 | 64.4 |  |
| ^[78]^ (R-LCO-0.2A) | 2024 | SSR | LCO | SSR-1 | 1, 2, 3, 4, 5, 6 | Li2CO3 |  | 88.1 | 54.1 | 45.8 | 62.7 | 33.3 |
| ^[78]^ (R-LCO-0.5A) | 2024 | SSR | LCO | SSR-1 | 1, 2, 3, 4, 5, 6 | Li2CO3 |  | 88.0 | 54.5 | 45.8 | 62.8 |  |
| ^[79]^ (Regenerated) | 2023 | SSR | NMC 532 | SSR-3 | 1, 2, 3, 4, 5 | |  | 96,3 | 35.0 | - | - | 33.2 |
| ^[80]^ (R-LCO) | 2024 | SSR | LCO | SSR-3 | 1, 2, 3, 4, 5, 6 | |  | 95,3 | 62.9 | - | - | 31.9 |
| ^[81]^ (LMNCO-1) | 2022 | SSR | LCO | SSR-1 | 1, 2, 3, 4, 5, 6 | Li2CO3 |  | 93.7 | 70.1 | 45.9 | 69.9 | - |
| ^[82]^ (NLCO0.01) | 2021 | SSR | LCO | SSR-1 | 1, 2, 3, 4, 5 | Li2CO3 |  | 89.8 | 85.2 | 52.1 | 75.7 | - |
| ^[82]^ (RLCO) | 2021 | SSR | LCO | SSR-1 | 1, 2, 3, 4, 5 | Li2CO3 |  | 89.8 | 78.1 | 52.1 | 73.3 | - |
| ^[83]^ (Relithiatied) | 2022 | SSR | NMC 111 | SSR-2 | 2, 3, 4, 5 | LiOH |  | 27.9 | - | 58.7 | - | 33.3 |
| ^[84]^ (Relithiated) | 2024 | SSR | LCO | SSR-1 | 1, 2, 4, 5 | Li2CO3 |  | 89.6 | - | 64.2 | - | 34.0 |
| ^[85]^ (R-LCO-70) | 2022 | SSR | LCO | SSR-1 | 1, 2, 3, 4, 5 | Li2CO3 |  | 93.5 | 70.1 | 45.8 | 69.8 |  |
| ^[85]^ (R-LCO-40) | 2022 | SSR | LCO | SSR-1 | 1, 2, 3, 4, 5 | Li2CO3 |  | 93.5 | 86.6 | 45.8 | 75.3 | 32.7 |
| ^[85]^ (R-LCO-15) | 2022 | SSR | LCO | SSR-1 | 1, 2, 3, 4, 5 | Li2CO3 |  | 93.5 | 51.9 | 45.8 | 63.8 | - |
| ^[86]^ (DLCO) | 2021 | SSR | LCO | SSR-1 | 1, 2, 3, 4, 5, 6 | Li2CO3 |  | 86.7 | 24.5 | 27.2 | 46.1 | - |
| ^[86]^ (MTLCO-11) | 2021 | SSR | LCO | SSR-1 | 1, 2, 3, 4, 5, 6 | Li2CO3 |  | 86.7 | 32.3 | 27.2 | 48.7 | 33.3 |
| ^[86]^ (MTLCO-22) | 2021 | SSR | LCO | SSR-1 | 1, 2, 3, 4, 5, 6 | Li2CO3 |  | 86.6 | 35.7 | 27.2 | 49.8 | 33.3 |
| ^[86]^ (MTLCO-24) | 2021 | SSR | LCO | SSR-1 | 1, 2, 3, 4, 5, 6 | Li2CO3 |  | 86.5 | 54.1 | 27.2 | 55.9 | 33.3 |
| ^[86]^ (MTLCO-42) | 2021 | SSR | LCO | SSR-1 | 1, 2, 3, 4, 5, 6 | Li2CO3 |  | 86.5 | 61.1 | 27.2 | 58.3 | - |
| ^[87]^ (3h-800-1) | 2024 | SSR | NCM 831205 | SSR-1 | 1, 2, 3, 4, 5 | Li2CO3 |  | 83.2 | 37.4 | 38.8 | 53.1 | 31.4 |
| ^[87]^ (3h-800-1,06) | 2024 | SSR | NCM 831205 | SSR-1 | 1, 2, 3, 4, 5 | Li2CO3 |  | 82.8 | 5.6 | 38.8 | 42.4 | 29.6 |
| ^[87]^ (6h-800-1,03-n/w) | 2024 | SSR | NCM 831205 | SSR-1 | 1, 2, 3, 4, 5 | Li2CO3 |  | 83.7 | 28.8 | 38.8 | 50.5 | 27.6 |
| ^[87]^ (3h-800-1,03-w) | 2024 | SSR | NCM 831205 | SSR-1 | 1, 2, 3, 4, 5 | Li2CO3 |  | 82.9 | 22.5 | 38.8 | 48.1 | 28.5 |
| ^[87]^ (6h-800-1,03-w) | 2024 | SSR | NCM 831205 | SSR-1 | 1, 2, 3, 4, 5 | Li2CO3 |  | 82.6 | 25.6 | 38.8 | 49.0 | - |
| ^[87]^ (10h-800-1,03-w) | 2024 | SSR | NCM 831205 | SSR-1 | 1, 2, 3, 4, 5 | Li2CO3 |  | 82.1 | 14.4 | 38.8 | 45.1 | 0.1 |
| ^[87]^ (3h-800-1,03) | 2024 | SSR | NCM 831205 | SSR-1 | 1, 2, 3, 4, 5 | Li2CO3 |  | 83.0 | 18.4 | 38.8 | 46.7 | 0.1 |
| ^[87]^ (3h-750-1,03) | 2024 | SSR | NCM 831205 | SSR-1 | 1, 2, 3, 4, 5 | Li2CO3 |  | 82.7 | 18.2 | 44.0 | 48.3 | 0.1 |
| ^[88]^ (R-LCO) | 2024 | SSR | LCO | SSR-1 | 1, 2, 3, 4, 5, 6 | Li2CO3 |  | 88.6 | 41.3 | 28.5 | 52.8 | 0.1 |
| ^[88]^ (R-LCO-0.2F) | 2024 | SSR | LCO | SSR-1 | 1, 2, 3, 4, 5, 6 | Li2CO3 |  | 88.5 | 55.1 | 28.5 | 57.4 | 0.1 |
| ^[88]^ (R-LCO-0.5F) | 2024 | SSR | LCO | SSR-1 | 1, 2, 3, 4, 5, 6 | Li2CO3 |  | 88.3 | 49.9 | 28.5 | 55.6 | 0.1 |
| ^[88]^ (R-LCO-1F) | 2024 | SSR | LCO | SSR-1 | 1, 2, 3, 4, 5, 6 | Li2CO3 |  | 88.1 | 31.4 | 28.5 | 49.3 | 0.1 |
| ^[89]^ (R-NCMA0) | 2023 | SSR | NMC 111 | SSR-3 | 1, 2, 3, 4, 5, 6 | |  | 91,2 | 4.5 | - | - | 22.8 |
| ^[89]^ (R-NCMA0.2) | 2023 | SSR | NMC 111 | SSR-3 | 1, 2, 3, 4, 5, 6 | |  | 91,2 | 10.7 | - | - | 22.9 |
| ^[89]^ (R-NCMA0.5) | 2023 | SSR | NMC 111 | SSR-3 | 1, 2, 3, 4, 5, 6 | |  | 91,2 | 9.6 | - | - | 23.1 |
| ^[89]^ (R-NCMA1) | 2023 | SSR | NMC 111 | SSR-3 | 1, 2, 3, 4, 5, 6 | |  | 91,2 | 5.3 | - | - | 23.4 |
| ^[90]^ (0.5 %Sm@OR) | 2023 | SSR | NMC 622 | SSR-3 | 1, 2, 3, 4, 5, 6 | |  | 91,3 | 53.2 | - | - | - |
| ^[90]^ (1.0 %Sm@OR) | 2023 | SSR | NMC 622 | SSR-3 | 1, 2, 3, 4, 5, 6 | |  | 90,5 | 43.9 | - | - | - |
| ^[90]^ (2.0 %Sm@OR) | 2023 | SSR | NMC 622 | SSR-3 | 1, 2, 3, 4, 5, 6 | |  | 88,8 | 45.7 | - | - | - |
| ^[91]^ (RNCM) | 2020 | SSR | NMC 532 | SSR-2 | 1, 2, 3, 4, 5, 6 | LiOH |  | 84.5 | 77.9 | 34.9 | 65.8 | - |
| ^[91]^ (RNCMP-1) | 2020 | SSR | NMC 532 | SSR-2 | 1, 2, 3, 4, 5, 6 | LiOH |  | 84.0 | 80.6 | 34.9 | 66.5 | 0.8 |
| ^[91]^ (RNCMP-2) | 2020 | SSR | NMC 532 | SSR-2 | 1, 2, 3, 4, 5, 6 | LiOH |  | 83.9 | 85.1 | 34.9 | 68.0 | 1.6 |
| ^[91]^ (RNCMP-3) | 2020 | SSR | NMC 532 | SSR-2 | 1, 2, 3, 4, 5, 6 | LiOH |  | 83.7 | 82.4 | 34.9 | 67.0 | 3.0 |
| ^[92]^ (R-LCO) | 2021 | SSR | LCO | SSR-1 | 1, 2, 3, 4, 5, 6 | Li2CO3 |  | 89.5 | 63.3 | 45.8 | 66.2 | 18.8 |
| ^[92]^ (1%-LMCO) | 2021 | SSR | LCO | SSR-1 | 1, 2, 3, 4, 5, 6 | Li2CO3 |  | 89.4 | 68.5 | 45.8 | 67.9 | 19.5 |
| ^[92]^ (3%-LMCO) | 2021 | SSR | LCO | SSR-1 | 1, 2, 3, 4, 5, 6 | Li2CO3 |  | 89.3 | 71.2 | 45.8 | 68.8 | 20.2 |
| ^[92]^ (5%-LMCO) | 2021 | SSR | LCO | SSR-1 | 1, 2, 3, 4, 5, 6 | Li2CO3 |  | 89.1 | 62.7 | 45.8 | 65.9 | 21.8 |
| ^[93]^ (NCM-R) | 2024 | SSR | NMC 532 | SSR-1 | 1, 2, 3, 4, 5, 6 | Li2CO3 |  | 82.7 | 62.4 | 38.8 | 61.3 | 33.3 |
| ^[94]^ (MTLCO-11) | 2022 | SSR | LCO | SSR-1 | 1, 2, 3, 4, 5, 6 | Li2CO3 |  | 86.4 | - | 27.2 | - | 29.0 |
| ^[94]^ (CLCO-0,1) | 2022 | SSR | LCO | SSR-1 | 1, 2, 3, 4, 5, 6 | Li2CO3 |  | 87.5 | - | 71.4 | - | 26.7 |
| ^[94]^ (CLCO-0,25) | 2022 | SSR | LCO | SSR-1 | 1, 2, 3, 4, 5, 6 | Li2CO3 |  | 87.5 | - | 71.4 | - |  |
| ^[94]^ (CLCO-0,5) | 2022 | SSR | LCO | SSR-1 | 1, 2, 3, 4, 5, 6 | Li2CO3 |  | 87.5 | - | 71.4 | - | 33.3 |
| ^[95]^ (Calcined) | 2020 | SSR | LMO | SSR-1 | 1, 2, 3, 4, 5, 6 | Li2CO3 |  | 94.8 | - | - | - | 31.3 |
| ^[96]^ (R-NCM) | 2022 | SSR | NMC 532 | SSR-1 | 1, 2, 3, 4, 5, 6 | Li2CO3 |  | 92.6 | 72.4 | 37.7 | - | 31.0 |
| ^[97]^ (R-LCO-1173) | 2023 | SSR | LCO | SSR-3 | 1, 2, 3, 4, 5 | |  | 83,7 | - | 45.9 | - | - |
| ^[97]^ (R-LCO-Li-1173) | 2023 | SSR | LCO | SSR-1 | 1, 2, 3, 4, 5 | Li2CO3 |  | 81.0 | - | 45.7 | - | 22.3 |
| ^[97]^ (R-LCO-1440) | 2023 | SSR | LCO | SSR-3 | 1, 2, 3, 4, 5 | |  | 82,7 | 44.9 | 9.6 | 45.8 |  |
| ^[97]^ (R-LCO-Li-1440) | 2023 | SSR | LCO | SSR-1 | 1, 2, 3, 4, 5 | Li2CO3 |  | 80.0 | 61.3 | 9.3 | 50.2 | 33.3 |
| ^[97]^ (R-LCO-Li-1300) | 2023 | SSR | LCO | SSR-1 | 1, 2, 3, 4, 5 | Li2CO3 |  | 80.3 | 46.8 | 20.3 | 49.1 | - |
| ^[97]^ (R-LCO-Li-1390) | 2023 | SSR | LCO | SSR-1 | 1, 2, 3, 4, 5 | Li2CO3 |  | 80.0 | 45.5 | 9.3 | 44.9 | - |
| ^[97]^ (R-LCO-Li-1560) | 2023 | SSR | LCO | SSR-1 | 1, 2, 3, 4, 5 | Li2CO3 |  | 79.8 | 51.1 | 0.0 | 43.6 | 23.1 |
| ^[97]^ (R-LCO-Li-1700) | 2023 | SSR | LCO | SSR-1 | 1, 2, 3, 4, 5 | Li2CO3 |  | 79.8 | 38.0 | 0.0 | 39.3 | - |
| ^[98]^ (R-LCO) | 2024 | SSR | LCO | SSR-3 | 1, 2, 3, 4, 5, 6 | |  | - | - | - | - | 23.1 |
| ^[98]^ (R-LCO-0.4Mg) | 2024 | SSR | LCO | SSR-1 | 1, 2, 3, 4, 5, 6 | Li2CO3 |  | 94.4 | 44.6 | 45.8 | 61.6 | 23.1 |
| ^[98]^ (R-LCO-0.4Mg-0,17P) | 2024 | SSR | LCO | SSR-1 | 1, 2, 3, 4, 5, 6 | Li2CO3 |  | 93.1 | 64.4 | 45.8 | 67.8 | 23.1 |
| ^[99]^ (D-NCM622-R-H) | 2024 | SSR | NMC 622 | SSR-3 | 1, 2, 3, 4, 5 | |  | 90,0 | 78.6 | 58.2 | 75.6 | 23.1 |
| ^[100]^(R-NCM83) | 2024 | SSR | NCM 83 | SSR-3 | 1, 2, 3, 4, 5, 6 | |  | 73,6 | 99.0 | 64.5 | 79.0 | 32.3 |
| ^[101]^ (Regenerated LCO) | 2025 | EC | LCO |  | 1, 2, 3 |  | yes | 79.2 | 41.0 | 73.6 | 64.6 | - |
| ^[102]^ (EC-RNCM-2) | 2025 | EC | NMC 532 |  | 1, 2, 3, 4, 5, 6 | | yes | 77,8 | 56.0 | 64.6 | 66.1 | - |
| ^[103]^ (Relithiated) | 2020 | EC | NMC 442 |  | 1, 2, 3 |  | no | 79.2 | 3.4 | 98.4 | 60.3 | - |
| ^[104]^ (Recovered) | 2025 | EC | LCO |  | 1, 2, 3, 4, 5, 6 | | no | 81,6 | 8.6 | 67.8 | 52.7 | - |
| ^[105]^ (R3) | 2025 | Chem | NMC 622 |  | 1, 2, 3 |  | yes | 71.9 | 49.6 | 72.3 | 64.6 | - |
| ^[106]^ (RLCO -60) | 2025 | Chem | LCO |  | 2,3,4 |  | yes | 87.2 | 70.0 | 67.5 | 74.9 | - |
| ^[106]^ (RLCO-80) | 2025 | Chem | LCO |  | 2,3,4 |  | yes | 87.1 | 71.0 | 67.4 | 75.2 | - |
| ^[106]^ (RLCO-100) | 2025 | Chem | LCO |  | 2,3,4 |  | yes | 87.1 | 67.2 | 67.3 | 73.9 | - |
| ^[107]^ (r-LMO-Ni) | 2025 | Chem | LMO |  |  |  | yes | 73.8 | - | 50.8 | - | - |
| ^[107]^ (r-LMO-Mn) | 2025 | Chem | LMO |  |  |  | yes | 74.1 | - | 50.8 | - | - |
| ^[107]^ (r-LMO-Li) | 2025 | Chem | LMO |  |  |  | yes | 75.7 | - | 50.9 | - | - |
| ^[108]^ (D-NCM 30 min) | 2025 | Chem | NMC 622 |  | 1,2,3 |  | no | 62.7 | - | 99.7 | - | - |
| ^[108]^ (D-NCM 1h) | 2025 | Chem | NMC 622 |  | 1,2,3 |  | no | 62.7 | - | 99.7 | - | - |
| ^[108]^ (D-NCM 3h) | 2025 | Chem | NMC 622 |  | 1,2,3 |  | no | 62.5 | - | 99.7 | - | - |
| ^[108]^ (D-NCM 6h) | 2025 | Chem | NMC 622 |  | 1,2,3 |  | no | 62.2 | - | 99.7 | - | - |
| ^[109]^ (R-LCO) | 2025 | MST | LCO | MST-2 | 1, 2, 3, 4, 5, 6 | | yes | 72,4 | 73.4 | 64.8 | 70.2 | - |
| ^[110]^ (R-NCM) | 2025 | MST | NMC 532 | MST-1 | 1, 2, 3, 4, 5 | | yes | 85,2 | 43.7 | 57.1 | 62.0 | - |
| ^[110]^ (Nb-R-NCM) | 2025 | MST | NMC 532 | MST-1 | 1, 2, 3, 4, 5 | | yes | 84,0 | 62.7 | 57.1 | 67.9 | - |
| ^[111]^ (3MS-NCM523) | 2025 | MST | NMC 532 | MST-1 | 1, 2, 3, 4, 5 | | yes | 36,3 | 59.9 | 57.1 | 51.1 | - |
| ^[111]^ (3MT-NCM523) | 2025 | MST | NMC 532 | MST-1 | 1, 2, 3, 4, 5, 6 | | yes | 29,1 | 69.5 | 25.0 | 41.2 | - |
| ^[112]^ (R-NCM622) | 2025 | MST | NMC 622 | MST-1 | 1, 2, 3, 4, 5, 6 | | yes | 80,5 | 73.8 | 28.9 | 61.1 | - |
| ^[113]^ (R-NCM622-850) | 2025 | MST | NMC 622 | MST-1 | 2,3,4 |  | yes | 79.1 | 33.7 | 57.1 | 56.6 | - |
| ^[113]^ (R-NCM622-900) | 2025 | MST | NMC 622 | MST-1 | 2,3,4 |  | yes | 79.0 | 44.6 | 55.2 | 59.6 | - |
| ^[113]^ (R-NCM622-950) | 2025 | MST | NMC 622 | MST-1 | 2,3,4 |  | yes | 79.0 | 39.4 | 53.2 | 57.2 | - |
| ^[114]^ (CONVMS) | 2025 | MST | NMC 111 | MST-1 | 2, 3, 5 |  | yes | 89.7 | 41.6 | 55.2 | 62.2 | - |
| ^[114]^ (RNMC20) | 2025 | MST | NMC 111 | MST-1 | 2, 3, 5 |  | yes | 82.1 | 20.5 | 100.0 | 67.5 | - |
| ^[114]^ (RNMC20‑350‑2h‑SA) | 2025 | MST | NMC 111 | MST-1 | 2, 3, 5 |  | yes | 79.4 | 34.3 | 55.2 | 56.3 | - |
| ^[115]^ (R-NCM) | 2025 | MST | NMC 111 | MST-1 | 1, 2, 3, 5 |  | yes | 92.3 | 68.1 | 55.1 | 71.8 | - |
| ^[116]^ (R-NCM) | 2025 | MST | NMC 532 | MST-1 | 1,2,3,4,5 |  | yes | 70.7 | 63.7 | 33.8 | 56.1 | - |
| ^[117]^ (R-NCM622-F) | 2025 | MST | NMC 622 | MST-1 | 1, 2, 3, 4, 5, 6 | | yes | 46,0 | 80.9 | 55.2 | 60.7 | - |
| ^[117]^ (R-NCM622) | 2025 | MST | NMC 622 | MST-1 | 1, 2, 3, 4, 5, 6 | | yes | 46,0 | 55.0 | 55.2 | 52.0 | - |
| ^[118]^ (REBNCM) | 2025 | MST | NMC 532 | MST-2 | 2,3,4,5 |  | yes | 74.7 | 55.8 | 67.8 | 66.1 | - |
| ^[118]^ (RENCM) | 2025 | MST | NMC 532 | MST-2 | 2,3,4,5 |  | yes | 55.8 | 60.5 | 66.4 | 60.9 | - |
| ^[119]^ (RSNCM) | 2025 | MST | NMC 532 | MST-1 | 2,3,4,5,6 |  | yes | 55.5 | 30.3 | 55.5 | 47.1 | - |
| ^[119]^ (LPDR-SNCM) | 2025 | MST | NMC 532 | MST-1 | 2,3,4,5,6 |  | yes | 55.1 | 57.3 | 55.5 | 56.0 | - |
| ^[120]^ (R-NCM811) | 2025 | MST | NMC 811 | MST-2 | 2,3,4 |  | yes | 28.3 | 79.1 | 52.3 | 53.2 | - |
| ^[121]^ (R-NCM) | 2025 | MST | NMC 532 | MST-1 | 2,3,4 |  | yes | 88.5 | 70.7 | 65.9 | 75.1 | - |
| ^[122]^ (R-NCM) | 2025 | MST | NMC 811 | MST-3 | 1,2,3,4,5,6 | | yes | 59,7 | 71.6 | 48.2 | 59.9 | - |
| ^[123]^ (RLCO) | 2025 | MST | LCO | MST-2 | 1,2,3,4,5 |  | no | 89.1 | 68.5 | 93.3 | 83.6 | - |
| ^[124]^ (R-NCM) | 2025 | MST | NMC 811 | MST-3 | 2, 3, 4, 5, 6 | | yes | 89,9 | 58.6 | 37.7 | 62.1 | - |
| ^[125]^ (H-LCO) | 2025 | Hydro | LCO | Hydro-2 | 1,2,3,4,6 |  | yes | 85.4 | 69.3 | 58.9 | 71.2 | - |
| ^[125]^ (R-LCO) | 2025 | Hydro | LCO | Hydro-2 | 1,2,3,4,6 |  | yes | 85.1 | 78.6 | 58.9 | 74.2 | - |
| ^[126]^ (LPO-NCM622) | 2025 | Hydro | NMC 622 | Hydro-2 | 1,2,3,6 |  | yes | 89.0 | 71.3 | 67.6 | 76.0 | - |
| ^[127]^ (H-NCM523) | 2025 | Hydro | NMC 532 | Hydro-2 | 2, 3, 4, 6 |  | yes | 92.2 | 28.2 | 98.8 | 73.1 | - |
| ^[127]^ (GHCR-NCM523) | 2025 | Hydro | NMC 532 | Hydro-2 | 2, 3, 4, 6 |  | yes | 89.3 | 30.3 | 68.7 | 62.8 | - |
| ^[128]^ (rCBMup_1%B) | 2025 | Hydro | NCM 89 | Hydro-3 | 2, 3, 4, 6 |  | yes | 83.5 | 86.2 | 67.8 | 79.2 | - |
| ^[129]^ (regenerated) | 2025 | SSR | LMO/NMC 111 | | 2,3 | LiNO3 |  | 52,0 | 68.3 | 89.2 | 69.8 | - |
| ^[130]^ (Recycled) | 2025 | SSR | NMC 622 | SSR-2 | 2, 3 | LiOH |  | 88.0 | 0.0 | 45.5 | 44.5 | - |
| ^[131]^ (R-LCO) | 2025 | SSR | LCO |  | 2, 4, 5, 6 | LiNO3 |  | 91.7 | 43.9 | 62.1 | 65.9 | - |
| ^[131]^ (R-LCO@LLTO/LT) | 2025 | SSR | LCO |  | 2, 4, 5, 6 | LiNO3 |  | 85.7 | 57.6 | 61.9 | 68.4 | - |
| ^[66]^ (relithiated) | 2025 | SSR | NMC 532 | SSR-2 | 2, 3, 4 | LiOH |  | 86.9 | - | 73.7 | - | - |
| ^[132]^ (RLCO) | 2025 | SSR | LCO | SSR-2 | 1, 2, 3, 4, 5, | LiOH |  | 86.6 | 53.7 | 67.8 | 69.4 | - |
| ^[132]^ (RLCO-0,5A) | 2025 | SSR | LCO | SSR-2 | 1, 2, 3, 4, 5, | LiOH |  | 86.6 | 48.2 | 67.8 | 67.5 | - |
| ^[132]^ (RLCO-0,5A@LAO) | 2025 | SSR | LCO | SSR-2 | 1, 2, 3, 4, 5, | LiOH |  | 84.4 | 60.0 | 35.7 | 60.0 | - |
| ^[133]^ (R-45-80) | 2025 | SSR | NMC 811 | SSR-2 | 1,2,3,4,5,6 | LiOH |  | 90.2 | 61.8 | 15.4 | 55.8 | - |
| ^[133]^ (R-45-60) | 2025 | SSR | NMC 811 | SSR-2 | 1,2,3,4,5,6 | LiOH |  | 90.2 | 63.1 | 15.4 | 56.2 | - |
| ^[134]^ (RNCM) | 2025 | SSR | NCM 83 | SSR-2 | 2, 3, 6 | LiOH |  | 86.0 | 58.5 | 54.4 | 66.3 | - |
| ^[93]^ (NCM-R) | 2025 | SSR | NMC 532 | SSR-2 | 2, 3, 5, 6 | LiOH |  | 82.7 | 68.1 | 69.9 | 73.6 | - |
| ^[135]^ (DRL1) | 2025 | SSR | LCO | SSR-3 | 1, 2, 3, 4, 5, 6 | |  | 90,1 | 50.9 | 43.6 | 61.5 | - |
| ^[135]^ (DRL2) | 2025 | SSR | LCO | SSR-3 | 1, 2, 3, 4, 5, 6 | |  | 89,3 | 29.5 | 43.6 | 54.1 | - |
| ^[135]^ (DRL3) | 2025 | SSR | LCO | SSR-3 | 1, 2, 3, 4, 5, 6 | |  | 88,5 | 12.7 | 43.6 | 48.3 | - |
| ^[135]^ (DRL4) | 2025 | SSR | LCO | SSR-3 | 1, 2, 3, 4, 5, 6 | |  | 87,8 | 12.1 | 43.6 | 47.8 | - |
| ^[136]^ (R-NCM622) | 2025 | SSR | NMC 622 | SSR-2 | 2, 3, 4, 5, 6 | LiOH |  | 88.7 | 82.9 | 41.7 | 71.1 | - |
| ^[137]^ (R-NCM) | 2025 | SSR | NCM 82 | SSR-2 | 1,2,3,4,5,6 | LiOH |  | 89.1 | 91.7 | 71.8 | 84.2 | - |
| ^[137]^ (R-NCM-HS) | 2025 | SSR | NCM 82 | SSR-2 | 1,2,3,4,5,6 | LiOH |  | 88.2 | 95.4 | 71.8 | 85.1 | - |
| ^[138]^ (D-RNCM) | 2025 | SSR | NMC 532 | SSR-2 | 1,2,3,4,5 | LiOH |  | 90.9 | 30.6 | 66.0 | 62.5 | - |
| ^[139]^ (N-LCO) | 2025 | SSR | LCO | SSR-1 | 1,2,4,5,6 | Li2CO3 |  | 87.5 | 66.6 | 61.6 | 71.9 | - |
| ^[139]^ (U-LCO) | 2025 | SSR | LCO | SSR-1 | 1,2,4,5,6 | Li2CO3 |  | 86.7 | 77.4 | 61.6 | 75.3 | - |
| ^[140]^ (RLMO-NCM) | 2025 | SSR | NMC 532 | SSR-2 | 2, 3, 4, 5, 6 | LiOH |  | 71.7 | 67.3 | 54.0 | 64.3 | - |
| ^[141]^ (RNMC 1.05:1) | 2025 | SSR | NMC 111 | SSR-1 | 2, 3, 4 | Li2CO3 |  | 88.4 | 70.6 | 69.7 | 76.2 | - |
| ^[141]^ (RNMC 1.1:1) | 2025 | SSR | NMC 111 | SSR-1 | 2, 3, 4 | Li2CO3 |  | 88.3 | 81.5 | 69.7 | 79.8 | - |
| ^[141]^ (RNMC 1.15:1) | 2025 | SSR | NMC 111 | SSR-1 | 2, 3, 4 | Li2CO3 |  | 88.1 | 73.5 | 69.7 | 77.1 | - |
| ^[141]^ (RNMC 1.2:1) | 2025 | SSR | NMC 111 | SSR-1 | 2, 3, 4 | Li2CO3 |  | 88.0 | 63.7 | 69.6 | 73.8 | - |
| ^[141]^ (RNMC 700) | 2025 | SSR | NMC 111 | SSR-1 | 2, 3, 4 | Li2CO3 |  | 88.4 | 64.5 | 73.5 | 75.5 | - |
| ^[141]^ (RNMC 750) | 2025 | SSR | NMC 111 | SSR-1 | 2, 3, 4 | Li2CO3 |  | 88.3 | 71.9 | 71.6 | 77.3 | - |
| ^[141]^ (RNMC 800) | 2025 | SSR | NMC 111 | SSR-1 | 2, 3, 4 | Li2CO3 |  | 88.3 | 81.4 | 69.7 | 79.8 | - |
| ^[141]^ (RNMC 850) | 2025 | SSR | NMC 111 | SSR-1 | 2, 3, 4 | Li2CO3 |  | 88.2 | 76.0 | 67.7 | 77.3 | - |
| ^[142]^ (R-NCM-800) | 2025 | SSR | NMC 622 | SSR-1 | 2,3,4 | Li2CO3 |  | 90.6 | 68.9 | 51.1 | 70.2 | - |
| ^[143]^ (R-750-12h) | 2025 | SSR | LCO | SSR-1 | 2, 4, 5, 6 | Li2CO3 |  | 88.3 | 48.6 | 71.7 | 69.5 | - |
| ^[143]^ (R-750-15h) | 2025 | SSR | LCO | SSR-1 | 2, 4, 5, 6 | Li2CO3 |  | 88.6 | 47.3 | 71.7 | 69.2 | - |
| ^[143]^ (R-850-12h) | 2025 | SSR | LCO | SSR-1 | 2, 4, 5, 6 | Li2CO3 |  | 88.2 | 66.3 | 67.8 | 74.1 | - |
| ^[143]^ (R-850-15h) | 2025 | SSR | LCO | SSR-1 | 2, 4, 5, 6 | Li2CO3 |  | 88.5 | 68.3 | 67.8 | 74.9 | - |
| ^[143]^ (R-950-12h) | 2025 | SSR | LCO | SSR-1 | 2, 4, 5, 6 | Li2CO3 |  | 88.1 | 60.5 | 64.0 | 70.9 | - |
| ^[143]^ (R-950-15h) | 2025 | SSR | LCO | SSR-1 | 2, 4, 5, 6 | Li2CO3 |  | 88.4 | 67.6 | 64.0 | 73.3 | - |
| ^[144]^ (R-C-NCM) | 2025 | SSR | NMC 532 | SSR-1 | 1, 2, 4, 6 | Li2CO3 |  | 93.9 | 52.5 | 67.8 | 71.4 | - |
| ^[145]^ (5LiRECAT) | 2025 | SSR | LMO | SSR-1 | 1,2,3 | Li2CO3 |  | 91.8 | 15.1 | 71.7 | 59.5 | - |
| ^[145]^ (10LiRECAT) | 2025 | SSR | LMO | SSR-1 | 1,2,3 | Li2CO3 |  | 91.7 | 16.6 | 71.7 | 60.0 | - |
| ^[145]^ (15LiRECAT) | 2025 | SSR | LMO | SSR-1 | 1,2,3 | Li2CO3 |  | 91.5 | 17.2 | 71.7 | 60.1 | - |

**Table S2.** Full technical and economic parameters for grouped relithiation methods

| **CAMs** | **Reagents and method variations** | **Process conditions** | | ***C_M_*, $∙kg^−^¹, Median,**  ***S_M_*, pts** | ***E*, kJ∙g^−^¹, Median**  ***S_Е_*, pts** | ***S_Group I_*, pts**  **Median** | **Ref.** |  |
| --- | --- | --- | --- | --- | --- | --- | --- | --- |
| **Solid-state reaction (SSR)** | | | | | | | | |
| LCO, NMC 151570, NMC 111, NMC 631, NMC 622, NMC 532, NCM 831205, LMO | Li_2_CO_3_  (SSR-1) | 500–1000 °C (typically 700–900 °C), 0.1–30 h (commonly 8.5–15 h), air (most common) or inert (e.g., Ar for pre-treatment), ball milling (2:1 ball ratio, 250–500 rpm, 3–4 h) or mortar mixing (agate). | | 25.8–128.8 $∙kg^−^¹,  37.1 $∙kg^−^¹,  87.1–97.4 pts | 295.1-1148.1 kJ·g⁻¹, 560.8 kJ·g⁻¹,  0–70.5 pts | 79.8–95.8 pts  89.3 pts | ^[61,62,64,65,67,69–78,86,92,95–98,146–148]^ |  |
| NMC532, LCO, LMO, NMC622, NMC111 | LiOH  (SSR-2) | 450–1000 °C (typically 750–900 °C), 5–30 h (commonly 8–15 h), air or O_2_ (most common) or inert (e.g., N₂ for LiCoO_2_), ball milling (0.1 mm ZrO_2_ balls, 2000–3000 rpm, 3–6 h) or mortar mixing (agate, about 20 min). | | 55.8–1844.8 $∙kg^−^¹,  65.8 $∙kg^−^¹,  0,0–94,4 pts | 295.1–671.8 kJ·g⁻¹, 432.1 kJ·g⁻¹,  32.8–70.5 pts | 27.9–92.2 pts  86.3 pts | ^[60,61,65,66,68,75,83,91]^ |  |
| NMC532, LCO, LMO, NMC622, NCM83 | Biphenyl-Li, amyloxyllithium, surface contaminants, CB and PVDF residuals  (SSR-3) | 450–1000 °C (typically 700–900 °C), 0.1–30 h (commonly 8–15 h), air (most common) or O₂/N₂/Ar (e.g., N₂ for LCO), ball milling (0.1 mm ZrO₂ balls, 2000–3000 rpm, 3–6 h) or mortar mixing (agate, 20–30 min). | | 0.0–189.5 $∙kg^−^¹, 2.1 $∙kg^−^¹,  81,1–100.0 pts | 255.3–935.6 kJ·g⁻¹,  527.6 kJ·g⁻¹,  6.4–74.5 pts | 73.6–96.9 pts  91.0 pts | ^[73,75,79,89,90,97,99,100]^ |  |
| **Molten salt thermochemistry (MST)** | | | | | | | | |
| NCM 532 | Inorganic lithium compounds: LiOH, LiNO_3­­_, Li_2_CO_3_, LiI  (MST-1) | 200–950 °C (typically 300–320 °C + 800–850 °C), 2–12 h (commonly 4–10 h per step), air or O_2_ (air most common), ball milling (agate or ZrO_2_, 10–30 min to 3 h) or mortar mixing (agate, 10–30 min), molten salt (eutectic LiNO_3_/LiOH = 3:2 or LiOH/Li_2_CO_3_ = ≈0.84:0.16, LiOH/Li_2_CO_3_/LiNO_3_ = 2:1:1, or LiI/LiOH = 55:45), Li-excess (typically 1.05–1.1 Li:M), additives optional (e.g., 3–5 wt% Co_2_O_3_ or MnO_2_, 1–6 wt% Al, 3 mol% TiO_2_), water washing (3–5× DI water, 20–25 °C, 1–3 h) and drying (60–120 °C, air or vacuum). | | 35.3–5956.3$∙kg^−^¹, 91.2 $∙kg^−^¹,  0,0–96,5 pts | 228.8–611.4 kJ·g⁻¹,  457.7 kJ·g⁻¹,  38.9–77.1 pts | 28.1–92.3 pts  87.8 pts | ^[42,45,47,52,55,57,58]^ |  |
| NMC 111, NCM 532, LCO | Organic: ionic liquid ([C_2_OHmim]), CH_3_COOLi, lithium salicylate, urea, betaine, LiCl  (MST-2) | 40–850 °C (typically 150–300 °C + 500–850 °C), 4–24 h (commonly 6–10 h), air or O₂ (air most common), mortar mixing or magnetic stirring (10–30 min), ionic liquids (e.g., [C₂mim][NTf₂], 10–50 mL per 25 g, reused up to 89%), eutectic or DES systems (e.g., LiOH:LiNO₃:LiSA = 2:3:5; LiNO_3_:LiOH:CCL = 9:6:10; betaine:ethylene glycol lithium:urea = 1.4:2.5:0.2), Li-excess optional (typically 5 wt% Li₂CO₃ added before sintering), washing with acetone/ethanol/water (3×), drying at 70–120 °C (1–6 h), sintering (optional, 500–850 °C for 4–6 h). | | 381.3–  4661.5  $∙kg^−^¹, 422.2 $∙kg^−^¹,  0.0–61.8 pts | 0.9–728.0 kJ·g⁻¹,  105.2 kJ·g⁻¹,  0–99.9 pts | 23.2–84.0 pts  65.1 pts | ^[43,46,48,49,56]^ |  |
| NCM 532, NMC 622, LCO, NCA | Inorganic lithium and non-lithium compounds: KCl, KNO_3_, NaCl, KOH, NaOH, NaNO_3_, Na_2_SO_4_  (MST-3) | 400–900 °C (typically 700–850 °C), 2–20 h (commonly 5–12 h), air or O₂ (most common), mortar mixing (agate, 5–10 min) or hand grinding, eutectic/molten salt media (e.g., LiOH–NaCl, KCl–KNO_3_–LiNO_3_, LiOH–KOH–Li_2_CO_3_, LiCl–NaNO_3_–NaOH–NaCl, LiOH–Na_2_SO_4_), Li-excess optional (commonly 4–5 wt% LiOH or Li_2_CO_3_), washing with warm deionized water (3–6×), ultrasonication (10–30 min), drying at 60–120 °C (1–12 h), post-annealing (700–850 °C, 2–10 h, O₂). | | 428.6–11106.2 $∙kg^−^¹, 1037.2 $∙kg^−^¹,  0.0–57.1 pts | 740.1–1616.0 kJ·g⁻¹,  1285.1 kJ·g⁻¹,  0–26.0 pts | 21.7–56.3 pts  36.8 pts | ^[50,51,53,54,149]^ |  |
| **Hydrothermal (Hydro)** | | | | | | | | |
| NMC111, NMC151570, NMC553015, LCO, NMC532, LMO, LiNi_0.5_Mn_1.5_O_4_ | LiOH-based aqueous solution treatment  (Hydro-1) | 180–220 °C (commonly 220 °C), 1–6 h (typically 2–4 h), 4 M LiOH (0.1–4.0 M range), Teflon-lined or stainless-steel autoclave, stirring optional (commonly 300–500 rpm), solid–liquid ratio ≈1:20–1:80 (e.g., 1–5 g CAM in 60–125 mL solution), cooling natural or water-assisted, washing with deionized water (3–6×, pH ∼7), drying at 80–150 °C (typically overnight), grinding (manual or ball-mill), optional ultrasonication (10–30 min), post-annealing at 800–850 °C (3–6 h, O₂ or air), Li-excess (commonly 5 mol% Li_2_CO_3_ or LiOH), heating rate 5 °C min⁻¹. | | 3.0–124.6 $∙kg^−^¹, 58.9 $∙kg^−^¹,  87.5–99.7 pts | 225.6 – 853.3 kJ·g⁻¹,  509.3 kJ·g⁻¹,  14.7–77.4 pts | 80.2–92.3 pts  88.6 pts | ^[19,20,33,35,38,39,21,22,26–29,31,32]^ |  |
| LCO, NMC111, NCM532 | LiOH-based aqueous solution treatment with optional additives  (Hydro-2) | 125–220 °C (commonly 180–220 °C), 1–20 h (typically 2–6 h), 4 M LiOH (0.1–4.0 M range, sometimes mixed with H_2_O_2_, (NH_4_)_2_S_2_O_8_, KOH or Li_2_SO_4_), Teflon-lined or stainless-steel autoclave, stirring optional (commonly 300–500 rpm), solid–liquid ratio ≈1:20–1:80 (e.g., 1–5 g CAM in 60–125 mL solution), optional additives: ethanol, H_2_O_2_, ethylene glycol, Al powder/scraps, urea, ammonium persulfate, Ni/Mn acetates, LiAlTi(PO_4_)_3_, dimethyl sulfoxide (for pre-cleaning), natural or water-assisted cooling, washing with deionized water (3–6×, pH ∼7; some processes avoid washing), drying at 80–150 °C (typically overnight), grinding (manual or ball-mill), optional ultrasonication (10–30 min), post-annealing at 800–900 °C (commonly 850 °C), 3–15 h (typically 4–6 h), O_2_ or air atmosphere, Li-excess (commonly 5 mol% Li_2_CO_3_ or LiOH), multi-step calcination optional (e.g., 350→560→900 °C). | | 57.2–1537.2 $∙kg^−^¹, 58.9 $∙kg^−^¹,  0–94.3 pts | 94.0–932.1 kJ·g⁻¹,  496.1 kJ·g⁻¹,  6.8–90.6 pts | 22.6–88.9 pts  88.1 pts | ^[23–25,37,40,41]^ |  |
| NMC622, NMC532 | Li compounds-based solvothermal treatment  (Hydro-3) | 60–160 °C (commonly 140–150 °C), 6–30 h (typically 8–12 h), Li compounds (e.g., LiNO_3_, Li_2_CO_3_, LiOH), solvothermal in ethanol or ethylene glycol, Teflon-lined autoclave or sealed reactor, stirring optional (∼1 h pre-hydrothermal stirring), solid–liquid ratio ∼1:20–1:40 (e.g., 1–2 g CAM in 40–70 mL solvent), optional additives (e.g., urea, titanium butoxide), cooling natural, washing with ethanol or deionized water (3×), drying at 60–80 °C (∼6 h or freeze-drying), grinding (manual or ball-mill), post-annealing at 800–850 °C (4–12 h, O_2_ or air), Li-excess (commonly 5–10 mol% Li_2_CO_3_ or LiOH). | | 91.2–400.0 $∙kg^−^¹, 119.2 $∙kg^−^¹,  60.8–90.9 pts | 454.6–634.5 kJ·g⁻¹,  568.7 kJ·g⁻¹,  36.6–54.5 pts | 62.5–83.4 pts  77.7 pts | ^[30,34,36]^ |  |
| **Electrochemical (EC)** | | | | | | | | |
| LMO/NMC, LCO,  NMC 111, NMC 413623,  NMC 622 | Aqueous electrolytes: Li_2_SO_4_, LiNO_3_, LiOH  Organic solutions:  Lithium bromide (LiBr) in acetonitrile (MeCN),  Battery electrolytes | | Electrochemical relithiation is carried out either in static cells (e.g., H-cells) using aqueous electrolytes containing lithium salts (Li_2_SO_4_, LiNO_3_, LiOH), or in half-cells with metallic lithium as the anode. The method may involve constant current application or cyclic charge–discharge restoration with voltage and capacity control. Some techniques use suspensions of cathode powder, while others involve intact cathodes, sometimes following surface cleaning (e.g., rinsing with acetone or IPA). Alternative systems are also employed, such as LiBr in MeCN, utilizing spontaneous galvanic corrosion (with Al as a reductant), or galvanostatic deposition from Co(OH)₂ solutions in alkaline media. After relithiation, the material is often annealed at 700–800 °C to restore its crystalline structure. | 35.0–2336.1 $∙kg^−^¹, 154.2 $∙kg^−^¹,  0–96.5 pts | 0.0–433.9 kJ·g⁻¹,  94.7 kJ·g⁻¹,  56.6–100 pts | 16.8–89.2 pts  68.8 pts | ^[8,9,18,10–17]^ |  |
| **Chemical (Chem)** | | | | | | | | |
| LCO, NMC 622, LMO, NMC 811 | DTBQ, FL-Li, Per-Li, LiBr, DMSO, C_2_H_5_LiO | | Chemical relithiation is based on mild redox processes that enable the restoration of lithium content and the crystalline structure of the active material without damaging its morphology. Key approaches involve the use of redox mediators such as 3,5-di-tert-butyl-o-benzoquinone (DTBQ), lithium fluorenone (FL-Li) and lithium perylene (Py-Li), which efficiently transfer electrons and Li⁺ ions from metallic lithium to the cathode. In autooxidative systems, reagents like LiBr in dimethyl sulfoxide (DMSO) are employed, where DMSO serves not only as a solvent but also as an oxygen donor, facilitating the recovery of oxygen vacancies. Additionally, closed-loop cycles with reagent regeneration are utilized—for example, involving C_2_H_5_LiO derived from recycled lithium anodes and redox-active donors, whose chemical activity and solubility allow for repeated use in the relithiation process. | 50.7–6554.8 $∙kg^−^¹, 238.0 $∙kg^−^¹,  0–95.0 pts | 0.0–494.5 kJ·g⁻¹,  310.1 kJ·g⁻¹,  50.5–100 pts | 23.0–85.5 pts  77.5 pts | ^[1–7]^ |  |

**Table S3.** Summary of *S_Group I_* statistical robustness metrics for all regeneration methods

| **Method** | **N** | **Median** | **IQR** | **95% CI(median)** | **ΔLOO(median)** |
| --- | --- | --- | --- | --- | --- |
| **SSR (total)** | 159 | 88.36 | 6.32 | 88.26– 88.46 | 0.18 |
| SSR-1 | 100 | 88.55 | 5.17 | 88.26 – 89.53 | 0.01 |
| SSR-2 | 29 | 86.94 | 6.15 | 84.53 – 89.21 | 0.70 |
| SSR-3 | 30 | 90.04 | 11.51 | 85.74 – 91.23 | 0.03 |
| **MST (total)** | 56 | 74.09 | 32.94 | 55.52– 80.51 | 5.34 |
| MST-1 | 32 | 79.27 | 25.41 | 64.51 – 87.60 | 7.13 |
| MST-2 | 14 | 69.03 | 57.13 | 27.05 – 84.09 | 6.65 |
| MST-3 | 8 | 52.52 | 34.56 | 22.56– 59.72 | 2.71 |
| **Hydro (total)** | 61 | 88.15 | 3.92 | 87.10– 88.70 | 0.14 |
| Hydro-1 | 32 | 88.83 | 3.72 | 87.00– 89.30 | 0.19 |
| Hydro-2 | 23 | 88.15 | 2.94 | 86.96– 88.77 | 0.14 |
| Hydro-3 | 6 | 78.83 | 9.13 | 74.27– 83.40 | 4.56 |
| **EC (total)** | 20 | 71.87 | 15.99 | 67.83– 72.66 | 0.64 |
| **Chem (total)** | 30 | 77.84 | 20.97 | 63.52– 82.31 | 1.05 |
| **N** — the number of valid observations (literature data points) included in the analysis after removing invalid or non-numeric entries.  **Median** — the median of the *S_Group I_* distribution, representing the central tendency of each method with minimal influence from outliers.  **IQR (Interquartile Range)** — the difference between the third and first quartiles (Q3 − Q1), quantifying the spread of the central 50% of the data and indicating variability within each method.  **95% CI(median)** — the 95% binomial confidence interval for the median, indicating the range within which the true median is expected to lie with 95% probability. Narrow intervals reflect higher statistical stability.  **ΔLOO(median)** — the maximum change in the median under a leave-one-out sensitivity test, where extreme values are sequentially removed. This metric captures how sensitive each method’s central tendency is to dataset composition. Smaller ΔLOO values indicate greater robustness. | | | | | |

**Table S4.** Statistical robustness of Group I techno-economic criteria across relithiation methods

| **Criterion** | **Method** | **N** | **Median** | **IQR** | **95% CI(median)** | **ΔLOO(median)** |
| --- | --- | --- | --- | --- | --- | --- |
| Material costs | SSR | 159 | 96.30 | 2.51 | 96.15 – 96.41 | 0.00 |
|  | MST | 56 | 86.14 | 33.70 | 70.10 – 88.68 | 0.19 |
|  | Hydro | 61 | 94.11 | 2.18 | 93.96 – 94.28 | 0.05 |
|  | EC | 20 | 85.23 | 48.4 | 42.9 – 93.4 | 0.58 |
|  | Chem | 30 | 76.01 | 32.83 | 0.00 – 87.62 | 0.96 |
| Energy consumption | SSR | 159 | 44.03 | 19.80 | 41.6 – 50.5 | 0.52 |
|  | MST | 56 | 53.79 | 19.54 | 16.81 – 99.91 | 0.19 |
|  | Hydro | 61 | 49.52 | 16.9 | 46.1 – 54.6 | 0.21 |
|  | EC | 20 | 89.41 | 37.2 | 71.98 – 99.57 | 0.66 |
|  | Chem | 30 | 66.50 | 49.46 | 50.52 – 100.00 | 0.88 |
| Equipment cost | SSR | 159 | 60.0 | 35.3 | 58.8 – 70.7 | 0.00 |
|  | MST | 56 | 80.7 | 21.3 | 78.7 – 86.0 | 0.31 |
|  | Hydro | 61 | 78.7 | 8.0 | 78.0 – 86.0 | 0.22 |
|  | EC | 20 | 50.7 | 64.7 | 0.0 – 64.7 | 0.71 |
|  | Chem | 30 | 78.0 | 78.0 | 0.0 – 78.7 | 0.94 |
| Process duration | SSR | 159 | 86.17 | 7.63 | 85.5 – 87.5 | 0.17 |
|  | MST | 56 | 76.0 | 16.0 | 70.5 – 82.5 | 0.38 |
|  | Hydro | 61 | 81.0 | 11.0 | 75.5 – 85.5 | 0.34 |
|  | EC | 20 | 55.0 | 78.5 | 5.98 – 84.0 | 1.34 |
|  | Chem | 30 | 85.5 | 7.8 | 84.5 – 89.3 | 0.41 |
| Space requirement | SSR | 159 | 99.84 | 0.09 | 99.83 – 99.85 | 0.00 |
|  | MST | 56 | 99.72 | 0.53 | 99.57 – 99.84 | 0.01 |
|  | Hydro | 61 | 73.68 | 15.79 | 72.37 – 88.16 | 0.41 |
|  | EC | 20 | 73.68 | 83.62 | 0.00 – 98.62 | 1.0 |
|  | Chem | 30 | 94.11 | 51.84 | 72.32 – 99.21 | 0.89 |

**Fig. S1.** The example of fitting c-rates

**Table S5.** Summary of *S_Group II_* statistical robustness metrics for all regeneration methods

| **Method** | **N** | **Median** | **IQR** | **95% CI(median)** | **ΔLOO(median)** |
| --- | --- | --- | --- | --- | --- |
| **SSR (total)** | 126 | 61.13 | 29.04 | 57.37 – 66.57 | 0.45 |
| SSR-1 | 87 | 61.45 | 24.98 | 57.37 – 66.57 | 0.42 |
| SSR-2 | 22 | 60.02 | 24.85 | 53.74 – 82.36 | 0.58 |
| SSR-3 | 17 | 43.95 | 31.77 | 34.82 – 62.92 | 0.64 |
| **MST (total)** | 47 | 63.97 | 29.26 | 55.70 – 72.77 | 0.44 |
| MST-1 | 29 | 54.97 | 29.48 | 41.61 – 63.97 | 0.47 |
| MST-2 | 12 | 65.94 | 23.00 | 60.55 – 79.10 | 0.44 |
| MST-3 | 6 | 72.11 | 12.66 | 66.93 – 75.90 | 0.39 |
| **Hydro (total)** | 52 | 48.20 | 30.94 | 44.86 – 57.51 | 0.38 |
| Hydro-1 | 30 | 48.20 | 20.99 | 44.86 – 55.45 | 0.36 |
| Hydro-2 | 19 | 30.50 | 39.68 | 18.17 – 71.27 | 0.51 |
| **EC (total)** | 7 | 40.999 | 52.633 | 22.921 – 58.909 | 0.72 |
| **Chem (total)** | 11 | 57.00 | 20.98 | 49.57 – 69.32 | 0.61 |

**Table S6.** Summary of *S_Group III_* statistical robustness metrics for all regeneration methods

| **Method** | **N** | **Median** | **IQR** | **95% CI(median)** | **ΔLOO(median)** |
| --- | --- | --- | --- | --- | --- |
| SSR (total) | 159 | 45.83 | 23.01 | 43.7 – 48.1 | 2.2 |
| MST (total) | 56 | 55.18 | 29.34 | 52.3 – 57.1 | 3.1 |
| Hydro (total) | 61 | 52.61 | 18.46 | 50.20 – 55.95 | 2.5 |
| EC (total) | 20 | 78.45 | 24.10 | 72.55 – 92.91 | 4.08 |
| Chem (total) | 30 | 67.35 | 26.91 | 52.03 – 77.50 | 3.9 |

**Table S7.** Scenario-dependent median and interquartile range (Q25–Q75) of Group III scores as a function of electricity emission factor (EF)

| **EF (kg CO₂ kWh⁻¹)** | **Median** | **Q25** | **Q75** | **Median** | **Q25** | **Q75** | **Median** | **Q25** | **Q75** | **Median** | **Q25** | **Q75** | **Median** | **Q25** | **Q75** |
| --- | --- | --- | --- | --- | --- | --- | --- | --- | --- | --- | --- | --- | --- | --- | --- |
|  | **SSR** | | | **MST** | | | **Hydro** | | | **EC** | | | **Chem** | | |
| 0.00 | 99.90 | 94.63 | 91.82 | 99.98 | 99.93 | 100.00 | 99.99 | 99.95 | 100.00 | 99.51 | 99.12 | 100.00 | 99.68 | 99.63 | 99.80 |
| 0.05 | 94.17 | 89.25 | 84.10 | 94.63 | 91.82 | 94.85 | 94.36 | 93.10 | 96.00 | 97.30 | 95.93 | 98.58 | 95.86 | 94.07 | 97.97 |
| 0.10 | 88.44 | 74.27 | 55.00 | 89.25 | 84.10 | 91.70 | 88.48 | 84.10 | 90.70 | 93.60 | 91.50 | 95.90 | 91.98 | 88.36 | 94.75 |
| 0.25 | 71.25 | 99.98 | 99.93 | 74.27 | 55.00 | 80.30 | 73.45 | 65.90 | 82.50 | 84.15 | 78.75 | 92.55 | 80.33 | 71.17 | 86.87 |
| 0.417 (baseline) | 45.83 | 38.92 | 62.12 | 55.18 | 34.1 | 58.9 | 52.61 | 47.81 | 59.10 | 78.45 | 70.00 | 96.21 | 67.35 | 52.71 | 98.00 |

**Table S8.** Summary of *S_sum_* statistical robustness metrics for all regeneration methods

| **Method** | **N** | **Median** | **IQR** | **95% CI(median)** | **ΔLOO(median)** |
| --- | --- | --- | --- | --- | --- |
| SSR (total) | 126 | 65.70 | 14.45 | 62.68 – 67.86 | 0.08 |
| SSR-1 | 87 | 65.25 | 14.36 | 62.68 – 69.37 | 0.37 |
| SSR-2 | 22 | 66.40 | 8.42 | 62.49 – 68.71 | 0.11 |
| SSR-3 | 17 | 56.26 | 18.07 | 51.53 – 69.60 | 1.07 |
| MST (total) | 47 | 59.60 | 15.33 | 53.71 – 62.18 | 1.20 |
| MST-1 | 29 | 56.46 | 11.10 | 53.48 – 61.53 | 0.18 |
| MST-2 | 12 | 69.80 | 15.58 | 63.51 – 80.60 | 0.39 |
| MST-3 | 6 | 49.58 | 16.16 | 35.18 – 60.97 | 3.74 |
| Hydro (total) | 52 | 63.41 | 8.59 | 61.62 – 66.37 | 0.30 |
| Hydro-1 | 30 | 63.80 | 4.91 | 62.46 – 65.13 | 0.09 |
| Hydro-2 | 19 | 59.72 | 14.52 | 57.74 – 71.20 | 0.53 |
| EC (total) | 7 | 64.60 | 6.48 | 60.32 – 70.37 | 0.75 |
| Chem (total) | 11 | 72.27 | 6.37 | 64.59 – 74.87 | 0.15 |

**Table S9.** Summary of statistical robustness metrics for methods using LiOH/Li_2_CO_3_ as a precursor and high temperature step

| **Criterion** | **Method** | **N** | **Median** | **IQR** | **95% CI(median)** | **ΔLOO(median)** |
| --- | --- | --- | --- | --- | --- | --- |
| S_c-rate_ | Li_2_CO_3_ | 87 | 82.43 | 49.99 | 72.80 – 90.68 | 0.35 |
|  | LiOH | 22 | 93.60 | 14.54 | 85.67 – 97.17 | 0.98 |
|  | With T | 92 | 94.7 | 15.9 | 90.5 – 97.7 | 0.6 |
|  | Without T | 16 | 77.8 | 17.3 | 74.7 – 82.5 | 2.5 |
| S_cycling_ | Li_2_CO_3_ | 90 | 78.24 | 49.06 | 67.47 – 87.75 | 0.41 |
|  | LiOH | 24 | 74.57 | 26.56 | 65.59 – 83.38 | 1.46 |
|  | With T | 112 | 86.6 | 37.1 | 79.5 – 93.8 | 0.4 |
|  | Without T | 25 | 25.8 | 68.7 | 0.0 – 59.6 | ≈ 3.0 |
| S_capacity_ | Li_2_CO_3_ | 100 | 22.51 | 31.37 | 19.52 – 29.14 | 0.52 |
|  | LiOH | 29 | 27.00 | 29.99 | 16.71 – 34.29 | 2.79 |
|  | With T | 132 | 22.0 | 30.9 | 19.2 – 31.1 | 0.1 |
|  | Without T | 31 | 20.8 | 18.7 | 15.6 – 24.7 | 0.6 |
| S_Group II_ | Li_2_CO_3_ | 87 | 61.45 | 24.98 | 57.37 – 66.57 | 0.42 |
|  | LiOH | 22 | 60.02 | 24.85 | 53.74 – 82.36 | 0.58 |
|  | With T | 92 | 57.9 | 26.4 | 53.1 – 62.8 | 0.5 |
|  | Without T | 16 | 46.5 | 26.7 | 39.2 – 55.7 | ≈ 3.0 |


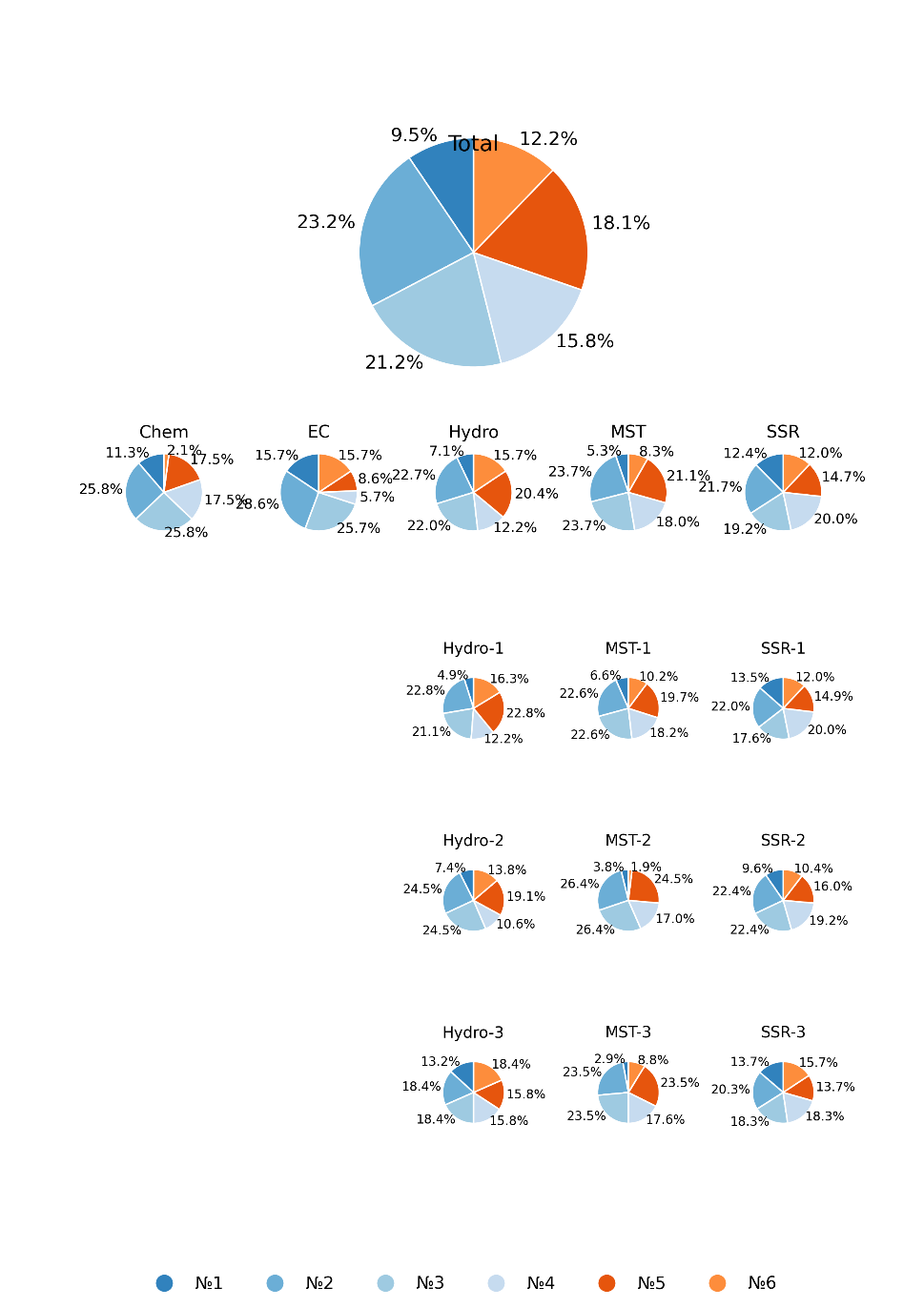


**Fig. S2.** Hierarchical pie-chart analysis of degradation mechanisms in relithiation methods


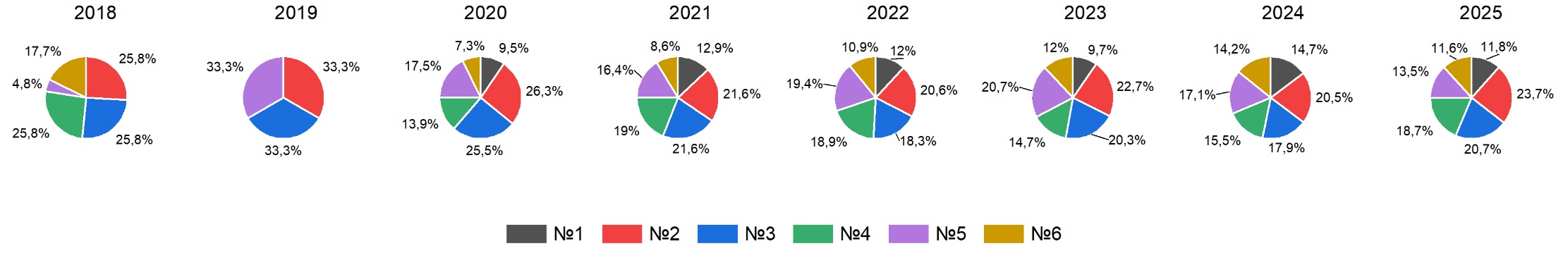


**Fig. S3.** The evolution of eliminated degradation mechanisms

**Section S1.** Justification for the selection of weighting coefficients

**Materials.** According to BatPaC ^[150]^, for NMC622 active materials account for ~67% of the cost and 38% of the mass (CAM) and an additional ~8% of the cost and 26% of the mass is accounted for by anode materials which together account for about 75% of the total cost of the cell. For PHEV50 ^[151]^ these shares are 43% (CAM) and 11% (AAM) confirming the dominance of materials. When modeling the cost structure in ^[152]^, materials accounted for 62–70% of the total cost per kWh for different form factors and chemistries. Overall, this is a typical range for the industry of 62–80% ^[150,152–155]^.

In battery recycling, materials also play a leading role in the cost of the process. The EverBatt model clearly breaks down recycling costs into “Materials cost,” “Direct labor,” “Equipment cost,” “Utilities,” “Building cost,” “Fixed charges,” etc. Materials account for a significant share of the initial costs (about 50% of all types of costs), since they determine the net revenue from recycling and a significant share of the costs of reagents and auxiliary substances ^[156]^. The study ^[157]^ indicates that material costs can reach up to ~69% of the total recycling cost, especially in hydrometallurgical schemes, where solutions, salt reagents and auxiliary liquids are used intensively. According to the study ^[158]^, the cost of materials in hydrometallurgical processes can also reach 75–90%.

**Energy.** Actual data from European production locations with a total capacity of ≈100 GWh/year show specific energy consumption of 30–35 kWh per 1 kWh of cells produced ^[159]^. With the price of electricity in the EU at around €0.20/kWh, these costs account for only ≈2–5% of the total cost of batteries. Similarly, the BatPaC  **^[150]^** and TEA ^[160]^ models show that energy costs account for only a few percent of the total cost. The study ^[153]^ confirms that materials dominate the cost structure while the share of energy is limited to 1–4 % and even with a significant increase in tariffs, the impact of energy on the total cost remains secondary. In the study ^[154]^, energy consumption varies by region (from 1.4 to 6.9% of the total cost, depending on the price of electricity and location) but even in these scenarios, energy is secondary to materials, labor and capital investments. Even in more energy-intensive pyrometallurgical processing, where temperatures reach 1400–1700 °C, materials and reagents remain the key drivers of the economy, with energy playing a secondary role ^[158]^.

**Equipment and labor.** In the EverBatt ^[156]^ and BatPaC **^[150]^** models, fixed capital investments, depreciation and labor costs together account for 15–25% of the cost of batteries. The study ^[158]^ emphasizes that capital investments and transportation are often underestimated in simplified models, but when correctly accounted for, they account for a significant share, although they still lag behind materials as a key cost driver. Similarly, analysis ^[160]^ shows that labor and capital costs together form the “second largest” group of factors after materials. Thus, our weights (0.13 for equipment and 0.10 for labor) reproduce this aggregate share and are within the ranges confirmed by both EverBatt and BatPaC.

**Area/buildings/rent.** In BatPaC, depreciation of buildings is set at around 5% of their value per year **^[150]^.** In the EverBatt model, buildings are included in capital costs, with the fixed item “building cost” also depreciated at a rate of 5% per year ^[156]^ . In study ^[152]^, dry rooms and auxiliary infrastructure are estimated at several thousand euros per m² of capital investment, which, when converted to €/kWh, contributes several percent to the product price structure. A similar order of magnitude is confirmed in the process-oriented TEA model by Orangi & Strømman ^[154]^, where “land and building” accounts for 3-7% of total costs, depending on location and scale. Similar estimates are given in the work of Wentker et al. ^[153]^ which takes into account sensitivity to land prices and capital costs for infrastructure.

The weighting structure adopted in our work is not arbitrary: materials naturally account for ~65–80% of the cost price, energy costs for only 2–5%, and labor, equipment, and space for the remaining shares. This is fully consistent with the estimates of EverBatt, BatPaC, and modern TEA studies.

**Section S2.** Toxicological hazard scoring of reagents (bm-weighting scheme)

To complement the conventional environmental metrics (e.g. CO_2_ equivalent, energy and reagent consumption), we implement a toxicity-based scoring submethod for reagents, based on the GreenScreen Hazard Benchmark concept. This scoring is intended as a *supplementary* criterion in our decision framework, particularly when only the reagent identity and its mass are known.

Method overview:

1. Benchmark assignment For each reagent (or chemical species) used in the process, we first check the public GreenScreen / Chemical Hazard Assessment (CHAD) database to see if there is an existing full GreenScreen Benchmark (BM) score ^[161,162]^. If an entry is found, we adopt that BM value (e.g. BM-1 through BM-4).
2. **Fallback to literature / analog / hazard data.** If the reagent is **not found** in these databases, we perform a targeted hazard search across public SDS / MSDS sources, regulatory databases (e.g. GHS / ECHA / US EPA / OECD), toxicological literature, and reliable chemical databases (PubChem, TOXNET, etc.). We look for key endpoints such as:

- Acute toxicity (oral, inhalation, dermal)

- Mutagenicity / genotoxicity / carcinogenicity

- Reproductive / developmental toxicity

- Chronic / systemic toxicity

- Ecotoxicity (acute and chronic aquatic)

- Persistence / biodegradation / transformation

- Bioaccumulation potential

- Physical hazards (reactivity, flammability)

We also consult analogues (chemically similar compounds) to infer hazard where direct data are absent.

1. **Benchmark determination by rules and expert judgment.**  Using the GreenScreen Benchmark Criteria (as defined in the GreenScreen Guidance v1.2 / v1.3) ^[161]^, we compare the collected hazard classifications against the decision logic for BM-1 through BM-4.

 - If any **high concern** endpoint triggers a BM-1 criterion (e.g. high carcinogenicity, PBT combining very high persistence + bioaccumulation + toxicity), the chemical is assigned BM-1.

 - Otherwise, we evaluate whether it meets criteria for BM-2, BM-3, or BM-4 in descending order.

1. Numeric score scaling. We associate each BM level with a numerical score S_BMs_ ​ on a 0–100 scale such that higher scores correspond to lower hazard (i.e. BM-1 = 0.00, BM-2 = 33.33, BM-3 = 66.67 and BM-4 = 100.00 points).
2. Weight coefficients by mass share. Let *m_i_* ​ be the mass of reagent *i*. We define the weight coefficient:

| $w_{i}=\frac{m_{i}}{\sum_{j} m_{j}}$ | (1) |
| --- | --- |

1. Process-level toxicological score. The overall reagent-toxicity score for the process is computed as:

| $S_{tox}=\sum_{i} w_{i}\cdot s_{BM, i}$ | (2) |
| --- | --- |

This S_tox_ ​ takes values in [0, 100]. A higher S_tox_ ​ implies a reagent composition with relatively lower inherent hazard.

1. Interpretation and limitations. This metric is a *hazard-based, mass-weighted proxy* and does not replace full toxicological risk assessment (which would require exposure, fate, transformation, etc.). However, it allows comparative ranking of alternative reagent sets under the constraints of limited data. In the Discussion section we highlight the caveats and recommend that this score be used as a supportive indicator rather than a deterministic decision criterion. In practice, we compute S_tox_ for each alternative processing scenario and compare it alongside traditional metrics (CO₂ emissions, water footprint, reagent cost) to assess trade-offs.
2. Results and Discussion. The comparative toxicological assessment highlights significant contrasts among relithiation strategies (Fig. S3, Table S1). Overall, the median Sₜₒₓ values for SSR, MST, Hydro and EC methods converge near 33.3 (BM-2), representing a moderate hazard level.
   In contrast, **Chem** processes exhibit a lower median around 22 (BM-1–BM-2), suggesting the use of more aggressive reagents and solvents. SSR routes generally rely on Li₂CO₃ or LiOH and operate without organic solvents or strong oxidants. Their toxicity profile is dominated by the basicity and thermal reactivity of these inorganic salts. Consequently, most SSR processes fall within BM-2, with minimal environmental or toxicological concern aside from dust generation and caustic residue formation. MST methods show a similar median Sₜₒₓ but a broader distribution due to the diversity of flux chemistries. Compositions containing nitrate or halide eutectics tend toward lower benchmarks (BM-1–BM-2) because of oxidative or corrosive potential, whereas carbonate- and hydroxide-based media shift the benchmark upward (BM-3). Thus, MST toxicity is largely determined by the redox stability of the chosen flux. Hydrothermal techniques employ concentrated aqueous or mixed organic–aqueous Li-salt solutions (commonly LiOH, Li₂CO₃, or LiNO₃). The main toxicological factors originate from alkaline corrosion and the toxicity of metal nitrates, ammonia derivatives, or organic solvents occasionally introduced to promote crystal reconstruction. Chemical methods of re-lithiation demonstrate the lowest Sₜₒₓ values values (≈ 22; BM-1–BM-2), reflecting the high hazard of reactive organic lithium compounds, redox mediators, and polar aprotic solvents, which are volatile, flammable, and harmful to aquatic organisms. Thus, chemical relithiation is currently the most aggressive method from a chemical point of view, but it is universal in composition and requires further development of more environmentally friendly analogues of mediators and solvents to achieve BM-3 or BM-4 safety levels. Electrochemical methods show moderate-to-high Sₜₒₓ values (BM-2–BM-3), largely governed by the electrolyte composition. Aqueous electrolytes based on Li₂SO₄, LiOH, or LiNO₃ represent the majority of systems and contribute only limited toxicity after neutralization. Non-aqueous variants, such as LiBr in acetonitrile, introduce additional hazards due to solvent volatility and halide content, slightly lowering the benchmark. Overall, EC relithiation maintains medium-level hazard comparable to hydrothermal routes, combining manageable reagent toxicity with minimal emissions and good recyclability of electrolytes.

**Fig. S4.** Results of relithiation methods toxicological assessment
